# Mapping 60 Years of Discovery: An AI‐Driven Bibliometric and Altmetric Analysis of the *Journal of Periodontal Research*

**DOI:** 10.1111/jre.70071

**Published:** 2025-12-30

**Authors:** Parham Hazrati, Tara Zibandehkhooy, Hamoun Sabri, Mario Romandini, Shayan Barootchi

**Affiliations:** ^1^ Department of Periodontics and Oral Medicine University of Michigan School of Dentistry Ann Arbor Michigan USA; ^2^ Periodontics and Implantology Advanced Computing and Artificial Intelligence (PICO.AI) Ann Arbor Michigan USA; ^3^ School of Business, Stevens Institute of Technology Hoboken New Jersey USA; ^4^ Center for Clinical Research and Evidence Synthesis in Oral Tissue Regeneration (CRITERION) Ann Arbor Michigan USA; ^5^ Ninth People's Hospital, Shanghai Jiao Tong University School of Medicine Shanghai China; ^6^ Department of Oral Medicine, Infection, and Immunity, Division of Periodontology Harvard School of Dental Medicine Boston Massachusetts USA

**Keywords:** artificial intelligence, Bibliometrics, ChatGPT, dental implants, evidence‐based dentistry, generative artificial intelligence, large language models, natural language processing, periodontal diseases, periodontics

## Abstract

AI, particularly the large language model GPT‐5‐mini, demonstrated reliable performance in labeling research topics and levels of evidence. Over the past 60 years, *JPR* has made a substantial contribution to the periodontal literature, generating meaningful public and societal impact, and it is expected to experience exponential growth in the coming years.

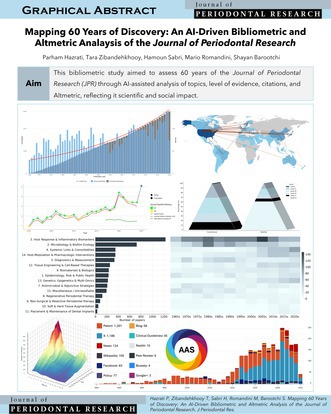

## 1 Introduction

Since its establishment in 1965, the *Journal of Periodontal Research* (*JPR*) has played a central role in shaping periodontology as a modern scientific discipline and is recognized as one of the oldest and most influential journals in dentistry. Professor Harald Löe, one of the fathers of modern periodontology and the founder of *JPR*, envisioned transforming periodontology from a primarily clinical discipline into a globally recognized, research‐driven field [1]. In contrast to the clinically oriented publications of that era, which emphasized practical advice and region‐specific issues, the journal was established to advance scientific investigation in periodontology and to position the discipline as a rigorous, research‐driven specialty on a global scale [2].

Over the past six decades, Professor Löe's vision has been realized, with *JPR* contributing significantly to a culture and tradition of inquiry, discovery, and advancement that has propelled the discipline toward an evidence‐based, literature‐supported contemporary practice, while also becoming one of the most impactful journals in periodontology and dentistry as a whole. To mark its 60‐year milestone, *JPR* has introduced several celebratory initiatives. These include the transition to 12 issues per year, reflecting the journal's growth and a commitment to publishing the latest periodontal research with greater frequency and accessibility, the launch of a new cover design [3], the commemorative series “The Past, The Present, The Future” [2], and this special issue honoring the journal's founding editor, Professor Harald Löe.

Similar to periodontics itself, *JPR* has evolved through the ages, adapting to the needs and interests of researchers and clinicians in each period. Starting with investigations on the etiopathology of periodontitis, moving to regeneration in periodontology, and later implant dentistry, today *JPR* addresses periodontal applications of cutting‐edge technologies such as artificial intelligence (AI), genetics, biologics, and photomedicine [2, 4, 5, 6]. Despite this wide range of published studies and covered topics, no bibliometric study has yet thoroughly examined this longstanding journal, which can serve as a valuable source for historical perspective and future opportunities for both the journal and the specialty.

In this context, this study aims to provide a comprehensive bibliometric analysis of its publication history, focusing on trends in topics, levels of evidence (LEVs), and key bibliometric indices. In line with the journal's longstanding tradition of embracing innovation and promoting the integration of emerging technologies into periodontology, the present study was complemented by the incorporation of AI and Altmetrics. Specifically, AI was applied to categorize the body of publications into research topics and levels of evidence, a task conventionally performed manually by experts in the field in previous bibliometric reviews [7, 8, 9]. While bibliometric studies exploring AI applications in healthcare are abundant [10, 11], the use of AI as a methodological tool within bibliometric research remains limited and is virtually absent in dental research [12]. By doing so, this study not only highlights the scholarly influence of *JPR* but also illustrates the opportunities and challenges of applying AI in bibliometric analysis.

## 2 Methods

### 2.1 Protocol and Study Design

This study was conducted as a cross‐sectional bibliometric review and Altmetrics analysis, and in line with the recommendations of the Enhancing the QUAlity and Transparency of Health Research (EQUATOR) Network, it was carried out in full accordance with the Preliminary Guideline for Reporting Bibliometric Reviews of the Biomedical Literature (BIBLIO) [13]. Given its bibliometric nature, this study was deemed exempt from institutional review board and ethical approval requirements.

### 2.2 Search Strategy and Data Sources

At the initial stage, all databases available in Web of Science (available online at: https://www.webofscience.com), including the Web of Science Core Collection and MEDLINE, were searched using the query “JOURNAL OF PERIODONTAL RESEARCH (Publication/Source Titles)” on June 1, 2025, to export all available bibliometric information, abstracts, and citation data, including both total citation counts (TCCs) and yearly citation counts (YCCs), for all research articles published across the entire publication history of the journal (1966–2025); nevertheless, YCC data was available since 1972. Web of Science was chosen because it provides exportable YCC and standardized citation metadata, enabling robust longitudinal analyses of citation dynamics, time‐series analyses of citation trajectories, and period‐specific topic salience. No restrictions or filters were applied to the search query, resulting in the inclusion of all available records, including early view articles, referring to online publications made available prior to their inclusion in an issue. After data export, the identified citations were cross‐checked against the journal's official online archive (available at: https://onlinelibrary.wiley.com/loi/16000765; last accessed June 1, 2025), which served as the primary reference for inclusion and exclusion. Also, retractions and corrections were excluded from the database. Finally, the Altmetric database was searched with the query “Exploring data for all research outputs from the full Altmetric database published in the *Journal of Periodontal Research* between 1966‐01‐01 and 2025‐06‐01”. Similarly, no filters or restrictions were applied in Altmetric search.

### 2.3 Overall Journal Trends and Metrics Analysis

After finalizing the dataset, total number of documents, document average age, total number of citations, average citation per document, total number of references, total number of authors, average number of authors per document, number of single‐authored documents, and international co‐authorship were retrieved from the dataset. Additionally, the following metrics were calculated from the dataset including individual document‐level data of all the publications:

- *Compound Annual Growth Rate (CAGR)*: This rate represents the mean annual growth rate of a measure over a specified time period, assuming the value grows at a steady, compounded rate each year. In other words, it answers the question: “If the measure had grown at the same rate every year, what would that rate have been to reach the final value from the initial value?” The CAGR is useful as it smooths out year‐to‐year fluctuations and provides a single growth rate that is easy to compare across different measures or time frames. It is calculated using the following formula:

CAGR=Final valueInitial value1years

The CAGR was calculated for publication count. At the time this study was conducted, the 60th volume of *JPR* had not been finalized; therefore, the 59th volume published in 2024 was considered the latest volume, and the 2024 publication count was used as the final value. Early View articles published in 2024 but not assigned to an issue were therefore excluded solely from this analysis.

- *H‐Index*: This index is the largest number h such that at least h of a journal's publications have each been cited at least h times. For example, for *JPR*, an h‐index of 133 means there are 133 documents with ≥ 133 citations each. Like for individual researchers' h‐index, it serves as a cumulative measure of both the journal output and citation impact.

Finally, internal data regarding the number of submissions, full‐text views, readers' countries, decision time, acceptance rate, and the number of peer‐reviews were obtained from Wiley for the period 2021–2025 (with 2025 updated to October 31).

### 2.4 Journal Impact Factor and Performance Assessment

Citation statistics for the journal were obtained from the latest release of Journal Citation Reports (JCR; Clarivate; accessed on June 1, 2025). The following journal‐level metrics were extracted:

- *Journal Impact Factor (JIF)*: Defined as the number of citations in year *Y* to citable items published in *Y–1* and *Y–2*, divided by the number of citable items published in *Y–1* and *Y–2*. For example, the JIF of *JPR* in 2024 is calculated with the following formula, resulting in 3.4.

$$\frac{\text{Citations in}\;2024\;\text{to papers published in}\;2023\;\left(383\right)+\text{Citations in}\;2024\;\text{to papers published in}\;2022\;\left(391\right)}{\text{Number of papers published in}\;2023\;\left(125\right)+\text{Number of papers published in}\;2022\;\left(106\right)}$$

The projected 2025 JIF (expected to be officially released in June 2026) was estimated using a ratio‐based approach, calculated from the actual 2025 citations indexed in the Web of Science to articles published in 2023 and 2024, normalized over a 12‐month period and adjusted for the typical 2 to 4‐week citation indexing delay.

- *Journal Citation Indicator (JCI)*: Introduced in 2021 and back‐calculated to 2017, this metric represents the mean category normalized citation impact (CNCI) of a journal's citable items (articles and reviews) over a recent three‐year window. A JCI score of 1 corresponds to the category average (for *JPR*: “Dentistry, Oral Surgery & Medicine”), and a JCI of 1.5 indicates 50% higher citation impact than the category average.
- *Quartile rank by JIF*: Represents the journal's position within its JCR category based on JIF, reported as Q1 (top 25%), Q2 (50th–75th percentile), Q3 (25th–50th percentile), or Q4 (bottom 25%).

### 2.5 Topic and Level of Evidence Assignment

Following an initial assessment of a randomly selected, year‐stratified pool of articles and consideration of the general themes and major areas of research in periodontics, 15 topics were developed through authors' discussion and consensus to categorize all studies. Initially, 20 topics were proposed; however, to reduce overlap between thematic areas and make the inclusion and exclusion criteria more precise and decisive for AI classification, they were consolidated into 15 by merging closely related categories. One category, labeled “Miscellaneous”, was retained to include studies that did not fit clearly into any of the predefined topics. These topics included: (1) Epidemiology, Risk, and Public Health, (2) Microbiology and Biofilm Ecology, (3) Host Response and Inflammatory Biomarkers, (4) Systemic Links and Comorbidities, (5) Diagnostics and Measurement, (6) Nonsurgical and Resective/Access Periodontal Therapy, (7) Antimicrobial and Adjunctive Strategies, (8) Regenerative Periodontal Therapy, (9) Biomaterials and Biologics, (10) Soft and Hard Tissue Augmentation, (11) Placement and Maintenance of Dental Implants, (12) Tissue Engineering and Cell‐Based Therapies, (13) Genetics, Epigenetics, and Multi‐Omics, (14) Host Modulation and Pharmacologic Interventions, and (15) Miscellaneous.

**FIGURE 1:**
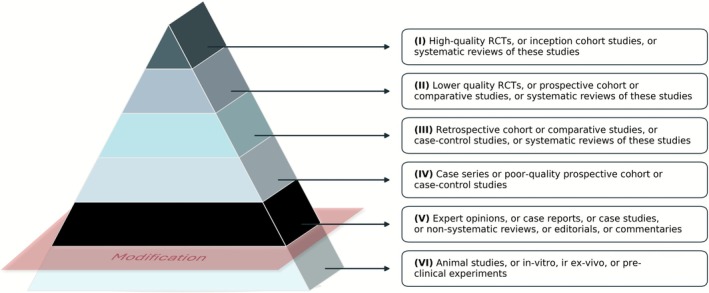
Oxford Centre for Evidence‐Based Medicine (OCEBM) 2011 Levels of Evidence (LEV) scale, with the modification of addition of a Level VI (Abbreviations: RCT: Randomized Controlled Trial).

LEV was mapped using the Oxford Center for Evidence Based Medicine (OCEBM) 2011 scale [14]. The scale is structured into five levels (I–V) according to the study design and quality. In line with previous studies [8], an additional level (VI) was introduced to classify preclinical research, including animal, in vitro, and ex vivo studies to accommodate a considerable body of evidence not investigating human participants (Figure 1). Two detailed codebooks including definitions and inclusion and exclusion criteria were developed for topics and LEV (Appendix S1).

Three OpenAI (San Francisco, CA, USA) large language models (LLMs), GPT‐5‐mini, GPT‐4o‐mini, and GPT‐4o, were accessed through the OpenAI application programming interface (API) and prompted via the codebooks to label a pilot pool of 250 studies that were randomly selected from the database. Each model labeled both Topic and LEV of these studies using only the title, abstract, and author keywords as input text. To ensure consistent outputs, a single deterministic prompting setup was used. Temperature was set to 0 for GPT‐4 and GPT‐4o‐mini, and the fixed snapshot version (gpt‐5‐mini‐2025‐08‐07) was used for GPT‐5‐mini to guarantee reproducibility. The same set of pilot studies was also labeled for both topic and LEV by an expert (H.S.) blinded to author(s) and year of publication. Inter‐rater agreement between the human expert and each AI model was evaluated using Cohen's κ statistic implemented in the *irr* package in RStudio (Version 2025.05.0 + 496, Posit Software, PBC). For topic classification (15 nominal categories), unweighted κ values were computed using the kappa2 function. However, for LEV classification (six ordinal categories), Cohen's weighted κ with quadratic weights was applied to account for the magnitude of disagreement between categories. For both topic and LEV GPT‐5‐mini demonstrated significantly superior agreement with human expert compared to other models (Topic: GPT‐5‐mini = 0.71, GPT‐4o‐mini = 0.64, GPT‐4o = 0.59; LEV: GPT‐5‐mini = 0.77, GPT‐4o‐mini = 0.65, GPT‐4o = 0.71). The agreement for topic and LEV was rated as good and excellent, respectively [15]. On the basis of these results, the pilot evaluation validated the reliability of the automated classification, supporting AI model use for labeling the full dataset.

Finally, for the entire dataset, each codebook was separately incorporated into the prompt used via the GPT‐5‐mini (2025‐08‐07) API to assign topics and LEVs to 10 batches of randomly selected studies, each batch including 468 studies in two different sessions. Further technical details, including prompt structure and parameter configuration, are provided in Appendix S2.

### 2.6 Top Cited Publications

The full, cleaned dataset of all published papers exported from Web of Science was sorted by TCC, and the top 100 most‐cited papers were identified. Also, average citations per year (ACY) were calculated by dividing TCC by the age of the paper. The age value was defined as the number of years since the article's first online publication (e‐publication) date, such that papers published in 2024 had an age value of 1, those in 2023 had an age value of 2, and so forth. For these papers, titles, publication years, and authorship information were exported for reporting. Also, the papers published in 2025, whether assigned and finalized or still in the early view stage, were assigned an age of 0 and therefore were excluded from this analysis.

### 2.7 Author, Country, and Institution Rankings, Clusters, Research Interests, and Collaborations

Top authors, countries, and institutions in terms of number of publications were identified in the dataset. Also, author clusters and collaboration network analysis were performed with VOSviewer (version 1.6.17, Leiden University Center for Science and Technology Studies, Leiden, The Netherlands) [16]. For enhanced readability, network analysis between authors was performed on authors with at least 20 publications, also excluding papers with more than 10 authors. Moreover, international collaboration network analysis was performed with ‘bibliometrix’ (Version 5.1.1.) in RStudio (Version 2025.05.0 + 496, Posit Software, PBC) [17]. To evaluate whether the distribution of research topics differed among countries, the relative frequency of each topic among publications with at least one author from each of the five most prolific countries was calculated and assessed using a Chi‐square test of independence for inter‐country differences. Following a significant Chi‐square outcome, standardized residuals were computed to identify the specific country–topic combinations contributing most to the deviation from independence. Due to the relatively large sample size and the resulting approximation to a standard normal distribution, residuals (*r*) greater than +2 or less than −2 (≈|1.96|) were considered indicative of higher or lower than expected frequencies, respectively, at the 5% significance level (two‐tailed). Positive residuals therefore denoted overrepresentation of a topic within a country's publications, while negative residuals denoted underrepresentation. All computations were performed in RStudio.

### 2.8 Funding Analysis

Funding information, including funding status and funder names, was retrieved from the database to identify leading sources of support for publications in *JPR*. “NA” or missing entries in the funding acknowledgments were interpreted as absence of funding and were treated as no funding status. The 100 most frequently acknowledged funders were tabulated, and summary statistics on funding across the included papers were compiled.

### 2.9 Altmetric Analysis

The Altmetric database (Altmetric, London, United Kingdom; available online at: www.altmetric.com) was used to retrieve Altmetric data. Using the advanced search function, the query mentioned in section 2.2. was formulated and used, and all identified publications along with their associated Altmetric data were exported into a spreadsheet format. No restriction or filter was applied to publication year or publication status. The dataset included the Altmetric Attention Score (AAS), a metric calculated through a proprietary algorithm that weights the amount and type of online attention a research output receives across multiple sources and platforms, such as patents, X (formerly Twitter), Facebook, LinkedIn, YouTube, blogs, and news outlets. The AAS is typically represented by the Altmetric “donut”, where each color corresponds to a specific online source. Additionally, chronological and geographical distribution of attentions across all sources and platforms were exported. The top articles based on AAS and average AAS per year (AAASY) were identified using the same procedure applied for determining the top‐cited articles according to TCC and ACY, respectively. The linear correlation between TCC and the AAS, as well as source‐specific mention counts, was evaluated using the Pearson correlation coefficient in RStudio. Correlation strength was interpreted based on the absolute *r* value, with *r* < 0.1 considered very weak, 0.1–0.3 weak, 0.3–0.5 moderate, 0.5–0.7 strong, and 0.7–1.0 very strong [18]. Also, statistical significance was set at 0.05.

## 3 Results

### 3.1 Journal's Overall Metrics

**FIGURE 2:**
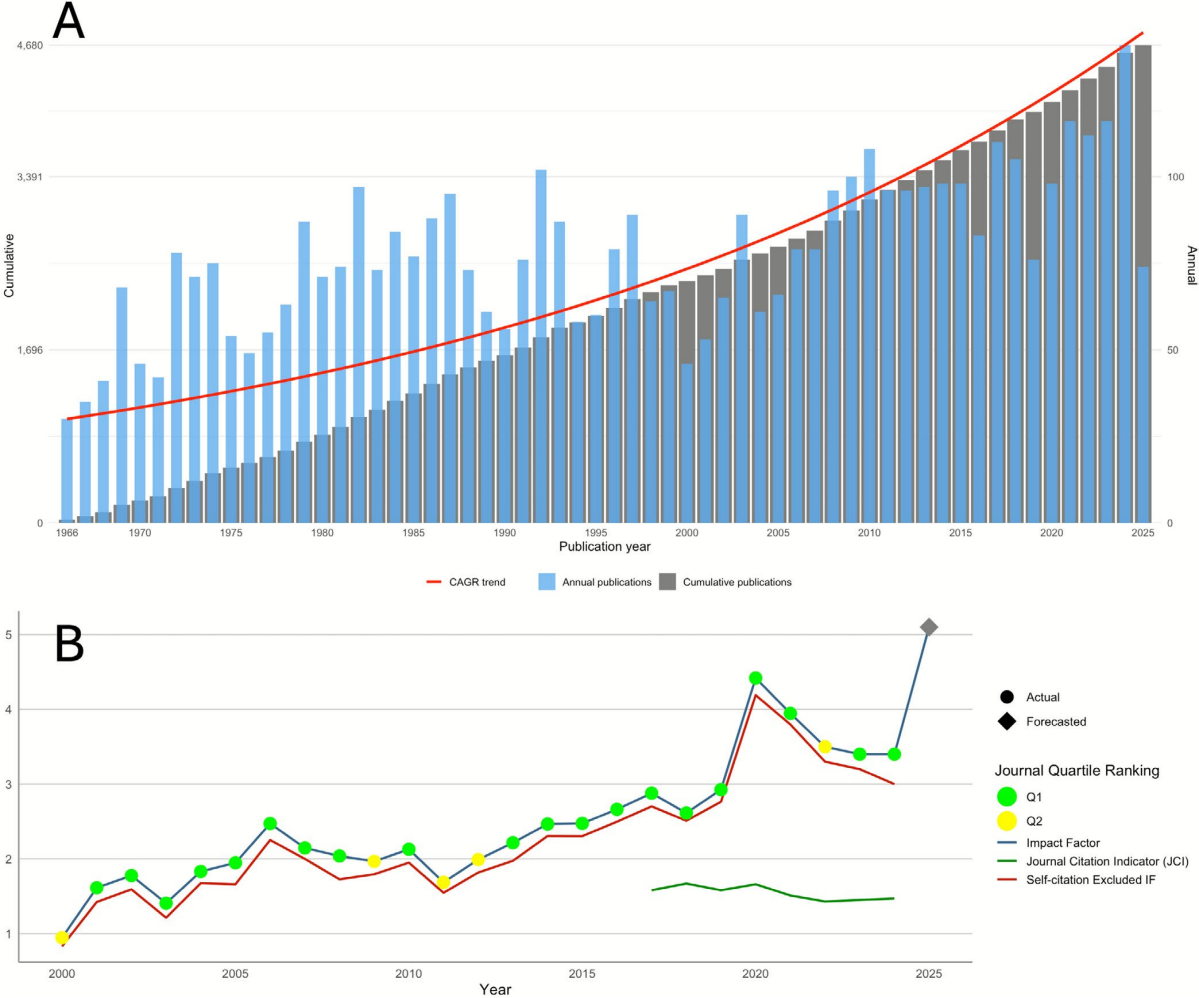
(A) Annual and cumulative publication count of *JPR* through 1966 to June 1, 2025, along with the compound annual growth rate (CAGR) trend; (B) Journal impact factor (JIF), Journal Citation Indicator (JCI) and quartile ranking according to JIF trends from 2000 to 2025. The value shown for 2025 is forecasted and may be subject to minor variation.

Over its 60‐year history (1966–2025), *JPR* has published 4680 research papers. The first article, also the most‐cited paper, is “Experimental gingivitis in man. II. A longitudinal clinical and bacteriological investigation” by Theilade et al. 1966 [4]. The journal was most productive in 2024, publishing 138 documents, while it only published 30 documents in its introductory year, 1966. Figure 2A shows the annual and cumulative publication count of *JPR* by June 1, 2025, along with the 2.62% CAGR. The average age of documents was 26.14. Over this period, the journal's publications cited 162 387 references (mean, 34.7 per document). In the same timeframe, *JPR* articles received 147 283 citations overall, averaging 31.47 citations per document. The journal had an H‐Index of 133. Table 1 presents the general metrics and characteristics of the journal.

**TABLE 1:** General metrics of the *Journal of Periodontal Research* until June 1, 2025.

Description	Results
Timespan	1966:2025
Total number of documents	4680
CAGR	2.62%
Document average age (year)	26.14
Total number of citations	147 283
Average citation per document	31.47
Total number of references	162 387
Average number of references	34.7
Total number of authors	10 876
Average number of authors per document	4.55
Single‐authored documents	476
International co‐authorship	16.62%
Impact factor (2024)	3.4
H‐index	133
Issues per year	2025 (continuing): 12
	2000–2024: 6
	1996–1999: 8
	1994–1995: 6
	1993: 7
	1973–1992: 6
	1966–1972: 4

Abbreviation: CAGR, compound annual growth rate.

**FIGURE 3:**
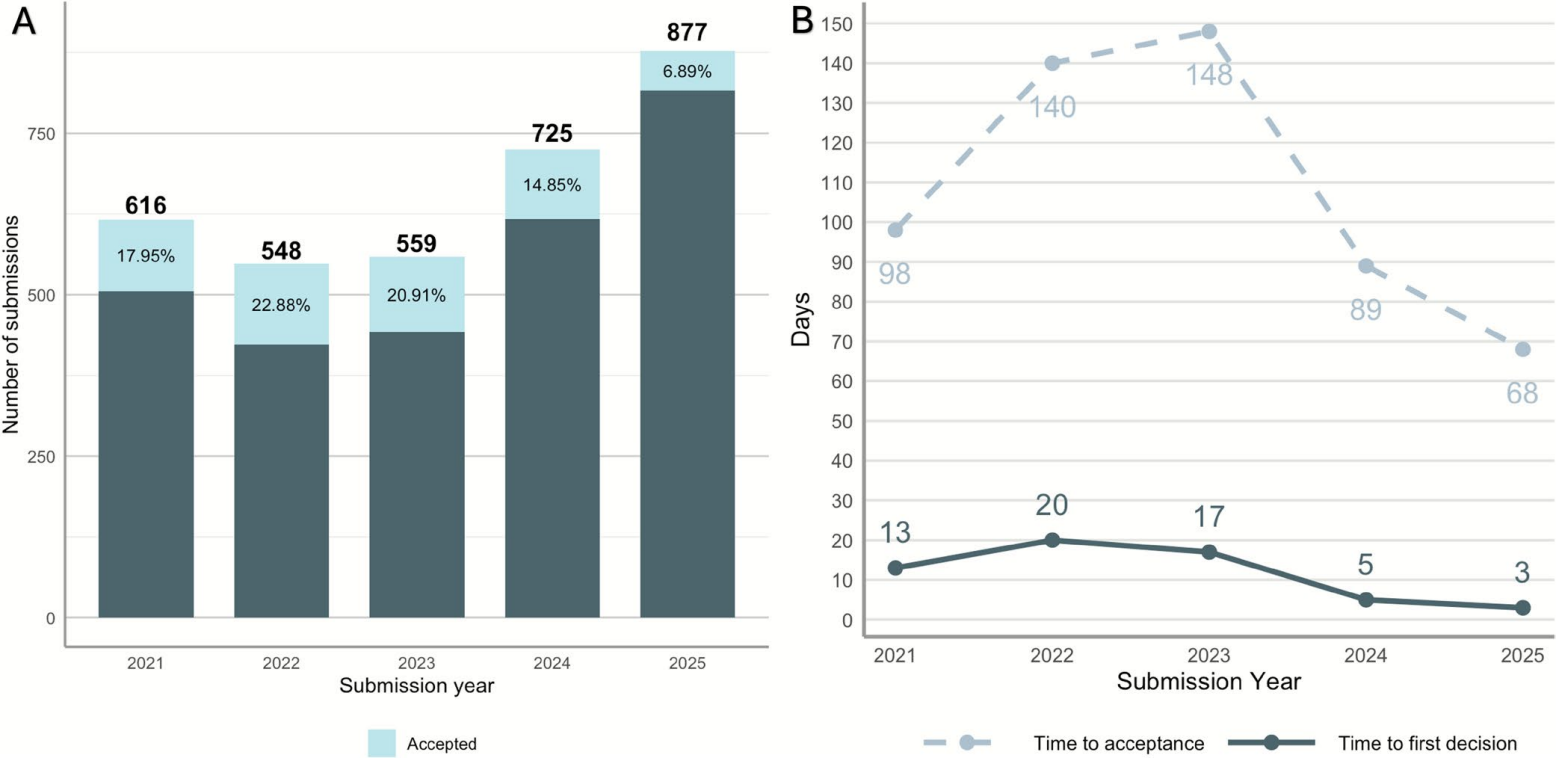
(A) Number of submissions along with acceptance rate and (B) average time to first decision and acceptance from 2021 to October 31, 2025.

In 2025, up to the end of October, 877 submissions were made to *JPR*, 6.89% of which were accepted for publication (Figure 3A). This represents a 32.08% increase in submission count compared to the same period in 2024 and aligns with the journal's overall upward trend. In addition, both the time to first decision and the time to acceptance decreased substantially in 2024 and 2025 (Figure 3B). In 2025, the average time to first decision and acceptance are 3 and 68 days, respectively, while in 2023 the same times corresponded to 17 and 148 days, respectively.

**FIGURE 4:**
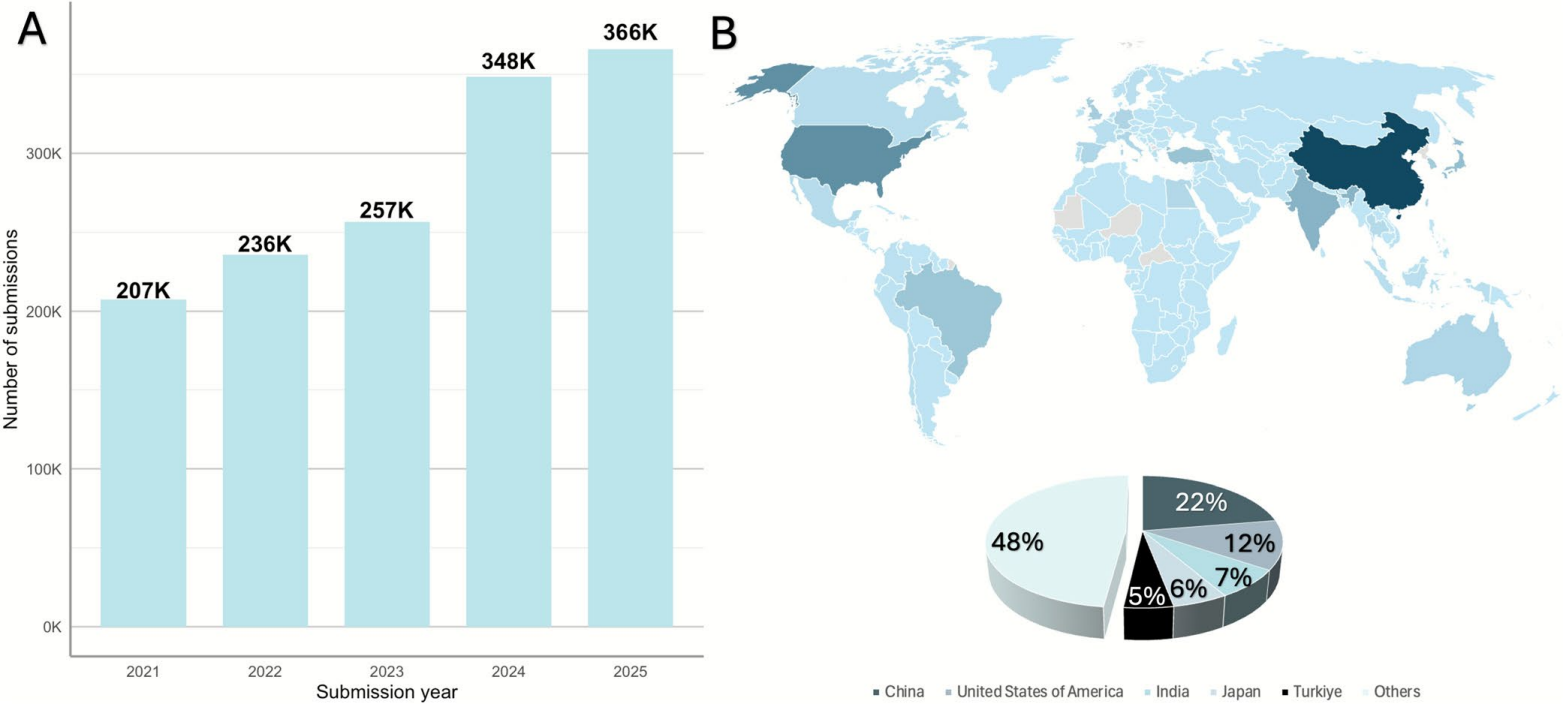
(A) Number of full text reads from 2021 to October 31, 2025; (B) Geographical distribution and the top 5 countries of full text reads in 2025 until October 31.

By October 31, *JPR* publications had collectively received 366 184 full‐text reads in 2025. Since 2021, the number of full‐text reads has steadily increased from 207 073 (2021) to 366 184 (2025, until October 31) (Figure 4A). The leading countries for full‐text reads in 2025 (until October 31) were China (22%), the United States (12%), India (7%), Japan (6%), and Türkiye (5%) (Figure 4B). Notably, China, the United States, and Japan consistently ranked as the top three countries in the same order from 2021 through October 31, 2025. As of October 31, 2025, among the 1403 peer‐review invitations sent, 1139 have been accepted and completed (Table S1).

### 3.2 Journal's Impact Factor: Past Trends and Future Forecast

The journal entered the 21st century with a JIF just under 1.0 (0.946). As depicted in Figure 2B, JIF rose overall until 2005. From 2005 to 2011, it declined modestly to 1.686. From 2011 onward, the JIF increased steadily, reaching its historical maximum in 2020 (4.419), and since 2020, it has remained above 3. A forecast based on the current 2025 citation data of articles published in 2023 and 2024 suggests that the 2025 JIF (to be released in June 2026) is expected to be approximately 5.1, although this estimate remains tentative. Moreover, *JPR* articles first published online during 2025 have so far, until October 31, 2025, received an average of 1.54 citations per citable item within the same year—higher than those of other leading journals in the field—indicating that the JIF is likely to continue growing substantially in the coming years.

Over the past quarter century, the journal was outside Q1 in only 5 years (2000, 2009, 2011, 2012, and 2022). Also, in the past decade, it has been ranked Q1 in 9 of 10 years. Since 2017, the JCI has hovered around 1.5 (ranging from 1.43 in 2022 to 1.67 in 2018), indicating that articles in *JPR* have about 50% more citation than the average literature of periodontology.

### 3.3 Topic Analysis

**FIGURE 5:**
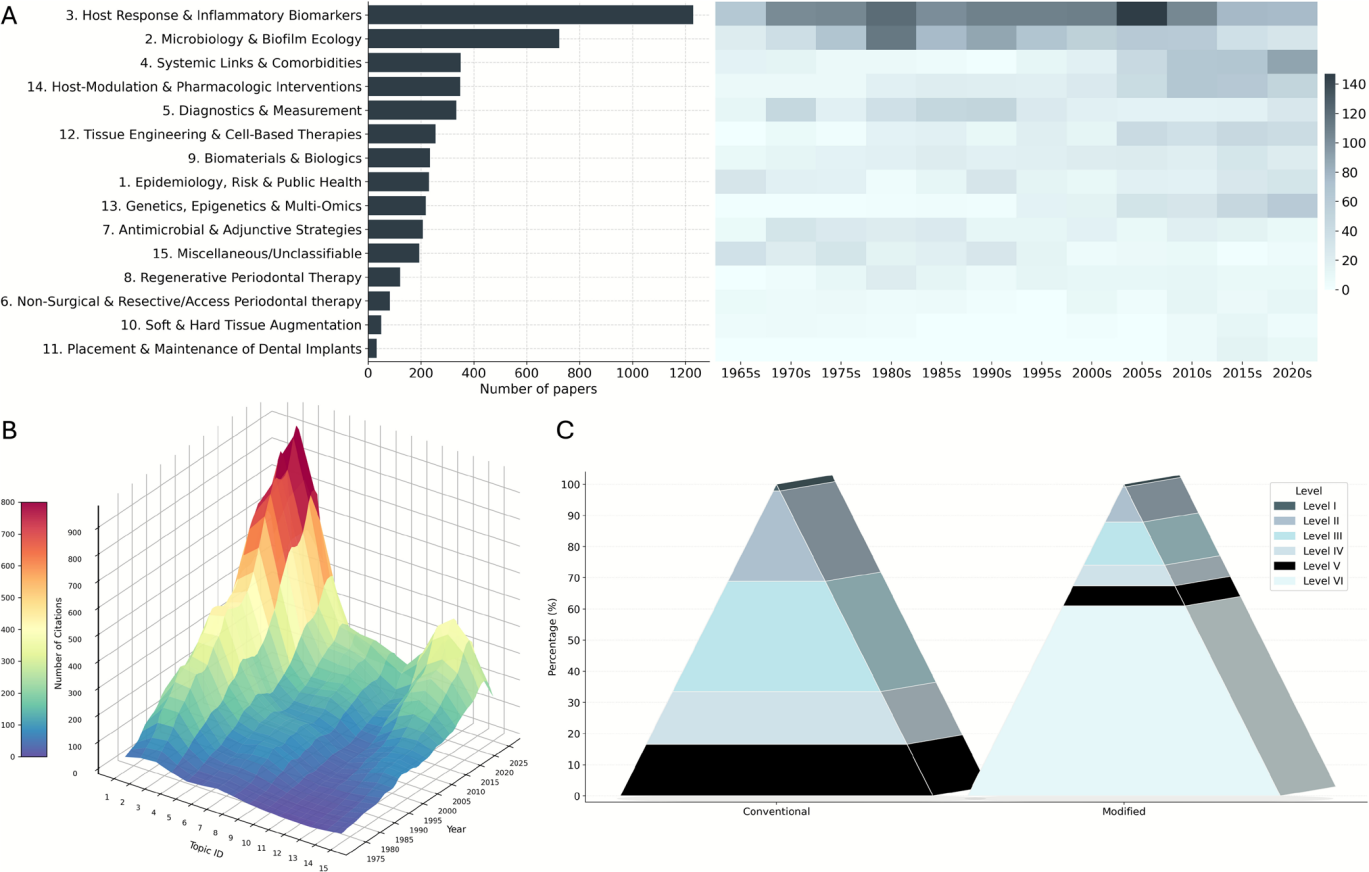
(A) Horizontal bar plot and heatmap presenting frequency and chronological distribution of topics from 1966 to June 1, 2025. (B) Distribution of yearly citation count of each topic through 1972 to June 1, 2025. (C) Level of evidence pyramids (Left) excluding level VI (in vitro, ex vivo, animal, and pre‐clinical studies) and (right) including level VI. (Topic ID: (1) Epidemiology, Risk, & Public Health; (2) Microbiology & Biofilm Ecology; (3) Host Response & Inflammatory Biomarkers; (4) Systemic Links & Comorbidities; (5) Diagnostics & Measurement; (6) Non‐Surgical & Resective/Access Periodontal Therapy; (7) Antimicrobial & Adjunctive Strategies; (8) Regenerative Periodontal Therapy; (9) Biomaterials & Biologics; (10) Soft & Hard Tissue Augmentation; (11) Placement & Maintenance of Dental Implants; (12) Tissue Engineering & Cell‐Based Therapies; (13) Genetics, Epigenetics, & Multi‐Omics; (14) Host‐Modulation & Pharmacologic Interventions; (15) Miscellaneous.)

Nearly one‐fourth of all publications (> 1200 documents; 26.39%) were categorized as “Host‐Response and Inflammatory Biomarkers”, the most common topic over the past 60 years (Figure 5A and Table S2). The next most common topics were “Microbiology & Biofilm Ecology” (15.56%), “Systemic Links and Comorbidities” (7.63%), “Host Modulation & Pharmacologic Interventions” (7.59%), and “Diagnostics & Measurement” (7.24%). The least represented were “Placement & Maintenance of Dental Implants”, “Soft & Hard Tissue Augmentation”, and “Non‐surgical & Resective/Access Periodontal Therapy”. As shown in Figure 5A, “Host‐Response and Inflammatory Biomarkers” remained among the leading themes across decades, peaking most intensely in the second half of 2000s. “Host Modulation & Pharmacologic Interventions” reached its strongest period between 2010 and 2020. “Microbiology & Biofilm Ecology” peaked in the first half of 1980s and continued among peak topics until 2015. “Systemic Links and Comorbidities” began rising in the late 1990s and is currently at its peak, a pattern mirrored by “Host Modulation & Pharmacologic Interventions”. In contrast, “Diagnostics & Measurement” peaked from 1986 to 1995 and has declined in recent years. “Genetics, Epigenetics & Multi‐Omics” has grown steadily since 2000 and is now at a historical peak.

As shown in Figure 5B, most topics exhibited marked growth in citation counts over the past 53 years. Consistent with their larger publication volumes, “Host‐Response and Inflammatory Biomarkers”, “Microbiology & Biofilm Ecology”, and “Systemic Links and Comorbidities” showed the steepest increases in YCC. In contrast, “Soft & Hard Tissue Augmentation” displayed the smallest gains. Since early 2000s, “Tissue Engineering & Cell‐Based Therapies” and “Genetics, Epigenetics, & Multi‐Omics” are increasingly being published and cited. Table S3 reports YCC for each topic from 1972 onward.

### 3.4 Level of Evidence (LEV) Analysis

LEVs were summarized using two complementary schemes (Figure 1). With a six‐level framework that added a preclinical category, most papers fell into Level VI (60.96%), with the remainder classified as I 0.83%, II 11.32%, III 13.8%, IV 6.6%, and V 6.47%. As depicted in Figure 5C, when recalculated with the conventional five‐level scale (I–V), the distribution shifted to I 2.13%, II 29.01%, III 35.36%, IV 16.91%, and V 16.58%. In some categories, including “Host Modulation & Pharmacologic Interventions”, “Genetics, Epigenetics & Multi‐Omics”, and “Biomaterials & Biologics” level VI had almost 90% or more frequency. Supplementary Table 2 presents the detailed frequency and count of each level across the topics.

### 3.5 Most Impactful Papers

Based on TCC, the 100 most‐cited articles were identified. The top 10 are summarized in Table 2, and the full top 100 list is provided in Table S4. In brief, the three highest‐ranked papers were “Experimental gingivitis in man. II. A longitudinal clinical and bacteriological investigation” by Theilade et al. published in 1966 [4], “The role of inflammatory mediators in the pathogenesis of periodontal disease” by R.C. Page published in 1991 [19], and “The effect of mouthrinses and topical application of chlorhexidine on the development of dental plaque and gingivitis in man” by Löe and Schiøtt published in 1970 [20], with 744, 740, and 649 citations, respectively.

**TABLE 2:** Top 10 papers according to total citation count (TCC) by June 1, 2025 (for top 100 see Table S4).

Rank	TCC	Author (year)	Title
1	744	Theilade et al. (1966) [4]	Experimental gingivitis in man. II. A longitudinal clinical and bacteriological investigation.
2	740	R.C. Page (1991) [19]	The role of inflammatory mediators in the pathogenesis of periodontal disease.
3	649	Löe and Schiøtt (1970) [20]	The effect of mouthrinses and topical application of chlorhexidine on the development of dental plaque and gingivitis in man.
4	530	H. Birkedal‐Hansen (1993) [21]	Role of cytokines and inflammatory mediators in tissue destruction.
5	443	Offenbacher et al. (1986) [22]	The use of crevicular fluid prostaglandin E2 levels as a predictor of periodontal attachment loss.
6	405	Golub et al. (1983) [23]	Minocycline reduces gingival collagenolytic activity during diabetes. Preliminary observations and a proposed new mechanism of action.
7	353	Masada et al. (1990) [24]	Measurement of interleukin‐1 alpha and −1 beta in gingival crevicular fluid: implications for the pathogenesis of periodontal disease.
8	349	Kornman and Loesche (1980) [25]	The subgingival microbial flora during pregnancy.
9	346	Yaegaki and Sanada (1992) [26]	Volatile sulfur compounds in mouth air from clinically healthy subjects and patients with periodontal disease.
10	342	Dreyer et al. (2018) [27]	Epidemiology and risk factors of peri‐implantitis: A systematic review.

Moreover, according to ACY, “Epidemiology and risk factors of peri‐implantitis: A systematic review” by Dreyer et al. published in 2018 [27], “Burden of severe periodontitis and edentulism in 2021, with projections up to 2050: The Global Burden of Disease 2021 study” by Nascimento et al. published in 2024 [28], and “Hyperglycemia modulates M1/M2 macrophage polarization via reactive oxygen species overproduction in ligature‐induced periodontitis” by Zhang et al. published in 2021 [29] were the most impactful papers. Table 3 and Table S5 present the top 10 and 100 studies based on ACY, respectively.

**TABLE 3:** Top 10 papers according to average citation per year (ACY) by June 1, 2025 (for top 100 see Table S5).

Rank	ACY	TCC	Author (year)	Title
1	48.86	342	Dreyer et al. (2018) [27]	Epidemiology and risk factors of peri‐implantitis: A systematic review.
2	46.00	46	Nascimento et al. (2024) [28]	Burden of severe periodontitis and edentulism in 2021, with projections up to 2050: The Global Burden of Disease 2021 study.
3	41.00	164	Zhang et al. (2021) [29]	Hyperglycemia modulates M1/M2 macrophage polarization via reactive oxygen species overproduction in ligature‐induced periodontitis.
4	37.00	37	Isola et al. (2024) [30]	Effect of quadrantwise versus full‐mouth subgingival instrumentation on clinical and microbiological parameters in periodontitis patients: A randomized clinical trial.
5	31.75	254	Ferreira et al. (2017) [31]	Impact of periodontal disease on quality of life: a systematic review.
6	28.29	198	Noronha Oliveira et al. (2018) [32]	Can degradation products released from dental implants affect peri‐implant tissues?
7	28.00	56	Isola et al. (2023) [33]	Impact of periodontitis on gingival crevicular fluid miRNAs profiles associated with cardiovascular disease risk.
8	21.76	740	R.C. Page (1991) [19]	The role of inflammatory mediators in the pathogenesis of periodontal disease.
9	21.13	169	de Jong et al. (2017) [34]	The intricate anatomy of the periodontal ligament and its development: Lessons for periodontal regeneration.
10	19.50	78	Wong et al. (2021) [35]	Periodontal disease and quality of life: Umbrella review of systematic reviews.

### 3.6 Author Clusters and Collaborations

**FIGURE 6:**
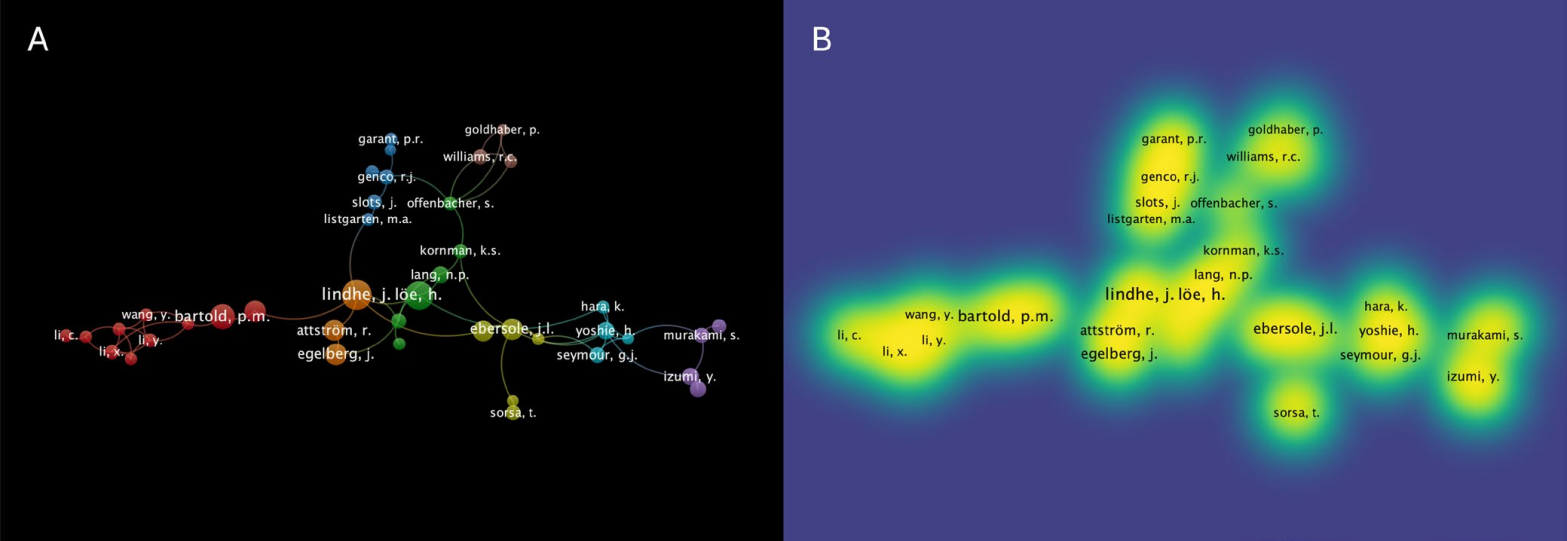
Co‐authorship analysis of authors with ≥ 20 publications presented with (A) network and (B) density visualization according to the data from 1966 to June 1, 2025.

**FIGURE 7:**
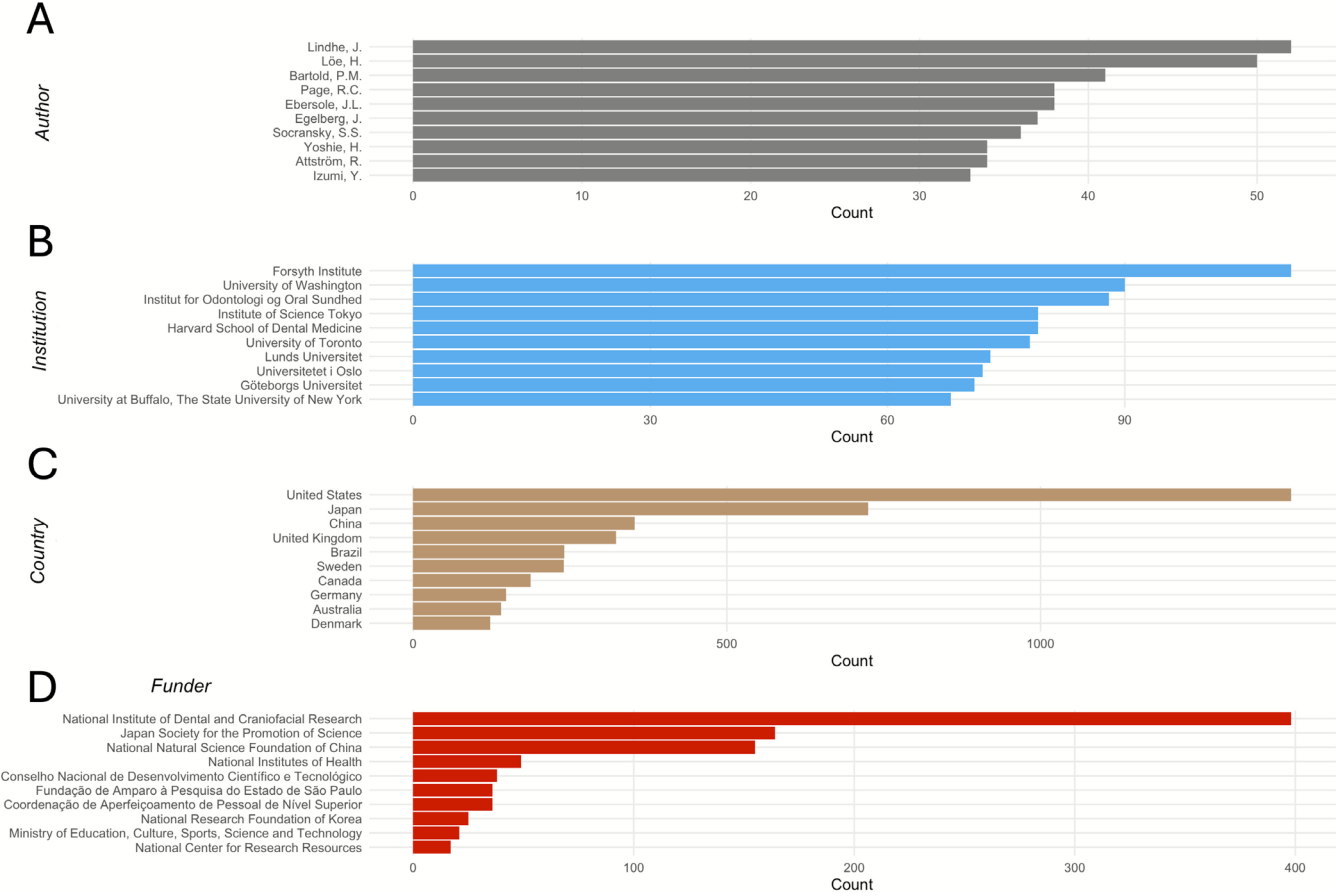
Top 10 (A) authors, (B) institutions, (C) countries, and (D) funders according to total number of publications from 1966 to June 1, 2025.

Over the 60‐year period, a total of 10 876 researchers contributed to *JPR* publications, yielding a mean of 4.55 co‐authors per article. International collaboration characterized 16.62% of papers. In parallel, 476 single‐authored articles were identified. Analysis of the author collaboration network identified 41 authors with ≥ 20 publications, forming 8 clusters (Figure 6A), and 159 authors with ≥ 10 publications, forming 17 clusters (Figure S1). The most productive contributors were J. Lindhe (52 publications), H. Löe (50 publications), P.M. Bartold (41 publications), J.L. Ebersole (38 publications), and R.C. Page (38 publications). The full list of 10 and 100 most productive authors in terms of number of publications is presented in Figure 7A and Table S6, respectively. The largest cluster—Cluster 1 (shown in red in Figure 6A)—comprised 10 authors, with P.M. Bartold as its most productive member, followed by R.C. Page. Cluster 2 (in green in Figure 6A) included 6 authors, led by H. Löe in number of publications. J. Lindhe, the journal's most prolific author by publication count, was part of Cluster 7 (in orange in Figure 6A), which contained two other members, J. Egelberg and R. Attström.

### 3.7 Country and Institution Clusters, Research Interests, and Collaborations

Regarding institutions, the Forsyth Institute (*n* = 111), the University of Washington (*n* = 90), and the Institut for Odontologi og Oral Sundhed (*n* = 88) ranked first, second, and third, respectively. Also, Harvard School of Dental Medicine (*n* = 79) and Institute of Science Tokyo (*n* = 79) tied for fourth place (Figure 7B). Overall, authors from 86 countries across six continents published in *JPR*.

**FIGURE 8:**
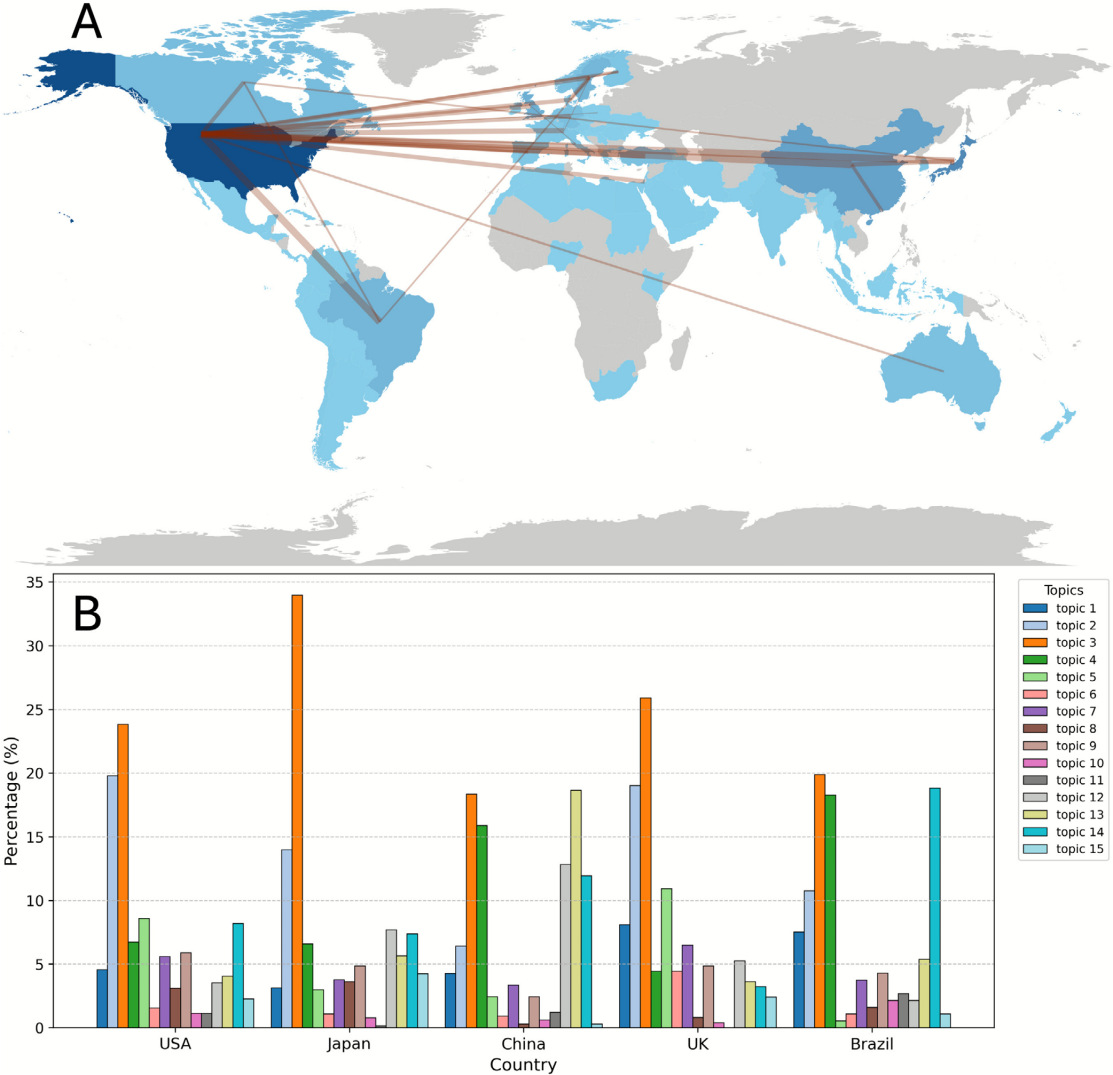
(A) International collaboration network between countries with ≥ 10 publications in *JPR* from 1966 to June 1, 2025. The width of the connecting line presents the number of publications. (B) Frequency distribution of research topics in the top five most prolific countries. (Topic ID: (1) Epidemiology, Risk, & Public Health; (2) Microbiology & Biofilm Ecology; (3) Host Response & Inflammatory Biomarkers; (4) Systemic Links & Comorbidities; (5) Diagnostics & Measurement; (6) Non‐Surgical & Resective/Access Periodontal Therapy; (7) Antimicrobial & Adjunctive Strategies; (8) Regenerative Periodontal Therapy; (9) Biomaterials & Biologics; (10) Soft & Hard Tissue Augmentation; (11) Placement & Maintenance of Dental Implants; (12) Tissue Engineering & Cell‐Based Therapies; (13) Genetics, Epigenetics, & Multi‐Omics; (14) Host‐Modulation & Pharmacologic Interventions; (15) Miscellaneous.)

As shown in Figure 7C, authors affiliated with the United States contributed the most, with 1399 publications, followed by Japan (*n* = 725), China (*n* = 353), the United Kingdom (*n* = 323), and Brazil (*n* = 241). The full list of all 86 countries and top 100 institutions according to the number of publications is available in Tables S7 and S8. Furthermore, international collaboration analysis showed that the most frequent country pairs involved the United States: Brazil–United States (*n* = 50), Japan–United States (*n* = 48), China–United States (*n* = 34), Switzerland–United States (*n* = 29), and the United Kingdom–United States and Turkey–United States (each *n* = 25). Figure 8A presents countries with ≥ 10 collaborative publications, and Table S9 and Figure S2 present countries with ≥ 5 collaborative publications across the past 60 years.

A significant association between country and research topic was revealed ($\chi^{2}$ = 414.76, df = 56, *p* < 0.001) in the chi‐square test. Standardized residual analysis showed that each country exhibited distinctive research preferences (Figure 8B). The United States showed significant emphasis on “Diagnostics & Measurement” (*r* = 3.54) and “Microbiology & Biofilm Ecology” (*r* = 3.31). Japan predominantly concentrated on “Host Response & Inflammatory Biomarkers” (*r* = 4.10). China displayed strong overrepresentation in “Genetics, Epigenetics & Multi‐Omics” (*r* = 8.53), “Tissue Engineering & Cell‐Based Therapies” (*r* = 5.04), and “Systemic Links & Comorbidities” (*r* = 4.47). The United Kingdom exhibited higher involvement in “Non‐Surgical & Resective/Access Periodontal Therapy” (*r* = 3.52), “Diagnostics & Measurement” (*r* = 3.31), and “Epidemiology, Risk, & Public Health” (*r* = 2.42). Brazil, meanwhile, stood out for its focus on “Host‐Modulation & Pharmacologic Interventions” (*r* = 4.60) and “Systemic Links & Comorbidities” (*r* = 4.47). Table S10 present the complete list of over‐ and under‐represented topics in each country. In 2025 (up to October 31), China has been the most prolific submitting country, contributing 27.48% of all submissions, followed by India (9.58%), Brazil (8.32%), Türkiye (6.84%), and the United States (5.02%). Notably, China has consistently been the leading submitting country over the past 5 years, and the composition of the top five most prolific submitting countries has remained unchanged during this period.

### 3.8 Funding

Funding sources were acknowledged in 1573 publications, while 3107 documents had no funding acknowledgments. An assessment of funding acknowledgments in *JPR* articles identified support from 160 different funding agencies overall, with 15 providing backing to at least 10 papers. The leading funder was the National Institute of Dental and Craniofacial Research, acknowledged in 398 articles, followed by the Japan Society for the Promotion of Science (164) and the National Natural Science Foundation of China (155). The top 10 funding institutions are summarized in Figure 7D, and the top 100 funders are listed in Table S11.

### 3.9 Altmetrics Analysis

**FIGURE 9:**
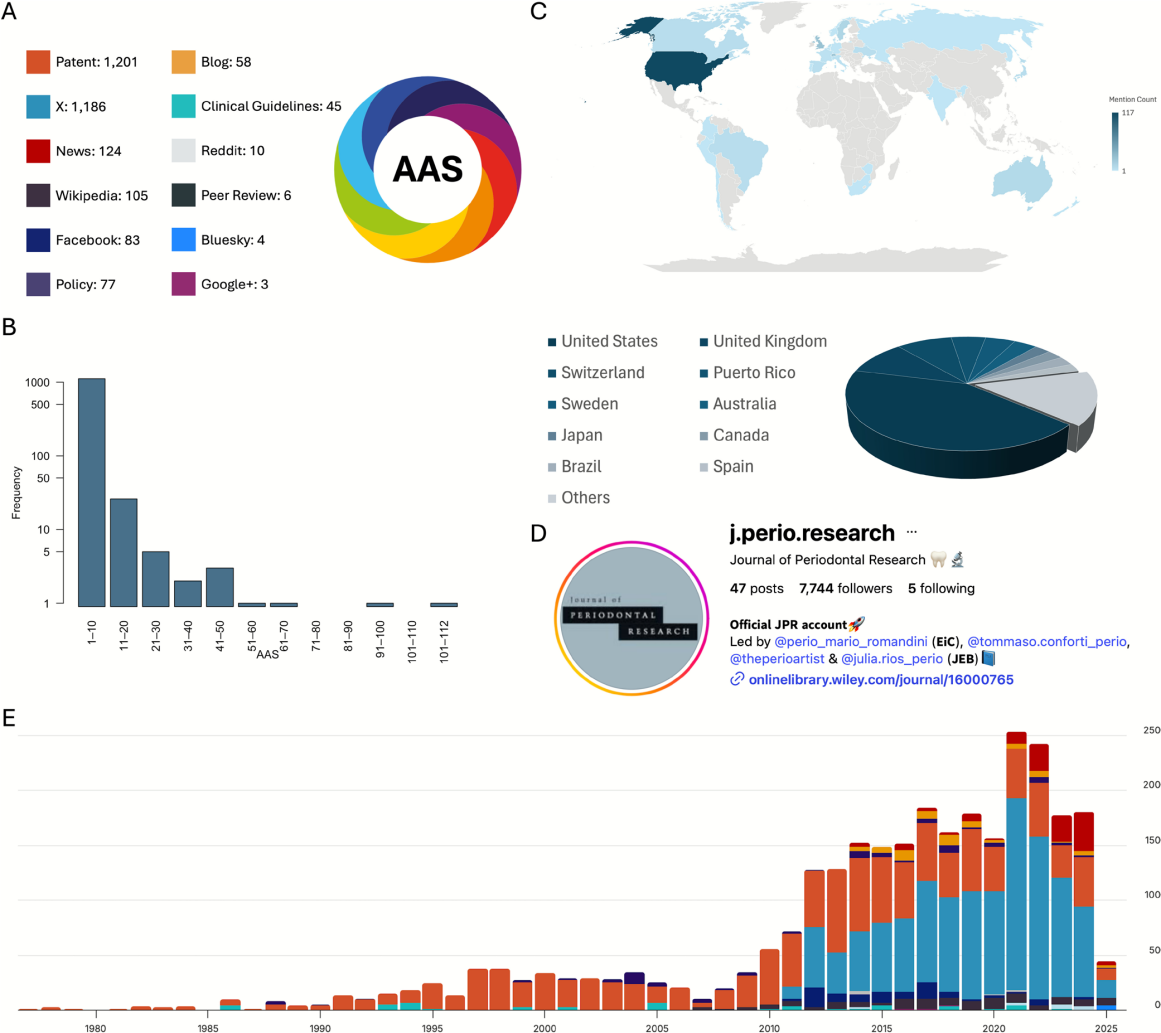
Altmetrics (until June 1, 2025) analysis visualization. (A) Total number of mentions by source, with colors corresponding to Altmetric source categories; (B) Frequency distribution of the Altmetrics Attention Across (AAS); (C) Geographical distribution of AAS by country, aggregated across sources; (D) Instagram page view of the journal's official account; (E) Chronological distribution of mentions from 1977 to June 1, 2025.

Over the past 60 years, 1150 of the articles published in *JPR* have collectively received a total of 2902 mentions according to Altmetric data, with individual studies showing an AAS score ranging from 1 to 112 [36]. The earliest Altmetric mention of a paper published in *JPR* dates back to 1977, when Gjermo et al.'s study on plaque inhibition by different bis‐biguanides was cited in a patent [37]. Since then, the journal has been highlighted in various platforms and sources, with more than 80% of the mentions sourcing from either patents or X, each having more than 1000 mentions (Figure 9A). Following patents (41.38%) and X (40.87%), which far exceeded all others, news (4.27%), Wikipedia (3.62%), and Facebook (2.86%) were the next most common sources of attention.

As shown in Figure 9B, among the 1150 studies with reported mentions, over 1000 received only a single mention, whereas just four studies had more than 50 mentions [26, 36, 38, 39], with one exceeding 100 [36]. From a chronological perspective (Figure 9E), most mentions before 2010 came from patents. After 2010, however, with the growing popularity of social networks, X became the leading source of mentions to the journal. The top three ranked articles based on their AAS were: “Antibacterial effects of blackberry extract target periodontopathogens (2013)” (AAS = 112) [36], “Use of floss/interdental brushes is associated with lower risk for new cardiovascular events among patients with coronary heart disease (2015)” (AAS = 91) [38], and “Volatile sulfur compounds in mouth air from clinically healthy subjects and patients with periodontal disease (1992)” (AAS = 67) [26]. The full ranking of top 10 and 100 papers according to AAS is presented in Table 4 and Table S12, respectively. Also, the top three papers according to AAASY were “Phytocannabinoids and gingival inflammation: Preclinical findings and a placebo‐controlled double‐blind randomized clinical trial with cannabidiol (2024)” (AAS = 28; AAASY = 28) [44], “Influence of gender on periodontal outcomes: A retrospective analysis of eight randomized clinical trials (2024)” (AAS = 27; AAASY = 27) [45], and “Demystifying the connection between periodontal disease and chronic kidney disease–An umbrella review (2023)” (AAS = 43; AAASY = 21.50) [41], respectively. Table 5 present the top 10 articles according to AAASY. As shown in Figure 9C, over 40% of the mentions originated from the United States, followed by the United Kingdom and Switzerland as the next most frequent sources. Figure S3 shows the geographical distribution of mentions, categorized by their Altmetric source.

**TABLE 4:** Top 10 papers according to the Altmetric Attention Score (AAS) by June 1, 2025 (for top 100 see Table S12).

Rank	AAS	Author, year	Title
1	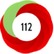	González et al. (2013) [36]	Antibacterial effects of blackberry extract target periodontopathogens.
2	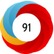	Reichert et al. (2015) [38]	Use of floss/interdental brushes is associated with lower risk for new cardiovascular events among patients with coronary heart disease.
3	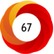	Yaegaki and Sanada (1992) [26]	Volatile sulfur compounds in mouth air from clinically healthy subjects and patients with periodontal disease.
4	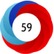	Laleman et al. (2018) [39]	Influence of tongue brushing and scraping on the oral microflora of periodontitis patients.
5	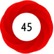	Kornman and Loesche (1980) [25]	The subgingival microbial flora during pregnancy.
6	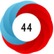	Maruyama et al. (2022) [40]	Association between serum miRNAs and gingival gene expression in an obese rat model.
7	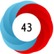	He et al. (2023) [41]	Demystifying the connection between periodontal disease and chronic kidney disease—An umbrella review.
8	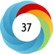	Long et al. (2017) [42]	Association of oral microbiome with type 2 diabetes risk.
9	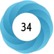	Schiött and Löe (1970) [43]	The origin and variation in number of leukocytes in the human saliva.
10	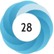	Jirasek et al. (2024) [44]	Phytocannabinoids and gingival inflammation: Preclinical findings and a placebo‐controlled double‐blind randomized clinical trial with cannabidiol.

**TABLE 5:** Top 10 papers according to the average Altmetric Attention Score (AAS) per year (AAASY) by June 1, 2025.

Rank	AAASY	AAS	Author (year)	Title
1	28.00	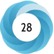	Jirasek et al. (2024) [44]	Phytocannabinoids and gingival inflammation: Preclinical findings and a placebo‐controlled double‐blind randomized clinical trial with cannabidiol.
2	27.00	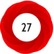	Castro Dos Santos et al. (2024) [45]	Influence of gender on periodontal outcomes: A retrospective analysis of eight randomized clinical trials.
3	21.50	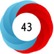	He et al. (2023) [41]	Demystifying the connection between periodontal disease and chronic kidney disease–An umbrella review.
4	16	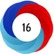	Nascimento et al. (2024) [28]	Burden of severe periodontitis and edentulism in 2021, with projections up to 2050: The Global Burden of Disease 2021 study.
5	14.67	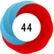	Maruyama et al. (2022) [40]	Association between serum miRNAs and gingival gene expression in an obese rat model.
6	8.62	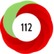	González et al. (2013) [36]	Antibacterial effects of blackberry extract target periodontopathogens.
7	8.27	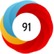	Reichert et al. (2015) [38]	Use of floss/interdental brushes is associated with lower risk for new cardiovascular events among patients with coronary heart disease.
8	7.38	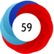	Laleman et al. (2018) [39]	Influence of tongue brushing and scraping on the oral microflora of periodontitis patients.
9 (tie)	7	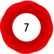	Oh et al. (2022) [46]	Transepithelial channels for leukocytes in the junctional epithelium.
9 (tie)	7	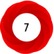	J. Caton (1992) [47]	Biological and measurement issues critical to design of gingivitis trials.
9 (tie)	7	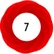	Hong et al. (2016) [48]	Anti‐inflammatory and anti‐osteoclastogenic effects of zinc finger protein A20 overexpression in human periodontal ligament cells.
9 (tie)	7	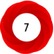	Shen et al. (2024) [49]	Abnormal amyloid precursor protein processing in periodontal tissue in a murine model of periodontitis induced by *Porphyromonas gingivalis* .

**FIGURE 10:**
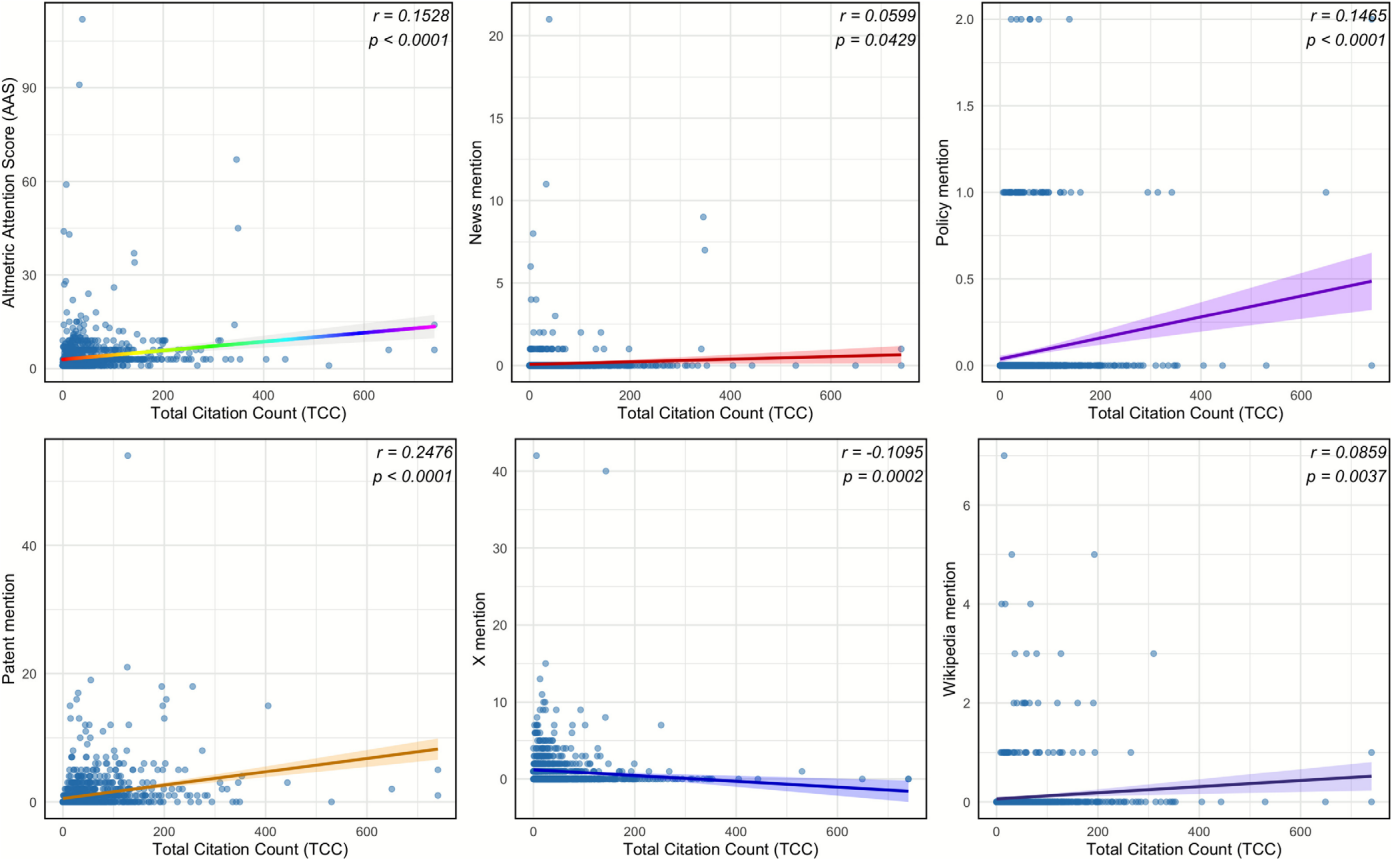
Linear correlation analysis between total citation count (TCC) and (top left) Altmetric Attention Score (AAS), (top middle) news mentions, (top right) policy mentions, (bottom left) patent mentions, (bottom middle) X (Twitter) mentions, and (bottom right) Wikipedia mentions. Each plot displays the correlation coefficient (*r*) and *p*‐value.

The results of the correlation analysis demonstrated that TCC was significantly correlated with AAS, as well as mention counts from news, policies, patents, X, and Wikipedia (Figure 10). Except for X mentions, which exhibited a weak negative correlation with TCC (*r* = −0.1095, *p* = 0.0002), all other mention counts and AAS demonstrated significant positive correlations with TCC. The strongest correlation, yet still classified as weak, was observed between TCC and patent mentions (*r* = 0.2476, *p* < 0.0001), followed by TCC and AAS (*r* = 0.1528, *p* < 0.0001) and TCC and policy mentions (*r* = 0.1465, *p* < 0.0001), both of which were categorized as weak correlations. The correlations between TCC and Wikipedia mentions (*r* = 0.0859, *p* = 0.0037) and TCC and news mentions (*r* = 0.0599, *p* = 0.0429) were significant but very weak.

## 4 Discussion

This study provided a 60‐year overview of *JPR*, combining conventional bibliometric indices with AI‐assisted analyses and Altmetrics to map the journal's scope, focus, performance, and societal impact. Overall, *JPR* has mainly concentrated on “Host‐Response & Inflammatory Biomarkers” and “Microbiology & Biofilm Ecology”, with most contributions involving in vitro, ex vivo, animal, or other preclinical designs, reflecting the field's mechanistic foundation. Research output has grown steadily, with a CAGR of 2.62%, reaching 4680 publications authored by more than 10 000 researchers across 86 countries, indicating sustained growth and broad international reach. Furthermore, the journal's citation metrics continue to trend upward, with a projected 2025 JIF (to be released in June 2026) of around 5.1—which will, for the first time, include approximately 40% of the articles published under the new leadership. In line with this trajectory, *JPR* articles published online during 2025 have so far, until October 31, 2025, accrued a mean of 1.54 citations per citable item within the same year, exceeding the corresponding rate reported for other well‐recognized journals in the field, such as the *Journal of Clinical Periodontology* (0.76). This suggests that the JIF is likely to continue growing substantially in the coming years.

Over the past six decades, the thematic evolution of *JPR* has closely mirrored the broader paradigm shifts that have shaped periodontology as a scientific discipline. In its formative years, the journal centered on elucidating the etiopathogenesis of periodontitis as an infection‐driven disease, with research largely devoted to microbial ecology and host‐response mechanisms [4, 50]. As the field advanced toward a host‐modulated understanding of disease, *JPR* reflected this transition by emphasizing studies on inflammation, immune regulation, and pharmacologic strategies aimed at modifying the host response. In recent years, periodontology has entered a new phase grounded in systems‐level biology and personalized medicine. No longer viewed in isolation, the periodontium is increasingly recognized as part of an integrated network connecting oral and systemic health [51, 52, 53]. Correspondingly, *JPR* has expanded its focus to encompass research on systemic associations and comorbidities, genetic and multi‐omics profiling, and precision diagnostics and individualized interventions supported by emerging technologies such as AI. Importantly, this influence has not been unidirectional. While the field's changing paradigms have influenced the journal's research priorities, the pioneering studies published in *JPR* have, in turn, shaped the trajectory of periodontology itself. Through disseminating foundational discoveries and emerging concepts, *JPR* has actively contributed to defining, refining, and advancing the discipline's scientific identity.

One of the important noted patterns was the extensiveness of international participation. Authors from 86 countries across six continents contributed to *JPR*, and the international co‐authorship network encompassed 68 pairs of countries. This wide involvement underscores the journal's commitment to global collaboration, diversity, and inclusion, and its ability to attract researchers with different backgrounds, research interests, and expertise. As shown, different countries have emphasized different research themes, resulting in publications spanning a broad range of topics. Notably, China and Brazil have shown strong contributions in areas such as “Systemic Links & Comorbidities” and “Genetics, Epigenetics, & Multi‐Omics”. Given the journal's and the broader discipline's current trajectory toward systems biology and other innovative, technology‐driven approaches, it is reasonable to anticipate an even greater representation of these countries in future publications. This commitment is also evident in editorial policy. Rather than relying on informal co‐optation, *JPR* issued an open international call for Associate Editors and Editorial Board members, which produced a diverse team spanning 25 countries on six continents [54]. As the editor‐in‐chief has emphasized, the new composition reflects both quality and diversity. In practical terms, submissions are handled by domain experts across periodontology and implant science, while the board demonstrates geographic inclusivity, gender balance, and representation across career stages, achieved through merit‐based selection from a broad applicant pool.

One reflection of this diverse and robust editorial structure is the journal's expedited peer‐review process: the time to first decision and acceptance has been reduced to roughly one‐sixth and one‐half, respectively, of what it was 2 years ago. This prompt review process, combined with the board's continued commitment to quality, constructive spirit, and scientific rigor, has contributed to the substantial increase in submissions and full‐text reads in *JPR*. The geographical patterns observed in this study also align well with prior bibliometric reports in the field. Across the bibliometric analyses conducted in the discipline, the United States has consistently been reported as the most prolific country in terms of total number of publications [8, 9, 55]. However, in recent years, dental and periodontal research has seen a substantial increase in contributions from China, Brazil, and India, all of which are now among the top submitting countries to *JPR*. This trend may eventually lead to a demographic, as well as thematic, shift in the journal's bibliometric profile and, more broadly, in the field as a whole.

In the 21st century, the rapid growth of social and web platforms has changed how research circulates and is discussed, including within scientific communities. In parallel, alternative metrics (“Altmetrics”) have been introduced to capture forms of impact that traditional indicators may miss, such as attention in news media, policy documents, clinical guidelines, blogs, and social media. Relying solely on classic journal performance metrics, which typically involve publication counts, citation counts, or citation‐derived indices, can underrepresent a journal's reach beyond academia. Altmetric indicators do not replace citation‐based measures, but they complement them by more granular signals of engagement and by reflecting interest among audiences outside the scholarly literature (e.g., clinicians, patients, journalists, and policymakers) [56]. Altmetric indices also have limitations that warrant cautious interpretation. Online attention is not always directly associated with methodological quality, as items can trend for reasons extraneous to rigor, such as novelty, controversy, and press releases [57]. General social platforms (e.g., X/Twitter, Facebook) allow amplification by non‐experts and automated accounts, and user identity is often opaque, complicating provenance and accountability. Engagement captured by Altmetrics is typically lightweight and short‐lived (likes, shares, retweets), whereas citations usually follow close reading, critical appraisal, and integration into a scholarly argument [58]. Altmetric indicators are also more susceptible to manipulation and cannot, on their own, distinguish endorsement from criticism [59]. Accordingly, they should be treated as complementary, context‐dependent signals rather than surrogates for scientific influence [60].

For these reasons, both citation‐based metrics and Altmetric data were evaluated in the present study to provide a more complete picture of the journal's scholarly and societal influence. However, it should be noted that even both citation‐based and societal‐mention‐based metrics may not always reflect methodological integrity and rigor, and that quantifying or assessing methodological rigor independently is challenging, as its primary evaluation method remains meticulous expert review—an approach that becomes nearly infeasible when the number of publications is large, as in the present study. Upon comparison of Altmetric and citation patterns, it was noted that two papers ranking in the top 10 by AAS were also ranked in the top 10 by TCC. Topics with broad public salience were addressed in these articles, one examining oral malodor in relation to periodontitis and tongue coating [26], and another exploring pregnancy‐associated changes in the subgingival microbiota [25], This co‐occurrence of considerable scholarly and public impact is indicative of an influence that extends beyond the scholarly record, while rigorous standards of scientific contribution are maintained, demonstrating the journal's multifaceted scope across academic and societal domains. Public awareness and knowledge appear to have been particularly affected together when clinically tangible health issues were investigated. The highest AAS (112) was recorded for “Antibacterial effects of blackberry extract target periodontopathogens” [36], a topic that tends to be amplified and mentioned by news and social media due to its inherent captivation to the general population. However, in the correlation analysis, it was noted that the strongest, yet still weak, correlation existed between TCC and patent mentions, which are typically more related to scientific rather than social media attention, while the only significant negative correlation was observed between TCC and X mentions. This difference, not only in magnitude but also in direction, suggests that scholarly contribution is not always parallel with societal impact. For instance, many highly cited articles are mechanistic studies addressing molecular‐ or cellular‐level questions that may not attract the interest of the general public. In comparative terms, public attention to *JPR* appears to have begun earlier than for other old and prestigious journals in periodontics. For instance, despite the *Journal of Periodontology* being founded 35 years earlier, its first Altmetric mention, which is by Centers for Disease Control and Prevention (CDC) to “Production and origin of oral malodor: a review of mechanisms and methods of analysis” by J. Tonzetich [61], was recorded 1 year after *JPR*'s first appearance in a patent [37]. This earlier emergence of Altmetric attention for *JPR* may be attributed to its past stronger orientation toward basic and translational research, which often bridges laboratory findings with potential clinical and industrial applications, thereby increasing the likelihood of early citation in societal and nonacademic sources, particularly patents that emerged prior to the advent of internet‐based social media platforms such as X and Facebook.

The modification made to the Oxford 2011 LEV classification in the present study is consistent with previous bibliometric analyses of the field [8]. This approach was followed to allow comparison with prior reviews and to give readers a familiar benchmark. However, this classification fails to reflect the true LEV of some of the studies since it is mainly designed to present the LEV of interventional studies. In practice, it privileges randomized clinical trials (RCTs) and clinical cohorts, while basic, translational, and discovery work are placed at lower levels by default, regardless of their internal validity and focus question. For instance, in many cases where the research question is trying to answer a mechanistic question, animal studies hold the highest LEV. Such designs can provide direct causal evidence at the tissue, cellular, or molecular level, which is not the aim of clinical interventions. Therefore, considering the main focus of *JPR* on mechanistic research, this classification might underestimate LEV.

ACY and AAASY were calculated in the present study to weight TCC and AAS by publication date, respectively. However, even these weighted metrics underestimate the true citation or societal impact of recently published studies. First, all articles published within the same calendar year were assigned the same age, regardless of their month of publication, which is not indicated in Web of Science exportable data. While this slight discrepancy may not affect older publications, a study published in January 2024—aged 17 months at the time of the present study's search—and another published in December 2024—aged only 6 months—were both assigned an age of 1 year. Second, given the peer‐review and publication process, it often takes time for an article's impact to be reflected as citations in subsequently published works. This temporal bias also extends to Altmetric analyses, since many recently published studies may not attract media attention in the short term.

Despite clear advantages that fit the objectives of this study, such as enabling export of document‐level YCC, Web of Science has limitations that might affect the findings. First, compared with other major indexing platforms (e.g., Scopus), Web of Science has narrower journal coverage. While this does not affect the pool of studies published in *JPR* since all exported records were cross‐checked against the journal's official website, it may underestimate TCC at both the journal and document levels. Another limitation was the absence of YCC prior to 1972 in Web of Science. Moreover, similar to most other major databases, indexing in Web of Science often takes place some time after early online publication. This delay can lead to a marginal underestimation in both publication counts and citation indices. Furthermore, because at the time the dataset for the present study was compiled only 17 months had passed since the new leadership began—and many articles published in 2024 had been inherited from the previous leadership—only a limited number of articles accepted under the new team were included. This prevented a direct comparison of past journal trends with current ones under the new leadership, making it difficult to quantify the impact of recent changes on the journal's output and performance. Additionally, because the Altmetric database does not track Instagram mentions, the impact of the journal's new official Instagram page (@j.perio.research)–which currently has more than 7800 followers and over 281 000 monthly views in October 2025 (Figure 9D)–could not be captured in the Altmetric analysis.

Finally, while the proposed use of LLMs for topic and LEV categorization demonstrated acceptable performance and holds promise for future applications in the field, certain inherent biases and limitations of this emerging technology should be acknowledged when applying it to bibliometrics. Since most training data for LLMs are in English, these models tend to perform less effectively on non‐English texts or writing styles that are underrepresented in the training corpus [62], limiting the applicability of this approach to non‐English literature. Moreover, it has been reported that the factual accuracy of LLMs can gradually decline over time, especially in long interactions, a phenomenon referred to as “semantic drift” [63]. In addition, the generation of content that appears plausible but is actually false, fabricated, or unsupported by real data, commonly known as “hallucination”, is a recognized issue in LLMs [64]. For instance, in the context of the present study, because only titles, abstracts, and author keywords were used in the categorization process, the model might incorrectly classify a study with an ambiguous abstract or telegraphic title as an RCT, even when no evidence of randomization is explicitly stated. Furthermore, as probabilistic neural networks, LLMs are inherently sensitive to small variations in input phrasing or prompt formatting, which can alter their outputs. Although a deterministic prompting setup (temperature = 0 and fixed model snapshot) was employed to ensure reproducibility, such configurations cannot fully eliminate potential variability across model updates. Finally, LLMs rely on pattern recognition rather than true domain understanding or causal reasoning, which may affect their accuracy in borderline or methodologically complex cases. To mitigate all these risks, constrained codebooks were applied, prompts were kept stable, records were processed in fixed‐size batches, and performance was validated against an expert‐labeled pilot set. Nevertheless, residual classification errors and biases may persist and should be considered when interpreting topic and LEV assignments.

On the other hand, this study presented the complete 60‐year history of the journal, whereas several bibliometric studies marking other journals' milestones have focused only on a subset of highly cited publications or a limited recent timespan [55]. This approach provided a general picture of the journal while also identifying its most impactful articles. An AI‐assisted approach was also employed for the first time in the bibliometric analysis of periodontal, and even more broadly, dental literature. To the author's knowledge, only one report has used AI for bibliometric analysis in the medical literature [12]. In that study, model performance was described qualitatively, and the authors indicated that GPT‐4 produced fewer than two misclassifications per 11 records, without specifying the exact number of records screened or the LLM configuration. Moreover, the most recent version of GPT (GPT‐5‐mini) was employed in the present study and, as shown, achieved significantly higher agreement with expert classification compared with older GPT models. The results of this study indicated that AI, more particularly LLM models, can serve as a useful yet accurate tool in bibliometric studies. Specifically, AI‐assisted bibliometrics can reshape evidence synthesis in dental research by enabling large‐scale analysis of extensive literature corpora that would otherwise be impractical for manual review. The most time‐consuming step in bibliometric studies typically involves expert labeling of articles by topic, study design, level of evidence, or other variables; however, the use of AI can perform these tasks in a controlled manner, with substantial time efficiency and acceptable precision. As mentioned earlier, most previous bibliometric studies have relied on cross‐sectional selections, such as the most cited or most recent, whereas AI‐assisted approaches enable longitudinal analysis of the literature by including more publications, making it easier to detect temporal trajectories and visualize their evolution over time. This scalability makes it possible to trace how evidence and research focus have evolved, offering valuable context for future investigations.

The bibliometric and Altmetric findings of the present study also provide insights that may inform *JPR*'s editorial direction in the coming decade. The journal's growing representation in emerging domains such as multi‐omics, tissue engineering, and AI‐assisted diagnostics reflects an ongoing shift toward interdisciplinarity and data integration, offering an opportunity to further connect biological, clinical, and computational research. The extensive international authorship spanning 86 countries underscores *JPR*'s global reach and its role as an inclusive platform for collaboration across diverse scientific communities. At the same time, the strong Altmetric attention observed across patents, social media platforms, and news outlets highlights the importance of enhancing public visibility and engagement. In this context, advancing open science practices, promoting data availability through open‐access publication, and maintaining an active presence on social media channels may collectively strengthen transparency, accessibility, and worldwide dissemination of research published in *JPR* while continuing to foster high‐impact and interdisciplinary scholarship in periodontology.

## 5 Conclusion

Throughout its 60‐year history, *JPR* has contributed significantly to the periodontal literature, mostly with publications in “Host‐Response & Inflammatory Biomarkers” and “Microbiology & Biofilm Ecology” and studies employing a pre‐clinical design. The journal has also demonstrated significant public and societal impact, mainly across patents and X platform. Furthermore, the journal demonstrates sustained growth alongside a commitment to rigorous and inclusive research.

Because only a limited number of articles published under the new leadership were included in this analysis, it was not possible to directly quantify the journal's current output and performance. Nonetheless, clear trends have already emerged: the number of submissions and full‐text views has almost doubled compared to 2023, while first‐decision and acceptance times have been reduced to one sixth and one half of their 2023 values, respectively. These developments likely underpin projections indicating that the JIF is poised for substantial growth in the coming years.

Finally, the AI model used in this study showed good to excellent agreement with a human field expert for labeling topics and LOE. This study represents one of the earliest applications of AI‐assisted classification in bibliometric research within medicine, and, to the authors' best knowledge, the first in dentistry, highlighting its potential to support and facilitate future evidence mapping and large‐scale literature synthesis.

## Author Contributions

**Parham Hazrati:** conceptualization, data curation, formal analysis, investigation, methodology, visualization, writing – original draft, writing – review and editing. **Tara Zibandehkhooy:** conceptualization, data curation, formal analysis, investigation, methodology, visualization, writing – original draft, writing – review and editing. **Hamoun Sabri:** conceptualization, data curation, formal analysis, investigation, methodology, visualization, writing – original draft, writing – review and editing. **Mario Romandini:** conceptualization, writing – original draft, writing – review and editing. **Shayan Barootchi:** conceptualization, methodology, project administration, supervision, writing – original draft, writing – review and editing.

## Funding

The authors have nothing to report.

## Ethics Statement

This study was exempt from institutional review board approval and ethical review, as it relied solely on publicly available data and information.

## Conflicts of Interest

Prof. Mario Romandini is the Editor‐in‐Chief of *JPR*. Also, Dr. Shayan Barootchi is an Associate Editor of *JPR*, and Dr. Hamoun Sabri is a member of the Junior Editorial Board. In accordance with Wiley's standard policies for submissions by Editors and Editorial Board members, they were excluded from the editorial decision‐making related to this article and remained blinded throughout the peer‐review process.

## Supporting information


**Data S1:** jre70071‐sup‐0002‐DataS1.zip.

**Data S2:** jre7001‐sup‐0002‐DataS2.docx.

## Data Availability

The data that support the findings of this study are available from the corresponding author upon reasonable request.
