# Supplementary material for: Mapping 60 Years of Discovery: An AI‐Driven Bibliometric and Altmetric Analysis of the Journal of Periodontal Research
Source: J Periodontal Res. 2025 Dec 30;60(12):1181–200. doi: 10.1111/jre.70071 (PMC12881886; doi:10.1111/jre.70071)
Supplement: Supplementary file 1 — Data S1: jre70071‐sup‐0002‐DataS1.zip. [file JRE-60-1181-s002.zip › jre70071-sup-0001-supinfo.docx]

**Supplementary Materials**

**Study title:** Mapping 60 Years of Discovery: An AI-Driven Bibliometric and Altmetric Analysis of the *Journal of Periodontal Research*

Table of Contents

[**SUPPLEMENTARY TABLE 1** Peer review statistic of the Journal of Periodontal Research. 2025 statistic reflect the data until Oct 30, 2025. 2](#_Toc214646701)

[**SUPPLEMENTARY TABLE 2** Absolute and relative frequencies (counts and percentages) of studies by 15 topics and six levels of evidence. 3](#_Toc214646702)

[**SUPPLEMENTARY TABLE 3** Yearly citation count (YCC) distribution across topics from 1972 to June 1, 2025. 4](#_Toc214646703)

[**SUPPLEMENTARY TABLE 4** Top 100 publications according to total citation count (TCC). 6](#_Toc214646704)

[**SUPPLEMENTARY TABLE 5** Top 100 publications according to average citation per year (ACY). 10](#_Toc214646705)

[**SUPPLEMENTARY TABLE 6** Top 100 most prolific authors according to the number of publications. 15](#_Toc214646706)

[**SUPPLEMENTARY TABLE 7** List of all 86 countries contributing to JPR, sorted according to the number of publications. 17](#_Toc214646707)

[**SUPPLEMENTARY TABLE 8** Top 100 most prolific institutions according to the number of publications. 19](#_Toc214646708)

[**SUPPLEMENTARY TABLE 9** International collaboration pairs with ≥5 coauthored publications. 21](#_Toc214646709)

[**SUPPLEMENTARY TABLE 10** Under- and over-represented research topics in each of the five most prolific countries according to the analysis of residuals (r). 23](#_Toc214646710)

[**SUPPLEMENTARY TABLE 11** Top 100 most prolific funding agencies according to number of publications. 24](#_Toc214646711)

[**SUPPLEMENTARY TABLE 12** Top 100 publications according to AAS. 26](#_Toc214646712)

[**SUPPLEMENTARY FIGURE 1** Co-authorship analysis of authors with ≥10 publications presented with **(A)** network and **(B)** density visualization. 32](#_Toc214646713)

[**SUPPLEMENTARY FIGURE 2** International collaboration network between countries with ≥ 5 publications in JPR from 1966 to 2025. The width of the connecting line presents the number of publications. 33](#_Toc214646714)

[**SUPPLEMENTARY FIGURE 3** Geographical distribution of Altmetric mentions in **(A)** X, **(B)** Facebook, and **(C)** news. 34](#_Toc214646715)

[**REFERENCES** 35](#_Toc214646716)

# **SUPPLEMENTARY TABLE 1** Peer review statistic of the *Journal of Periodontal Research.* 2025 statistic reflect the data until Oct 31, 2025.

|  | ***2021*** | ***2022*** | ***2023*** | ***2024*** | ***2025*** |
| --- | --- | --- | --- | --- | --- |
| Review invitations sent | 1,244 | 1,513 | 1,481 | 1,766 | 1,403 |
| Reviews completed | 659 | 710 | 684 | 1,071 | 1,139 |
| Median days to review completion | 13 | 15 | 14 | 8 | 8 |

# **SUPPLEMENTARY TABLE 2** Absolute and relative frequencies (counts and percentages) of studies published in *JPR* by June 1, 2025 by 15 topics and six levels of evidence.

| ***Topic*** | ***N (%) of total*** | ***Level I*** | ***Level II*** | ***Level III*** | ***Level IV*** | ***Level V*** | ***Level VI*** |
| --- | --- | --- | --- | --- | --- | --- | --- |
| Epidemiology, Risk & Public Health | 244 (5.21) | 2 (0.82) | 47 (19.26) | 109 (44.67) | 35 (14.34) | 33 (13.52) | 18 (7.38) |
| Microbiology & Biofilm Ecology | 728 (15.56) | 0 (0.0) | 73 (10.03) | 92 (12.64) | 65 (8.93) | 33 (4.53) | 465 (63.87) |
| Host Response & Inflammatory Biomarkers | 1235 (26.39) | 0 (0.0) | 81 (6.56) | 169 (13.68) | 91 (7.37) | 28 (2.27) | 866 (70.12) |
| Systemic Links & Comorbidities | 357 (7.63) | 4 (1.12) | 53 (14.85) | 123 (34.45) | 15 (4.2) | 18 (5.04) | 144 (40.34) |
| Diagnostics & Measurement | 339 (7.24) | 0 (0.0) | 73 (21.53) | 50 (14.75) | 51 (15.04) | 52 (15.34) | 113 (33.33) |
| Non-Surgical & Resective/Access Periodontal therapy | 83 (1.77) | 2 (2.41) | 43 (51.81) | 3 (3.61) | 14 (16.87) | 4 (4.82) | 17 (20.48) |
| Antimicrobial & Adjunctive Strategies | 208 (4.44) | 12 (5.77) | 72 (34.62) | 0 (0.0) | 9 (4.33) | 17 (8.17) | 98 (47.12) |
| Regenerative Periodontal Therapy | 127 (2.71) | 5 (3.94) | 20 (15.75) | 3 (2.36) | 6 (4.72) | 2 (1.57) | 91 (71.65) |
| Biomaterials & Biologics | 236 (5.04) | 0 (0.0) | 5 (2.12) | 0 (0.0) | 1 (0.42) | 6 (2.54) | 224 (94.92) |
| Soft & Hard Tissue Augmentation | 54 (1.15) | 3 (5.56) | 18 (33.33) | 4 (7.41) | 3 (5.56) | 5 (9.26) | 21 (38.89) |
| Placement & Maintenance of Dental Implants | 38 (0.81) | 1 (2.63) | 12 (31.58) | 7 (18.42) | 1 (2.63) | 5 (13.16) | 12 (31.58) |
| Tissue Engineering & Cell-Based Therapies | 258 (5.51) | 0 (0.0) | 3 (1.16) | 0 (0.0) | 2 (0.78) | 12 (4.65) | 241 (93.41) |
| Genetics, Epigenetics & Multi-Omics | 221 (4.72) | 0 (0.0) | 10 (4.52) | 79 (35.75) | 9 (4.07) | 24 (10.86) | 99 (44.8) |
| Host-Modulation & Pharmacologic Interventions | 355 (7.59) | 10 (2.82) | 16 (4.51) | 3 (0.85) | 3 (0.85) | 6 (1.69) | 317 (89.3) |
| Miscellaneous / Unclassifiable | 197 (4.21) | 0 (0.0) | 4 (2.03) | 4 (2.03) | 4 (2.03) | 58 (29.44) | 127 (64.47) |
| Total | 4680 (100.0) | 39 (0.83) | 530 (11.32) | 646 (13.8) | 309 (6.6) | 303 (6.47) | 2853 (60.96) |

# **SUPPLEMENTARY TABLE 3** Yearly citation count (YCC) distribution across topics from 1972 to June 1, 2025.

|  | ***Topics*** | | | | | | | | | | | | | | |
| --- | --- | --- | --- | --- | --- | --- | --- | --- | --- | --- | --- | --- | --- | --- | --- |
| ***Year*** | ***1*** | ***2*** | ***3*** | ***4*** | ***5*** | ***6*** | ***7*** | ***8*** | ***9*** | ***10*** | ***11*** | ***12*** | ***13*** | ***14*** | ***15*** |
| 1972 | 21 | 44 | 74 | 12 | 13 | 11 | 57 | 1 | 12 | 7 | 0 | 1 | 1 | 0 | 20 |
| 1973 | 29 | 65 | 125 | 6 | 21 | 2 | 90 | 2 | 22 | 8 | 0 | 5 | 0 | 3 | 27 |
| 1974 | 34 | 67 | 160 | 10 | 26 | 8 | 57 | 1 | 10 | 10 | 0 | 2 | 5 | 3 | 36 |
| 1975 | 25 | 104 | 147 | 5 | 33 | 5 | 71 | 4 | 14 | 7 | 0 | 10 | 2 | 6 | 30 |
| 1976 | 28 | 103 | 195 | 5 | 26 | 10 | 65 | 7 | 16 | 14 | 0 | 5 | 2 | 2 | 37 |
| 1977 | 27 | 113 | 215 | 16 | 57 | 12 | 78 | 7 | 17 | 13 | 0 | 14 | 4 | 10 | 37 |
| 1978 | 25 | 198 | 175 | 17 | 52 | 3 | 68 | 11 | 17 | 16 | 0 | 11 | 1 | 8 | 36 |
| 1979 | 36 | 279 | 290 | 15 | 68 | 11 | 75 | 6 | 17 | 13 | 0 | 24 | 2 | 11 | 49 |
| 1980 | 41 | 221 | 262 | 7 | 75 | 19 | 76 | 29 | 40 | 13 | 0 | 13 | 3 | 8 | 50 |
| 1981 | 46 | 276 | 333 | 26 | 79 | 15 | 118 | 74 | 34 | 17 | 0 | 21 | 3 | 15 | 52 |
| 1982 | 36 | 284 | 289 | 26 | 82 | 14 | 96 | 84 | 29 | 11 | 0 | 20 | 4 | 10 | 52 |
| 1983 | 48 | 321 | 351 | 20 | 89 | 9 | 91 | 95 | 57 | 11 | 0 | 36 | 4 | 18 | 49 |
| 1984 | 44 | 354 | 380 | 16 | 78 | 11 | 79 | 78 | 40 | 8 | 0 | 24 | 14 | 25 | 54 |
| 1985 | 49 | 407 | 335 | 16 | 114 | 14 | 119 | 133 | 42 | 8 | 0 | 35 | 12 | 22 | 39 |
| 1986 | 74 | 483 | 462 | 30 | 124 | 21 | 157 | 120 | 77 | 7 | 0 | 44 | 13 | 38 | 58 |
| 1987 | 49 | 402 | 398 | 8 | 130 | 19 | 100 | 158 | 83 | 9 | 0 | 62 | 12 | 47 | 68 |
| 1988 | 55 | 422 | 376 | 20 | 166 | 11 | 120 | 88 | 80 | 11 | 0 | 65 | 10 | 68 | 59 |
| 1989 | 71 | 428 | 393 | 20 | 152 | 19 | 189 | 57 | 72 | 6 | 0 | 39 | 8 | 72 | 54 |
| 1990 | 77 | 440 | 364 | 16 | 185 | 31 | 134 | 78 | 59 | 6 | 0 | 39 | 24 | 91 | 33 |
| 1991 | 62 | 476 | 481 | 10 | 212 | 19 | 131 | 127 | 92 | 7 | 0 | 66 | 13 | 119 | 43 |
| 1992 | 77 | 425 | 536 | 12 | 265 | 29 | 187 | 111 | 94 | 7 | 0 | 51 | 26 | 127 | 68 |
| 1993 | 72 | 567 | 552 | 15 | 230 | 29 | 133 | 105 | 89 | 7 | 0 | 61 | 15 | 189 | 56 |
| 1994 | 86 | 452 | 531 | 13 | 236 | 20 | 166 | 69 | 77 | 9 | 0 | 65 | 36 | 139 | 60 |
| 1995 | 66 | 506 | 594 | 14 | 229 | 25 | 155 | 167 | 150 | 13 | 0 | 60 | 17 | 109 | 62 |
| 1996 | 81 | 589 | 676 | 25 | 333 | 33 | 122 | 103 | 95 | 8 | 0 | 80 | 53 | 178 | 53 |
| 1997 | 82 | 581 | 767 | 22 | 257 | 23 | 151 | 122 | 137 | 13 | 0 | 120 | 31 | 147 | 63 |
| 1998 | 111 | 544 | 676 | 22 | 263 | 27 | 151 | 118 | 101 | 8 | 0 | 97 | 36 | 139 | 44 |
| 1999 | 89 | 614 | 664 | 31 | 212 | 23 | 97 | 95 | 119 | 7 | 0 | 94 | 34 | 127 | 39 |
| 2000 | 76 | 547 | 686 | 48 | 277 | 26 | 123 | 83 | 102 | 15 | 0 | 118 | 30 | 168 | 67 |
| 2001 | 102 | 514 | 695 | 31 | 149 | 34 | 101 | 114 | 89 | 11 | 0 | 108 | 67 | 122 | 51 |
| 2002 | 91 | 538 | 651 | 43 | 180 | 29 | 156 | 105 | 112 | 12 | 0 | 91 | 77 | 133 | 63 |
| 2003 | 90 | 570 | 756 | 72 | 214 | 34 | 106 | 113 | 118 | 13 | 0 | 105 | 86 | 124 | 61 |
| 2004 | 95 | 563 | 687 | 54 | 212 | 33 | 118 | 112 | 142 | 11 | 1 | 110 | 81 | 163 | 58 |
| 2005 | 101 | 552 | 806 | 81 | 206 | 48 | 166 | 166 | 166 | 16 | 3 | 142 | 110 | 243 | 60 |
| 2006 | 70 | 549 | 836 | 116 | 185 | 46 | 182 | 175 | 138 | 14 | 9 | 138 | 97 | 169 | 60 |
| 2007 | 120 | 621 | 930 | 147 | 219 | 63 | 150 | 143 | 177 | 15 | 2 | 184 | 126 | 220 | 46 |
| 2008 | 134 | 714 | 912 | 166 | 180 | 69 | 187 | 167 | 192 | 22 | 6 | 220 | 108 | 238 | 37 |
| 2009 | 160 | 673 | 952 | 200 | 222 | 61 | 147 | 203 | 167 | 22 | 6 | 290 | 145 | 249 | 52 |
| 2010 | 160 | 809 | 1164 | 239 | 237 | 69 | 197 | 171 | 199 | 26 | 2 | 303 | 211 | 218 | 65 |
| 2011 | 190 | 759 | 1144 | 285 | 231 | 81 | 208 | 173 | 228 | 20 | 8 | 307 | 162 | 252 | 34 |
| 2012 | 194 | 762 | 1165 | 317 | 238 | 85 | 228 | 195 | 242 | 22 | 4 | 346 | 177 | 292 | 53 |
| 2013 | 195 | 827 | 1309 | 375 | 270 | 93 | 214 | 180 | 201 | 27 | 12 | 371 | 185 | 287 | 72 |
| 2014 | 181 | 842 | 1241 | 434 | 272 | 112 | 233 | 173 | 240 | 34 | 10 | 379 | 193 | 327 | 59 |
| 2015 | 226 | 769 | 1222 | 392 | 235 | 109 | 267 | 207 | 216 | 41 | 11 | 389 | 218 | 364 | 59 |
| 2016 | 188 | 791 | 1171 | 403 | 258 | 144 | 326 | 167 | 219 | 35 | 15 | 341 | 219 | 399 | 60 |
| 2017 | 232 | 815 | 1221 | 399 | 278 | 112 | 314 | 203 | 186 | 47 | 39 | 393 | 213 | 399 | 66 |
| 2018 | 271 | 727 | 1157 | 480 | 303 | 107 | 259 | 145 | 171 | 80 | 72 | 359 | 222 | 398 | 63 |
| 2019 | 295 | 815 | 1272 | 491 | 300 | 137 | 349 | 199 | 251 | 101 | 106 | 442 | 270 | 425 | 71 |
| 2020 | 377 | 881 | 1318 | 625 | 344 | 141 | 393 | 148 | 265 | 88 | 127 | 492 | 321 | 546 | 52 |
| 2021 | 421 | 985 | 1421 | 708 | 340 | 146 | 435 | 197 | 333 | 99 | 184 | 553 | 317 | 580 | 55 |
| 2022 | 389 | 854 | 1302 | 670 | 311 | 95 | 311 | 170 | 322 | 69 | 181 | 558 | 396 | 568 | 75 |
| 2023 | 415 | 822 | 1147 | 813 | 348 | 122 | 343 | 156 | 339 | 78 | 184 | 455 | 385 | 530 | 48 |
| 2024 | 458 | 843 | 1166 | 770 | 386 | 148 | 266 | 148 | 319 | 74 | 173 | 527 | 363 | 552 | 64 |
| 2025 | 195 | 351 | 421 | 295 | 150 | 63 | 108 | 64 | 129 | 29 | 74 | 172 | 161 | 245 | 28 |

# **SUPPLEMENTARY TABLE 4** Top 100 publications according to total citation count (TCC) by June 1, 2025.

| ***Rank*** | ***TCC*** | ***Author, year*** | ***Title*** |
| --- | --- | --- | --- |
| 1 | 744 | Theilade et al., 1966 [1] | Experimental gingivitis in man. II. A longitudinal clinical and bacteriological investigation. |
| 2 | 740 | R.C. Page, 1991 [2] | The role of inflammatory mediators in the pathogenesis of periodontal disease. |
| 3 | 649 | Löe and Schiøtt, 1970 [3] | The effect of mouthrinses and topical application of chlorhexidine on the development of dental plaque and gingivitis in man. |
| 4 | 530 | H. Birkedal-Hansen, 1993 [4] | Role of cytokines and inflammatory mediators in tissue destruction. |
| 5 | 443 | Offenbacher et al., 1986 [5] | The use of crevicular fluid prostaglandin E2 levels as a predictor of periodontal attachment loss. |
| 6 | 405 | Golub et al., 1983 [6] | Minocycline reduces gingival collagenolytic activity during diabetes. Preliminary observations and a proposed new mechanism of action. |
| 7 | 353 | Masada et al., 1990 [7] | Measurement of interleukin-1 alpha and -1 beta in gingival crevicular fluid: implications for the pathogenesis of periodontal disease. |
| 8 | 349 | Kornman and Loesche, 1980 [8] | The subgingival microbial flora during pregnancy. |
| 9 | 346 | Yaegaki and Sanada, 1992 [9] | Volatile sulfur compounds in mouth air from clinically healthy subjects and patients with periodontal disease. |
| 10 | 342 | Dreyer et al., 2018 [10] | Epidemiology and risk factors of peri-implantitis: A systematic review. |
| 11 | 335 | Newman and Socransky, 1977 [11] | Predominant cultivable microbiota in periodontosis. |
| 12 | 324 | W.E.C. Moore, 1987 [12] | Microbiology of periodontal disease. |
| 13 | 314 | Lindhe et al., 1975 [13] | Plaque induced periodontal disease in beagle dogs. A 4-year clinical, roentgenographical and histometrical study. |
| 14 | 310 | Nagatomo et al., 2006 [14] | Stem cell properties of human periodontal ligament cells. |
| 15 | 294 | Glavind and Löe, 1967 [15] | Errors in the clinical assessment of periodontal destruction. |
| 16 | 285 | McCulloch and Bordin, 1991 [16] | Role of fibroblast subpopulations in periodontal physiology and pathology. |
| 17 | 280 | Socransky et al., 1977 [17] | Bacteriological studies of developing supragingival dental plaque. |
| 18 | 275 | Golub et al., 1984 [18] | Tetracyclines inhibit tissue collagenase activity. A new mechanism in the treatment of periodontal disease. |
| 19 | 271 | Crotti et al., 2003 [19] | Receptor activator NF kappaB ligand (RANKL) and osteoprotegerin (OPG) protein expression in periodontitis. |
| 20 | 268 | Mousquès et al., 1980 [20] | Effect of scaling and root planing on the composition of the human subgingival microbial flora. |
| 21 | 266 | Lee et al., 1995 [21] | Evidence of a direct relationship between neutrophil collagenase activity and periodontal tissue destruction in vivo: role of active enzyme in human periodontitis. |
| 22 | 265 | Karring et al., 1975 [22] | The role of gingival connective tissue in determining epithelial differentiation. |
| 23 | 264 | Lindhe et al., 1973 [23] | Experimental periodontitis in the beagle dog. |
| 24 | 256 | Camargo et al., 2002 [24] | Platelet-rich plasma and bovine porous bone mineral combined with guided tissue regeneration in the treatment of intrabony defects in humans. |
| 25 | 254 | Ferreira et al., 2017 [25] | Impact of periodontal disease on quality of life: a systematic review. |
| 26 | 252 | Löe et al.,1976 [26] | Two years oral use of chlorhexidine in man. |
| 27 | 251 | Akizuki et al., 2005 [27] | Application of periodontal ligament cell sheet for periodontal regeneration: a pilot study in beagle dogs. |
| 28 | 245 | Seymour et al., 1993 [28] | Immunopathogenesis of chronic inflammatory periodontal disease: cellular and molecular mechanisms. |
| 29 | 244 | Apse et al., 1989 [29] | Microbiota and crevicular fluid collagenase activity in the osseointegrated dental implant sulcus: a comparison of sites in edentulous and partially edentulous patients. |
| 30 | 241 | Garlet et al., 2003 [30] | Patterns of chemokines and chemokine receptors expression in different forms of human periodontal disease. |
| 31 | 236 | Dale et al., 2001 [31] | Localized antimicrobial peptide expression in human gingiva. |
| 32 | 235 | Gjermo et al., 1970 [32] | The plaque-inhibiting capacity of 11 antibacterial compounds. |
| 33 | 233 | Rölla et al., 1970 [33] | The affinity of chlorhexidine for hydroxyapatite and salivary mucins. |
| 34 | 229 | Schmidt et al., 1996 [34] | Advanced glycation endproducts (AGEs) induce oxidant stress in the gingiva: a potential mechanism underlying accelerated periodontal disease associated with diabetes. |
| 35 | 228 | Tsai et al., 2005 [35] | Lipid peroxidation: a possible role in the induction and progression of chronic periodontitis. |
| 36 | 227 | H. Takayanagi, 2018 [36] | Inflammatory bone destruction and osteoimmunology. |
| 37 | 226 | Savitt and Socransky, 1984 [37] | Distribution of certain subgingival microbial species in selected periodontal conditions. |
| 38 | 225 | Mackler et al., 1977 [38] | Immunoglobulin bearing lymphocytes and plasma cells in human periodontal disease. |
| 39 | 220 | Amano et al., 2004 [39] | Variations of Porphyromonas gingivalis fimbriae in relation to microbial pathogenesis. |
| 40 | 216 | Cole et al., 1980 [40] | Connective tissue regeneration to periodontally diseased teeth. |
| 41 | 215 | J. Slots, 2002 [41] | Selection of antimicrobial agents in periodontal therapy. |
| 42 | 214 | Sculean et al., 1999 [42] | Healing of human intrabony defects following treatment with enamel matrix proteins or guided tissue regeneration. |
| 43 | 212 | Murakami et al., 1999 [43] | Regeneration of periodontal tissues by basic fibroblast growth factor. |
| 44 | 210 | Lavine et al., 1979 [44] | Impaired neutrophil chemotaxis in patients with juvenile and rapidly progressing periodontitis. |
| 45 | 209 | R. Attström, 1970 [45] | Presence of leukocytes in crevices of healthy and chronically inflamed gingivae. |
| 46 | 206 | Murakami et al., 2003 [46] | Recombinant human basic fibroblast growth factor (bFGF) stimulates periodontal regeneration in class II furcation defects created in beagle dogs. |
| 47 | 204 | Golub et al., 1990 [47] | Low-dose doxycycline therapy: effect on gingival and crevicular fluid collagenase activity in humans. |
| 48 | 202 | Curtis et al., 1999 [48] | Molecular genetics and nomenclature of proteases of Porphyromonas gingivalis. |
| 49 | 201 | Rovin et al., 1966 [49] | The influence of bacteria and irritation in the initiation of periodontal disease in germfree and conventional rats. |
| 50 (tie) | 200 | Golub et al., 1985 [50] | Further evidence that tetracyclines inhibit collagenase activity in human crevicular fluid and from other mammalian sources. |
| 50 (tie) | 200 | Offenbacher et al., 1984 [51] | Crevicular fluid prostaglandin E levels as a measure of the periodontal disease status of adult and juvenile periodontitis patients. |
| 52 (tie) | 199 | Socransky and Haffajee, 1991 [52] | Microbial mechanisms in the pathogenesis of destructive periodontal diseases: a critical assessment. |
| 52 (tie) | 199 | Casarin et al., 2013 [53] | Subgingival biodiversity in subjects with uncontrolled type-2 diabetes and chronic periodontitis. |
| 54 | 198 | Noronha Oliveira et al., 2018 [54] | Can degradation products released from dental implants affect peri-implant tissues? |
| 55 (tie) | 197 | Rutherford et al., 1992 [55] | Platelet-derived and insulin-like growth factors stimulate regeneration of periodontal attachment in monkeys. |
| 55 (tie) | 197 | Heasman et al., 1993 [56] | Changes in crevicular fluid levels of interleukin-1 beta, leukotriene B4, prostaglandin E2, thromboxane B2 and tumour necrosis factor alpha in experimental gingivitis in humans. |
| 57 | 195 | Giannobile et al., 1996 [57] | Comparative effects of platelet-derived growth factor-BB and insulin-like growth factor-I, individually and in combination, on periodontal regeneration in Macaca fascicularis. |
| 58 | 194 | Lang and Brecx, 1986 [58] | Chlorhexidine digluconate–an agent for chemical plaque control and prevention of gingival inflammation. |
| 59 | 193 | D'Aiuto et al., 2004 [59] | Periodontal disease and C-reactive protein-associated cardiovascular risk. |
| 60 (tie) | 191 | Hönig et al., 1989 [60] | Increased interleukin-1 beta (IL-1 beta) concentration in gingival tissue from periodontitis patients. |
| 60 (tie) | 191 | Payne et al., 1975 [61] | Histopathologic features of the initial and early stages of experimental gingivitis in man. |
| 62 | 189 | Gould et al., 1980 [62] | Migration and division of progenitor cell populations in periodontal ligament after wounding. |
| 63 | 187 | Mäntylä et al., 2003 [63] | Gingival crevicular fluid collagenase-2 (MMP-8) test stick for chair-side monitoring of periodontitis. |
| 64 (tie) | 185 | Saito et al., 2005 [64] | Relationship between obesity, glucose tolerance, and periodontal disease in Japanese women: the Hisayama study. |
| 64 (tie) | 185 | Jordan et al., 1972 [65] | Periodontal lesions in hamsters and gnotobiotic rats infected with actinomyces of human origin. |
| 66 | 183 | Baelum et al., 1986 [66] | Oral hygiene, gingivitis and periodontal breakdown in adult Tanzanians. |
| 67 | 182 | Pitaru et al., 1994 [67] | Cellular origins and differentiation control mechanisms during periodontal development and wound healing. |
| 68 | 180 | Ara et al., 2009 [68] | Human gingival fibroblasts are critical in sustaining inflammation in periodontal disease. |
| 69 (tie) | 179 | Ruttimann et al., 1986 [69] | A robust digital method for film contrast correction in subtraction radiography. |
| 69 (tie) | 179 | Neiders et al., 1989 [70] | Heterogeneity of virulence among strains of Bacteroides gingivalis. |
| 71 | 178 | White and Mayrand, 1981 [71] | Association of oral Bacteroides with gingivitis and adult periodontitis. |
| 72 (tie) | 175 | Hirasawa et al., 2002 [72] | Improvement of periodontal status by green tea catechin using a local delivery system: a clinical pilot study. |
| 72 (tie) | 175 | Seymour and Greenspan, 1979 [73] | The phenotypic characterization of lymphocyte subpopulations in established human periodontal disease. |
| 72 (tie) | 175 | Sorsa et al., 1988 [74] | Comparison of interstitial collagenases from human gingiva, sulcular fluid and polymorphonuclear leukocytes. |
| 75 (tie) | 174 | Garrett et al., 1978 [75] | Effects of citric acid on diseased root surfaces. |
| 75 (tie) | 174 | Fontana et al., 2009 [76] | The antibacterial effect of photodynamic therapy in dental plaque-derived biofilms. |
| 77 | 170 | Schiøtt et al., 1970 [77] | The effect of chlorhexidine mouthrinses on the human oral flora. |
| 78 (tie) | 169 | de Jong et al., 2017 [78] | The intricate anatomy of the periodontal ligament and its development: Lessons for periodontal regeneration. |
| 78 (tie) | 169 | Kenney et al., 1977 [79] | The effect of cigarette smoke on human oral polymorphonuclear leukocytes. |
| 80 | 167 | Karring et al.,1971 [80] | Conservation of tissue specificity after heterotopic transplantation of gingiva and alveolar mucosa. |
| 81 | 166 | Nyman et al., 1987 [81] | New attachment formation by guided tissue regeneration. |
| 82 | 165 | Nojima et al., 1990 [82] | Fibroblastic cells derived from bovine periodontal ligaments have the phenotypes of osteoblasts. |
| 83 (tie) | 164 | Zhang et al., 2021 [83] | Hyperglycemia modulates M1/M2 macrophage polarization via reactive oxygen species overproduction in ligature-induced periodontitis. |
| 83 (tie) | 164 | Tanner et al., 1984 [84] | Microbiota of periodontal pockets losing crestal alveolar bone. |
| 83 (tie) | 164 | Salvi et al., 1998 [85] | Inflammatory mediators of the terminal dentition in adult and early onset periodontitis. |
| 86 (tie) | 163 | Boyko et al., 1981 [86] | Formation of new periodontal ligament by periodontal ligament cells implanted in vivo after culture in vitro. A preliminary study of transplanted roots in the dog. |
| 86 (tie) | 163 | Terranova et al., 1986 [87] | A biochemical approach to periodontal regeneration: tetracycline treatment of dentin promotes fibroblast adhesion and growth. |
| 88 | 162 | Gabler and Creamer, 1991 [88] | Suppression of human neutrophil functions by tetracyclines. |
| 89 (tie) | 161 | M.A. Listgarten, 1987 [89] | Nature of periodontal diseases: pathogenic mechanisms. |
| 89 (tie) | 161 | Crigger et al., 1978 [90] | Crigger M, Bogle G, Nilvéus R, Egelberg J, Selvig KA. The effect of topical citric acid application on the healing of experimental furcation defects in dogs. |
| 91 (tie) | 160 | Yamazaki et al., 2005 [91] | Effect of periodontal treatment on the C-reactive protein and proinflammatory cytokine levels in Japanese periodontitis patients. |
| 91 (tie) | 160 | Saxton and van der Ouderaa, 1989 [92] | The effect of a dentifrice containing zinc citrate and Triclosan on developing gingivitis. |
| 91 (tie) | 160 | Wikesjö et al., 1986 [93] | A biochemical approach to periodontal regeneration: tetracycline treatment conditions dentin surfaces. |
| 94 | 158 | Robertson et al., 1982 [94] | Collagenolytic activity associated with Bacteroides species and Actinobacillus actinomycetemcomitans. |
| 95 (tie) | 157 | Contreras and Slots, 2000 [95] | Herpesviruses in human periodontal disease. |
| 95 (tie) | 157 | Goodson et al., 1991 [96] | Multicenter evaluation of tetracycline fiber therapy: II. Clinical response. |
| 97 | 156 | Jönsson et al., 2011 [97] | The human periodontal ligament cell: a fibroblast-like cell acting as an immune cell. |
| 98 (tie) | 155 | Schroeder and Theilade, 1966 [98] | Electron microscopy of normal human gingival epithelium. |
| 98 (tie) | 155 | Wilson et al., 1996 [99] | Cytokine-inducing components of periodontopathogenic bacteria. |
| 100 | 154 | Jeffcoat et al., 1996 [100] | Extraoral control of geometry for digital subtraction radiography. |

# **SUPPLEMENTARY TABLE 5** Top 100 publications according to average citation per year (ACY) by June 1, 2025 (TCC: Total Citation Count).

| ***Rank*** | ***ACY*** | ***TCC*** | ***Author, year*** | ***Title*** |
| --- | --- | --- | --- | --- |
| 1 | 48.86 | 342 | Dreyer et al.,2018 [10] | Epidemiology and risk factors of peri-implantitis: A systematic review. |
| 2 | 46.00 | 46 | Nascimento et al., 2024 [101] | Burden of severe periodontitis and edentulism in 2021, with projections up to 2050: The Global Burden of Disease 2021 study. |
| 3 | 41.00 | 164 | Zhang et al., 2021 [83] | Hyperglycemia modulates M1/M2 macrophage polarization via reactive oxygen species overproduction in ligature-induced periodontitis. |
| 4 | 37.00 | 37 | Isola et al., 2024 [102] | Effect of quadrantwise versus full-mouth subgingival instrumentation on clinical and microbiological parameters in periodontitis patients: A randomized clinical trial. |
| 5 | 31.75 | 254 | Ferreira et al., 2017 [25] | Impact of periodontal disease on quality of life: a systematic review. |
| 6 | 28.29 | 198 | Noronha Oliveira et al., 2018 [54] | Can degradation products released from dental implants affect peri-implant tissues? |
| 7 | 28.00 | 56 | Isola et al., 2023 [103] | Impact of periodontitis on gingival crevicular fluid miRNAs profiles associated with cardiovascular disease risk. |
| 8 | 21.76 | 740 | R.C. Page, 1991 [2] | The role of inflammatory mediators in the pathogenesis of periodontal disease. |
| 9 | 21.13 | 169 | de Jong et al., 2017 [78] | The intricate anatomy of the periodontal ligament and its development: Lessons for periodontal regeneration. |
| 10 | 19.50 | 78 | Wong et al., 2021 [104] | Periodontal disease and quality of life: Umbrella review of systematic reviews. |
| 11 | 18.50 | 148 | Pettersson et al., 2017 [39] | Titanium ions form particles that activate and execute interleukin-1β release from lipopolysaccharide-primed macrophages. |
| 12 | 18.25 | 73 | Isola et al., 2021 [105] | Analysis of galectin-3 levels as a source of coronary heart disease risk during periodontitis. |
| 13 | 17.75 | 142 | Long et al., 2017 [106] | Association of oral microbiome with type 2 diabetes risk. |
| 14 | 17.40 | 87 | Isola et al., 2020 [107] | Association of vitamin D in patients with periodontitis: A cross-sectional study. |
| 15 | 16.58 | 199 | Casarin et al., 2013 [53] | Subgingival biodiversity in subjects with uncontrolled type-2 diabetes and chronic periodontitis. |
| 16 | 16.56 | 530 | H. Birkedal-Hansen, 1993 [4] | Role of cytokines and inflammatory mediators in tissue destruction. |
| 17 | 16.50 | 33 | Antezack et al., 2023 [108] | New putative periodontopathogens and periodontal health-associated species: A systematic review and meta-analysis. |
| 18 | 16.32 | 310 | Nagatomo et al., 2006 [14] | Stem cell properties of human periodontal ligament cells. |
| 19 | 16.00 | 64 | Zheng et al., 2021 [109] | Porphyromonas gingivalis survival skills: Immune evasion. |
| 20 | 15.50 | 31 | E.M. Lu, 2023 [110] | The role of vitamin D in periodontal health and disease. |
| 21 | 15.40 | 77 | Lafuente Ibáñez de Mendoza et al., 2020 [111] | Role of Porphyromonas gingivalis in oral squamous cell carcinoma development: A systematic review. |
| 22 | 15.00 | 15 | Wu et al., 2024 [112] | Association between Mediterranean diet and periodontitis among US adults: The mediating roles of obesity indicators. |
| 23 | 14.88 | 119 | Apaza-Bedoya et al., 2017 [113] | Synergistic interactions between corrosion and wear at titanium-based dental implant connections: A scoping review. |
| 24 | 14.33 | 43 | Mohammad-Rahimi et al., 2022 [114] | Deep learning in periodontology and oral implantology: A scoping review. |
| 25 (tie) | 14.00 | 112 | Danesh-Sani et al., 2017 [115] | Histomorphometric results of different grafting materials and effect of healing time on bone maturation after sinus floor augmentation: a systematic review and meta-analysis. |
| 25 (tie) | 14.00 | 56 | Tsuzuno et al., 2021 [116] | Ingestion of Porphyromonas gingivalis exacerbates colitis via intestinal epithelial barrier disruption in mice. |
| 25 (tie) | 14.00 | 28 | Jiang et al., 2023 [117] | The role of mitochondrial dysfunction in periodontitis: From mechanisms to therapeutic strategy. |
| 28 | 13.67 | 41 | Han et al., 2022 [118] | The emerging role of small extracellular vesicles in saliva and gingival crevicular fluid as diagnostics for periodontitis. |
| 29 | 13.40 | 67 | de Avila et al., 2020 [119] | Biomaterial-based possibilities for managing peri-implantitis. |
| 30 | 13.29 | 93 | Galofré et al., 2018 [120] | Clinical and microbiological evaluation of the effect of Lactobacillus reuteri in the treatment of mucositis and peri-implantitis: A triple-blind randomized clinical trial. |
| 31 (tie) | 13.00 | 13 | Yang et al., 2024 [121] | Interleukin-37 ameliorates periodontitis development by inhibiting NLRP3 inflammasome activation and modulating M1/M2 macrophage polarization. |
| 31 (tie) | 13.00 | 39 | Deng et al., 2022 [122] | The Th17/Treg cell balance: crosstalk among the immune system, bone and microbes in periodontitis. |
| 31 (tie) | 13.00 | 91 | Nonaka et al., 2018 [123] | Advanced glycation end-products increase IL-6 and ICAM-1 expression via RAGE, MAPK and NF-κB pathways in human gingival fibroblasts. |
| 34 | 12.88 | 103 | Tassi et al., 2017 [124] | Efficacy of stem cells on periodontal regeneration: Systematic review of pre-clinical studies. |
| 35 | 12.70 | 127 | Keestra et al., 2015 [125] | Non-surgical periodontal therapy with systemic antibiotics in patients with untreated aggressive periodontitis: a systematic review and meta-analysis. |
| 36 (tie) | 12.60 | 63 | Pitzurra et al., 2020 [126] | Effects of L-PRF and A-PRF+ on periodontal fibroblasts in in vitro wound healing experiments. |
| 36 (tie) | 12.60 | 126 | Ji et al., 2015 [127] | Bacterial invasion and persistence: critical events in the pathogenesis of periodontitis? |
| 38 | 12.59 | 744 | Theilade et al., 1966 [1] | Experimental gingivitis in man. II. A longitudinal clinical and bacteriological investigation. |
| 39 | 12.55 | 251 | Akizuki et al., 2005 [27] | Application of periodontal ligament cell sheet for periodontal regeneration: a pilot study in beagle dogs. |
| 40 | 12.32 | 271 | Crotti et al., 2003 [19] | Receptor activator NF kappaB ligand (RANKL) and osteoprotegerin (OPG) protein expression in periodontitis. |
| 41 | 12.00 | 47 | Baima et al., 2021 [128] | Metabolomics of gingival crevicular fluid to identify biomarkers for periodontitis: A systematic review with meta-analysis. |
| 42 | 11.89 | 107 | Duarte et al., 2016 [129] | Could cytokine levels in the peri-implant crevicular fluid be used to distinguish between healthy implants and implants with peri-implantitis? A systematic review. |
| 43 | 11.83 | 71 | Iwasaki et al., 2019 [130] | Periodontitis, periodontal inflammation, and mild cognitive impairment: A 5-year cohort study. |
| 44 (tie) | 11.80 | 59 | Souza et al., 2020 [131] | Titanium particles and ions favor dysbiosis in oral biofilms. |
| 44 (tie) | 11.80 | 118 | Jin et al., 2015 [132] | Isolation and characterization of human mesenchymal stem cells from gingival connective tissue. |
| 44 (tie) | 11.80 | 649 | Löe and Schiøtt, 1970 [3] | The effect of mouthrinses and topical application of chlorhexidine on the development of dental plaque and gingivitis in man. |
| 47 | 11.70 | 117 | Sokos et al., 2015 [133] | Role of periodontal ligament fibroblasts in osteoclastogenesis: a review. |
| 48 (tie) | 11.50 | 69 | Asparuhova et al., 2019 [134] | Activity of two hyaluronan preparations on primary human oral fibroblasts. |
| 48 (tie) | 11.50 | 115 | Bright et al., 2015 [135] | Periodontal ligament-derived cells for periodontal regeneration in animal models: a systematic review. |
| 50 | 11.40 | 228 | Tsai et al., 2005 [35] | Lipid peroxidation: a possible role in the induction and progression of chronic periodontitis. |
| 51 | 11.36 | 443 | Offenbacher et al., 1986 [5] | The use of crevicular fluid prostaglandin E2 levels as a predictor of periodontal attachment loss. |
| 52 | 11.35 | 227 | H. Takayanagi, 2018 [36] | Inflammatory bone destruction and osteoimmunology. |
| 53 (tie) | 11.25 | 45 | Li et al., 2021 [136] | Curcumin: A review of experimental studies and mechanisms related to periodontitis treatment. |
| 53 (tie) | 11.25 | 180 | Ara et al., 2009 [68] | Human gingival fibroblasts are critical in sustaining inflammation in periodontal disease. |
| 55 | 11.14 | 156 | Jönsson et al., 2011 [97] | The human periodontal ligament cell: a fibroblast-like cell acting as an immune cell. |
| 56 | 11.13 | 256 | Camargo et al., 2002 [24] | Platelet-rich plasma and bovine porous bone mineral combined with guided tissue regeneration in the treatment of intrabony defects in humans. |
| 57 | 11.10 | 111 | Landzberg et al., 2015 [137] | Quantifying oral inflammatory load: oral neutrophil counts in periodontal health and disease. |
| 58 | 10.95 | 241 | Garlet et al., 2003 [30] | Patterns of chemokines and chemokine receptors expression in different forms of human periodontal disease. |
| 59 | 10.88 | 174 | Fontana et al., 2009 [76] | The antibacterial effect of photodynamic therapy in dental plaque-derived biofilms. |
| 60 | 10.75 | 129 | Harvey et al., 2013 [138] | Expression of peptidylarginine deiminase-2 and -4, citrullinated proteins and anti-citrullinated protein antibodies in human gingiva |
| 61 | 10.67 | 59 | Shimizu et al., 2022 [139] | Exosomes from dental pulp cells attenuate bone loss in mouse experimental periodontitis. |
| 62 | 10.60 | 53 | Aral et al., 2020 [140] | Inflammasomes and their regulation in periodontal disease: A review. |
| 63 | 10.56 | 95 | Algate et al., 2016 [141] | The effects of tumour necrosis factor-α on bone cells involved in periodontal alveolar bone loss; osteoclasts, osteoblasts and osteocytes. |
| 64 | 10.55 | 116 | Costa et al., 2014 [142] | Tooth loss in individuals under periodontal maintenance therapy: 5-year prospective study. |
| 65 | 10.50 | 42 | B.O. Nilsson, 2021 [143] | Mechanisms involved in regulation of periodontal ligament cell production of pro-inflammatory cytokines: Implications in periodontitis. |
| 66 (tie) | 10.48 | 220 | Amano et al., 2004 [39] | Variations of Porphyromonas gingivalis fimbriae in relation to microbial pathogenesis. |
| 66 (tie) | 10.48 | 346 | Yaegaki and Sanada, 1992 [9] | Volatile sulfur compounds in mouth air from clinically healthy subjects and patients with periodontal disease. |
| 68 | 10.25 | 82 | Corrêa et al., 2017 [144] | Systemic treatment with resveratrol and/or curcumin reduces the progression of experimental periodontitis in rats. |
| 69 | 10.22 | 92 | Bartold et al., 2016 [145] | Tissue engineered periodontal products. |
| 70 | 10.17 | 61 | Fawzy El-Sayed et al., 2019 [146] | The periodontal stem/progenitor cell inflammatory-regenerative cross talk: A new perspective. |
| 71 | 10.09 | 353 | Masada et al., 1990 [7] | Measurement of interleukin-1 alpha and -1 beta in gingival crevicular fluid: implications for the pathogenesis of periodontal disease. |
| 72 (tie) | 10.00 | 10 | Figueredo et al., 2024 [147] | Use of ultrasound imaging for assessment of the periodontium: A systematic review. |
| 72 (tie) | 10.00 | 10 | Ma et al., 2024 [148] | Resveratrol modulates the inflammatory response in hPDLSCs via the NRF2/HO-1 and NF-κB pathways and promotes osteogenic differentiation. |
| 72 (tie) | 10.00 | 20 | Chen et al., 2023 [149] | Cannabidiol attenuates periodontal inflammation through inhibiting TLR4/NF-κB pathway. |
| 72 (tie) | 10.00 | 50 | Liu et al., 2020 [150] | Long non-coding RNA and mRNA expression profiles in peri-implantitis vs periodontitis. |
| 72 (tie) | 10.00 | 60 | Wang et al., 2019 [151] | Macrophage polarization in aseptic bone resorption around dental implants induced by Ti particles in a murine model. |
| 72 (tie) | 10.00 | 120 | Kagiya and Nakamura, 2013 [152] | Expression profiling of microRNAs in RAW264.7 cells treated with a combination of tumor necrosis factor alpha and RANKL during osteoclast differentiation. |
| 78 | 9.83 | 236 | Dale et al., 2001 [31] | Localized antimicrobial peptide expression in human gingiva. |
| 79 | 9.64 | 405 | Golub et al., 1983 [6] | Minocycline reduces gingival collagenolytic activity during diabetes. Preliminary observations and a proposed new mechanism of action. |
| 80 (tie) | 9.50 | 19 | Antonoglou et al., 2023 | Periodontitis and edentulism as risk indicators for mortality: Results from a prospective cohort study with 20 years of follow-up. |
| 80 (tie) | 9.50 | 133 | Saygun et al., 2011 [153] | Salivary infectious agents and periodontal disease status. |
| 82 | 9.40 | 47 | Liu et al., 2020 [154] | LIPUS inhibited the expression of inflammatory factors and promoted the osteogenic differentiation capacity of hPDLCs by inhibiting the NF-κB signaling pathway. |
| 83 (tie) | 9.36 | 103 | Maekawa and Hajishengallis, 2014 [155] | Topical treatment with probiotic Lactobacillus brevis CD2 inhibits experimental periodontal inflammation and bone loss. |
| 83 (tie) | 9.36 | 206 | Murakami et al., 2003 [46] | Recombinant human basic fibroblast growth factor (bFGF) stimulates periodontal regeneration in class II furcation defects created in beagle dogs. |
| 85 | 9.35 | 215 | J. Slots, 2002 [41] | Selection of antimicrobial agents in periodontal therapy. |
| 86 (tie) | 9.25 | 37 | Guo et al., 2021 [156] | NCOA4-mediated ferritinophagy promoted inflammatory responses in periodontitis. |
| 86 (tie) | 9.25 | 185 | Saito et al., 2005 [64] | Relationship between obesity, glucose tolerance, and periodontal disease in Japanese women: the Hisayama study. |
| 88 | 9.22 | 83 | Mikkonen et al., 2016 [157] | Salivary metabolomics in the diagnosis of oral cancer and periodontal diseases. |
| 89 | 9.19 | 193 | D'Aiuto et al., 2004 [59] | Periodontal disease and C-reactive protein-associated cardiovascular risk. |
| 90 | 9.17 | 110 | Ertugrul et al., 2013 [158] | Comparison of CCL28, interleukin-8, interleukin-1β and tumor necrosis factor-alpha in subjects with gingivitis, chronic periodontitis and generalized aggressive periodontitis. |
| 91 | 9.14 | 64 | de Morais et al., 2018 [159] | Matrix metalloproteinase-8 levels in periodontal disease patients: A systematic review. |
| 92 (tie) | 9.00 | 144 | Teles et al., 2009 [160] | Salivary cytokine levels in subjects with chronic periodontitis and in periodontally healthy individuals: a cross-sectional study. |
| 92 (tie) | 9.00 | 54 | Albuquerque-Souza et al., 2019 [161] | Probiotics alter the immune response of gingival epithelial cells challenged by Porphyromonas gingivalis. |
| 92 (tie) | 9.00 | 27 | Deng et al., 2022 [162] | Diagnostic accuracy of active matrix metalloproteinase-8 point-of-care test for the discrimination of periodontal health status: Comparison of saliva and oral rinse samples. |
| 92 (tie) | 9.00 | 9 | Gao et al., 2024 [163] | Caspase-3 and gasdermin E mediate macrophage pyroptosis in periodontitis. |
| 92 (tie) | 9.00 | 9 | Pakpahan et al., 2024 [164] | Effects of mechanical loading on matrix homeostasis and differentiation potential of periodontal ligament cells: A scoping review. |
| 97 | 8.87 | 266 | Lee et al., 1995 [21] | Evidence of a direct relationship between neutrophil collagenase activity and periodontal tissue destruction in vivo: role of active enzyme in human periodontitis. |
| 98 | 8.85 | 115 | Lekovic et al., 2012 [165] | Platelet-rich fibrin and bovine porous bone mineral vs. platelet-rich fibrin in the treatment of intrabony periodontal defects. |
| 99 | 8.83 | 53 | Kang et al., 2019 [166] | Sequential application of bFGF and BMP-2 facilitates osteogenic differentiation of human periodontal ligament stem cells. |
| 100 | 8.71 | 61 | Calciolari et al., 2018 [167] | Degradation pattern of a porcine collagen membrane in an in vivo model of guided bone regeneration. |

# **SUPPLEMENTARY TABLE 6** Top 100 most prolific authors according to the number of publications by June 1, 2025.

| ***Rank*** | ***Author*** | ***Number of Documents*** |
| --- | --- | --- |
| 1 | Lindhe, J. | 52 |
| 2 | Löe, H. | 50 |
| 3 | Bartold, P.M. | 41 |
| 4 (tie) | Ebersole, J.L. | 38 |
| 4 (tie) | Page, R.C. | 38 |
| 6 | Egelberg, J. | 37 |
| 7 | Socransky, S.S. | 36 |
| 8 (tie) | Attström, R. | 34 |
| 8 (tie) | Yoshie, H. | 34 |
| 10 | Izumi, Y. | 33 |
| 11 | Hara, Y. | 32 |
| 12 | Lang, N.P. | 31 |
| 13 | Ishikawa, I. | 29 |
| 14 (tie) | Murakami, S. | 28 |
| 14 (tie) | Seymour, G.J. | 28 |
| 16 (tie) | Genco, R.J. | 26 |
| 16 (tie) | Sanz, M. | 26 |
| 16 (tie) | Slots, J. | 26 |
| 16 (tie) | Sorsa, T. | 26 |
| 20 (tie) | Kornman, K.S. | 25 |
| 20 (tie) | Offenbacher, S. | 25 |
| 20 (tie) | Williams, R.C. | 25 |
| 23 | Karring, T. | 24 |
| 24 (tie) | Garant, P.R. | 23 |
| 24 (tie) | Jeffcoat, M.K. | 23 |
| 24 (tie) | Listgarten, M.A. | 23 |
| 24 (tie) | Okada, H. | 23 |
| 28 (tie) | Donos, N. | 21 |
| 28 (tie) | Fu, E. | 21 |
| 28 (tie) | Hara, K. | 21 |
| 28 (tie) | Taubman, M.A. | 21 |
| 32 (tie) | Casati, M.Z. | 20 |
| 32 (tie) | Goldhaber, P. | 20 |
| 32 (tie) | Golub, L.M. | 20 |
| 32 (tie) | Kinane, D.F. | 20 |
| 32 (tie) | Kurihara, H. | 20 |
| 32 (tie) | Melcher, A.H. | 20 |
| 32 (tie) | Nagata, T. | 20 |
| 32 (tie) | Nociti, F.H. | 20 |
| 32 (tie) | Yamazaki, K. | 20 |
| 41 (tie) | Everts, V. | 19 |
| 41 (tie) | Ivanovski, S. | 19 |
| 41 (tie) | Pitaru, S. | 19 |
| 41 (tie) | Romandini, M. | 19 |
| 45 (tie) | Jin, L. | 18 |
| 45 (tie) | McCulloch, C.A.G. | 18 |
| 45 (tie) | Nuki, K. | 18 |
| 48 (tie) | Ito, K. | 17 |
| 48 (tie) | Robertson, P.B. | 17 |
| 50 (tie) | Beertsen, W. | 16 |
| 50 (tie) | Duarte, P.M. | 16 |
| 50 (tie) | Grenier, D. | 16 |
| 50 (tie) | Haffajee, A.D. | 16 |
| 50 (tie) | Hausmann, E. | 16 |
| 50 (tie) | Sallum, E.A. | 16 |
| 50 (tie) | Smith, D.J. | 16 |
| 50 (tie) | Ukai, T. | 16 |
| 58 (tie) | Chiang, C.Y. | 15 |
| 58 (tie) | Feres, M. | 15 |
| 58 (tie) | Maeda, K. | 15 |
| 58 (tie) | Nibali, L. | 15 |
| 58 (tie) | Noguchi, K. | 15 |
| 58 (tie) | Schenkein, H.A. | 15 |
| 58 (tie) | Sculean, A. | 15 |
| 58 (tie) | Smith, P.C. | 15 |
| 58 (tie) | Wolff, L.F. | 15 |
| 67 (tie) | Cao, Z. | 14 |
| 67 (tie) | Gjermo, P. | 14 |
| 67 (tie) | Goodson, J.M. | 14 |
| 67 (tie) | Kato, I. | 14 |
| 67 (tie) | Li, C. | 14 |
| 67 (tie) | Nakajima, T. | 14 |
| 67 (tie) | Noguchi, T. | 14 |
| 67 (tie) | Schroeder, H.E. | 14 |
| 67 (tie) | Stahl, S.S. | 14 |
| 67 (tie) | Taichman, N.S. | 14 |
| 67 (tie) | Teughels, W. | 14 |
| 67 (tie) | Tew, J.G. | 14 |
| 67 (tie) | Tipton, D.A. | 14 |
| 80 (tie) | Chang, Y.C. | 13 |
| 80 (tie) | Choi, S.H. | 13 |
| 80 (tie) | Ekuni, D. | 13 |
| 80 (tie) | Fujita, T. | 13 |
| 80 (tie) | Jacobsen, P.J. | 13 |
| 80 (tie) | Kitamura, M. | 13 |
| 80 (tie) | Powell, R.N. | 13 |
| 80 (tie) | Ranney, R.R. | 13 |
| 80 (tie) | Reinhardt, R.A. | 13 |
| 80 (tie) | Takashiba, S. | 13 |
| 80 (tie) | Tervahartiala, T. | 13 |
| 80 (tie) | Yamamoto, M. | 13 |
| 80 (tie) | Yoshinaga, Y. | 13 |
| 93 (tie) | Breivik, T. | 12 |
| 93 (tie) | Davies, R.M. | 12 |
| 93 (tie) | Herrera, D. | 12 |
| 93 (tie) | Holmstrup, P. | 12 |
| 93 (tie) | Ishihara, K. | 12 |
| 93 (tie) | Kaneko, T. | 12 |
| 93 (tie) | Patters, M.R. | 12 |
| 93 (tie) | Pavasant, P. | 12 |

# **SUPPLEMENTARY TABLE 7** List of all 86 countries contributing to *JPR* by June 1, 2025, sorted according to the number of publications.

| ***Rank*** | ***Institution*** | ***Number of Documents*** |
| --- | --- | --- |
| 1 | United States | 1399 |
| 2 | Japan | 725 |
| 3 | China | 353 |
| 4 | United Kingdom | 323 |
| 5 | Brazil | 241 |
| 6 | Sweden | 240 |
| 7 | Canada | 187 |
| 8 | Germany | 148 |
| 9 | Australia | 140 |
| 10 | Denmark | 123 |
| 11 | Norway | 122 |
| 12 | Turkey | 121 |
| 13 (tie) | Netherlands | 114 |
| 13 (tie) | Switzerland | 114 |
| 15 | South Korea | 95 |
| 16 | Taiwan | 94 |
| 17 | Finland | 92 |
| 18 | Italy | 82 |
| 19 | Spain | 70 |
| 20 | Israel | 64 |
| 21 | France | 59 |
| 22 | Hong Kong | 44 |
| 23 (tie) | Chile | 38 |
| 23 (tie) | India | 38 |
| 25 (tie) | New Zealand | 35 |
| 25 (tie) | Thailand | 35 |
| 27 | Belgium | 34 |
| 28 | Greece | 32 |
| 29 (tie) | Austria | 27 |
| 29 (tie) | South Africa | 27 |
| 31 (tie) | Argentina | 21 |
| 31 (tie) | Egypt | 21 |
| 31 (tie) | Singapore | 21 |
| 34 | Poland | 20 |
| 35 | Saudi Arabia | 19 |
| 36 | Mexico | 17 |
| 37 | Malaysia | 14 |
| 38 (tie) | Colombia | 12 |
| 38 (tie) | Iran | 12 |
| 40 (tie) | Hungary | 11 |
| 40 (tie) | Portugal | 11 |
| 42 | Romania | 9 |
| 43 (tie) | Ireland | 7 |
| 43 (tie) | Jordan | 7 |
| 45 | Peru | 6 |
| 46 (tie) | Ecuador | 5 |
| 46 (tie) | Indonesia | 5 |
| 46 (tie) | Serbia | 5 |
| 49 (tie) | Czech Republic | 4 |
| 49 (tie) | Iraq | 4 |
| 49 (tie) | United Arab Emirates | 4 |
| 49 (tie) | Vietnam | 4 |
| 53 (tie) | Puerto Rico | 3 |
| 53 (tie) | Türkiye | 3 |
| 55 (tie) | Kenya | 2 |
| 55 (tie) | Lebanon | 2 |
| 55 (tie) | Moldova | 2 |
| 55 (tie) | Nigeria | 2 |
| 55 (tie) | Pakistan | 2 |
| 55 (tie) | Slovenia | 2 |
| 55 (tie) | Sudan | 2 |
| 55 (tie) | Uruguay | 2 |
| 55 (tie) | Venezuela | 2 |
| 55 (tie) | Yemen | 2 |
| 65 (tie) | Algeria | 1 |
| 65 (tie) | Bolivia | 1 |
| 65 (tie) | Costa Rica | 1 |
| 65 (tie) | Croatia | 1 |
| 65 (tie) | Cuba | 1 |
| 65 (tie) | Czechoslovakia | 1 |
| 65 (tie) | Dominican Republic | 1 |
| 65 (tie) | Guatemala | 1 |
| 65 (tie) | Honduras | 1 |
| 65 (tie) | Kuwait | 1 |
| 65 (tie) | Libya | 1 |
| 65 (tie) | Malta | 1 |
| 65 (tie) | Morocco | 1 |
| 65 (tie) | Myanmar | 1 |
| 65 (tie) | Oman | 1 |
| 65 (tie) | Panama | 1 |
| 65 (tie) | Paraguay | 1 |
| 65 (tie) | Qatar | 1 |
| 65 (tie) | Russian Federation | 1 |
| 65 (tie) | Sri Lanka | 1 |
| 65 (tie) | Trinidad and Tobago | 1 |
| 65 (tie) | Ukraine | 1 |

# **SUPPLEMENTARY TABLE 8** Top 100 most prolific institutions according to the number of publications by June 1, 2025.

| ***Rank*** | ***Country*** | ***Number of Documents*** |
| --- | --- | --- |
| 1 | Forsyth Institute | 111 |
| 2 | University of Washington | 90 |
| 3 | Institut for Odontologi og Oral Sundhed | 88 |
| 4 (tie) | Harvard School of Dental Medicine | 79 |
| 4 (tie) | Institute of Science Tokyo | 79 |
| 6 | University of Toronto | 78 |
| 7 | Lunds Universitet | 73 |
| 8 | Universitetet i Oslo | 72 |
| 9 | Göteborgs Universitet | 71 |
| 10 | University at Buffalo, The State University of New York | 68 |
| 11 | The University of Queensland | 64 |
| 12 | The University of Osaka | 62 |
| 13 | University of Pennsylvania | 59 |
| 14 | Karolinska Institutet | 58 |
| 15 | Universidade Estadual de Campinas | 56 |
| 16 | Nihon University | 54 |
| 17 | University of Bern | 53 |
| 18 (tie) | Universidade Estadual Paulista Júlio de Mesquita Filho | 51 |
| 18 (tie) | Academisch Centrum Tandheelkunde Amsterdam | 51 |
| 20 (tie) | Universidade de São Paulo | 49 |
| 20 (tie) | The University of Adelaide | 49 |
| 22 | Barts and The London School of Medicine and Dentistry | 48 |
| 23 | Universiteit van Amsterdam | 47 |
| 24 (tie) | Helsingin Yliopisto | 46 |
| 24 (tie) | The University of Hong Kong | 46 |
| 24 (tie) | Kyushu University | 46 |
| 24 (tie) | Tokyo Dental College | 46 |
| 24 (tie) | The University of North Carolina at Chapel Hill | 46 |
| 29 | Stony Brook University | 45 |
| 30 (tie) | Sichuan University | 44 |
| 30 (tie) | Niigata University | 44 |
| 32 | Universitetet i Bergen | 43 |
| 33 (tie) | University of Michigan, Ann Arbor | 42 |
| 33 (tie) | West China School/Hospital of Stomatology Sichuan University | 42 |
| 35 (tie) | Tokushima University | 41 |
| 35 (tie) | University of Southern California | 41 |
| 35 (tie) | Niigata University, Graduate School of Medical and Dental Science | 41 |
| 38 (tie) | Tohoku University | 40 |
| 38 (tie) | University of Minnesota Twin Cities | 40 |
| 40 (tie) | Ministry of Education of the People's Republic of China | 38 |
| 40 (tie) | University of California, San Francisco | 38 |
| 40 (tie) | Peking University Hospital of Stomatology | 38 |
| 43 | Turun yliopisto | 37 |
| 44 | Eastman Dental Institute | 36 |
| 45 (tie) | Virginia Commonwealth University | 35 |
| 45 (tie) | Universität Zürich | 35 |
| 45 (tie) | Faculty of Life Sciences & Medicine | 35 |
| 48 (tie) | Vrije Universiteit Amsterdam | 34 |
| 48 (tie) | UNC-CH Adams School of Dentistry | 34 |
| 48 (tie) | University of Texas Health Science Center at Houston | 34 |
| 51 | Wuhan University | 33 |
| 52 (tie) | University of Otago | 32 |
| 52 (tie) | State Key Laboratory of Oral Disease | 32 |
| 54 (tie) | Hebrew University of Jerusalem | 31 |
| 54 (tie) | University of Kentucky | 31 |
| 54 (tie) | The University of Texas Health Science Center at San Antonio | 31 |
| 54 (tie) | University of Michigan School of Dentistry | 31 |
| 54 (tie) | Kanagawa Dental University | 31 |
| 59 (tie) | Nagasaki University | 30 |
| 59 (tie) | Universidad de Chile | 30 |
| 59 (tie) | University of Iowa | 30 |
| 59 (tie) | Universidad Complutense de Madrid | 30 |
| 59 (tie) | Malmo University Faculty of Odontology | 30 |
| 59 (tie) | School of Stomatology Wuhan University | 30 |
| 65 (tie) | Tel Aviv University | 29 |
| 65 (tie) | The University of British Columbia | 29 |
| 65 (tie) | Hokkaido University | 29 |
| 65 (tie) | School of Dental Medicine | 29 |
| 65 (tie) | University of Birmingham | 29 |
| 65 (tie) | Aichi Gakuin University | 29 |
| 71 (tie) | University of Kentucky College of Dentistry | 28 |
| 71 (tie) | University of Iowa College of Dentistry | 28 |
| 71 (tie) | University of Manitoba | 28 |
| 71 (tie) | Queen Mary University of London | 28 |
| 71 (tie) | Hiroshima University | 28 |
| 71 (tie) | Umeå Universitet | 28 |
| 71 (tie) | Shanghai Jiao Tong University School of Medicine | 28 |
| 78 (tie) | Okayama University | 27 |
| 78 (tie) | Shanghai Ninth People's Hospital, Shanghai JiaoTong University School of Medicine | 27 |
| 80 (tie) | King's College London | 26 |
| 80 (tie) | VA Medical Center | 26 |
| 80 (tie) | KU Leuven | 26 |
| 80 (tie) | The University of Alabama at Birmingham | 26 |
| 80 (tie) | Université Paris Cité | 26 |
| 85 (tie) | Eastman Institute for Oral Health | 25 |
| 85 (tie) | Helsinki University Hospital | 25 |
| 85 (tie) | Hammaslääketieteen laitos | 25 |
| 85 (tie) | Columbia University | 25 |
| 85 (tie) | Université Laval | 25 |
| 85 (tie) | Graduate School of Medical and Dental Sciences | 25 |
| 91 (tie) | Yonsei University | 24 |
| 91 (tie) | Kagoshima University | 24 |
| 91 (tie) | Yonsei University College of Dentistry | 24 |
| 91 (tie) | Graduate School of Medicine, Dentistry and Pharmaceutical Sciences | 24 |
| 95 (tie) | National Defense Medical Center Taiwan | 23 |
| 95 (tie) | Nippon Dental University | 23 |
| 95 (tie) | University of the Witwatersrand, Johannesburg | 23 |
| 95 (tie) | Glasgow Dental Hospital and School | 23 |
| 95 (tie) | Kyushu Dental College | 23 |
| 95 (tie) | University College London | 23 |

# **SUPPLEMENTARY TABLE 9** International collaboration pairs with ≥5 coauthored publications by June 1, 2025.

| ***Rank*** | ***Institution*** | ***Number of Documents*** |
| --- | --- | --- |
| 1 | Brazil & United States | 50 |
| 2 | Japan & United States | 48 |
| 3 | China & United States | 34 |
| 4 | Switzerland & United States | 29 |
| 5 (tie) | Turkey & United States | 25 |
| 5 (tie) | United Kingdom & United States | 25 |
| 7 | Sweden & United States | 24 |
| 8 | Denmark & Sweden | 23 |
| 9 | Israel & United States | 22 |
| 10 (tie) | Canada & United States | 21 |
| 10 (tie) | Denmark & United States | 21 |
| 12 | Germany & United States | 20 |
| 13 | China & Hong Kong | 18 |
| 14 | Taiwan & United States | 16 |
| 15 | China & Japan | 15 |
| 16 (tie) | Finland & United States | 14 |
| 16 (tie) | Spain & United States | 14 |
| 18 (tie) | Australia & United States | 12 |
| 18 (tie) | Brazil & Canada | 12 |
| 18 (tie) | Italy & United States | 12 |
| 21 (tie) | Brazil & Sweden | 11 |
| 21 (tie) | Canada & Japan | 11 |
| 21 (tie) | Finland & Sweden | 11 |
| 24 (tie) | Italy & United Kingdom | 10 |
| 24 (tie) | Norway & Sweden | 10 |
| 24 (tie) | Poland & United States | 10 |
| 24 (tie) | Sweden & Switzerland | 10 |
| 28 (tie) | Egypt & Germany | 9 |
| 28 (tie) | Finland & Turkey | 9 |
| 28 (tie) | Norway & United States | 9 |
| 31 (tie) | Australia & China | 8 |
| 31 (tie) | Canada & Finland | 8 |
| 31 (tie) | Germany & Switzerland | 8 |
| 31 (tie) | Greece & United Kingdom | 8 |
| 31 (tie) | Greece & United States | 8 |
| 31 (tie) | Japan & South Korea | 8 |
| 31 (tie) | Japan & Thailand | 8 |
| 31 (tie) | South Korea & United States | 8 |
| 39 (tie) | Canada & Israel | 7 |
| 39 (tie) | China & Switzerland | 7 |
| 39 (tie) | Japan & Taiwan | 7 |
| 39 (tie) | Saudi Arabia & United States | 7 |
| 43 (tie) | Australia & New Zealand | 6 |
| 43 (tie) | Belgium & United States | 6 |
| 43 (tie) | China & Italy | 6 |
| 43 (tie) | Egypt & United States | 6 |
| 43 (tie) | France & United States | 6 |
| 43 (tie) | Germany & Norway | 6 |
| 43 (tie) | Mexico & United States | 6 |
| 43 (tie) | Netherlands & United States | 6 |
| 51 (tie) | Belgium & Netherlands | 5 |
| 51 (tie) | Brazil & Germany | 5 |
| 51 (tie) | Canada & China | 5 |
| 51 (tie) | Chile & Peru | 5 |
| 51 (tie) | Chile & Spain | 5 |
| 51 (tie) | China & India | 5 |
| 51 (tie) | Finland & United Kingdom | 5 |
| 51 (tie) | India & United States | 5 |
| 51 (tie) | Israel & Switzerland | 5 |
| 51 (tie) | Italy & Switzerland | 5 |
| 51 (tie) | Japan & Norway | 5 |
| 51 (tie) | Japan & Switzerland | 5 |
| 51 (tie) | Malaysia & New Zealand | 5 |
| 51 (tie) | New Zealand & United Kingdom | 5 |
| 51 (tie) | Norway & Spain | 5 |
| 51 (tie) | Spain & Switzerland | 5 |
| 51 (tie) | Switzerland & Turkey | 5 |
| 51 (tie) | Turkey & United Kingdom | 5 |

# **SUPPLEMENTARY TABLE 10** Under- and over-represented research topics in each of the five most prolific countries according to the analysis of residuals (*r*) by June 1, 2025.

|  | ***Research Topics*** | | | | | | | | | | | | | | |
| --- | --- | --- | --- | --- | --- | --- | --- | --- | --- | --- | --- | --- | --- | --- | --- |
| ***Country*** | ***1*** | ***2*** | ***3*** | ***4*** | ***5*** | ***6*** | ***7*** | ***8*** | ***9*** | ***10*** | ***11*** | ***12*** | ***13*** | ***14*** | ***15*** |
| The Unites States | -0.26 | **3.31** | -1.15 | **-2.01** | **3.54** | -0.13 | 1.22 | 1.20 | 1.39 | 0.52 | 0.82 | **-3.16** | **-3.06** | -0.65 | -0.35 |
| Japan | -1.85 | -1.02 | **4.10** | -1.75 | **-2.98** | -1.01 | -1.12 | 1.78 | -0.04 | -0.48 | -1.96 | 1.74 | -0.89 | -1.21 | **2.88** |
| China | -0.38 | **-4.20** | **-2.63** | **4.47** | **-2.54** | -0.99 | -1.15 | **-2.51** | **-2.01** | -0.66 | 0.64 | **5.04** | **8.53** | 1.90 | **-2.48** |
| The United Kingdom | **2.42** | 1.37 | 0.06 | **-2.24** | **3.31** | **3.52** | 1.25 | -1.68 | -0.04 | -0.91 | -1.48 | -0.48 | -1.79 | **-2.95** | -0.03 |
| Brazil | 1.74 | -1.67 | -1.56 | **4.47** | **-2.99** | -0.57 | -0.61 | -0.76 | -0.38 | 1.63 | **2.60** | **-2.15** | -0.63 | **4.60** | -1.20 |
| * *p* < 0.05  Topics: 1: Epidemiology, Risk, & Public Health; 2: Microbiology & Biofilm Ecology; 3: Host Response & Inflammatory Biomarkers; 4: Systemic Links & Comorbidities; 5: Diagnostics & Measurement; 6: Non-Surgical & Resective/Access Periodontal Therapy; 7: Antimicrobial & Adjunctive Strategies; 8: Regenerative Periodontal Therapy; 9: Biomaterials & Biologics; 10: Soft & Hard Tissue Augmentation; 11: Placement & Maintenance of Dental Implants; 12: Tissue Engineering & Cell-Based Therapies; 13: Genetics, Epigenetics, & Multi-Omics; 14: Host-Modulation & Pharmacologic Interventions; 15: Miscellaneous. | | | | | | | | | | | | | | | |

# **SUPPLEMENTARY TABLE 11** Top 100 most prolific funding agencies according to number of publications by June 1, 2025.

| ***Rank*** | ***Institution*** | ***Number of Documents*** |
| --- | --- | --- |
| 1 | National Institute of Dental and Craniofacial Research | 398 |
| 2 | Japan Society for the Promotion of Science | 164 |
| 3 | National Natural Science Foundation of China | 155 |
| 4 | National Institutes of Health | 49 |
| 5 | Conselho Nacional de Desenvolvimento Científico e Tecnológico | 38 |
| 6 (tie) | Coordenação de Aperfeiçoamento de Pessoal de Nível Superior | 36 |
| 6 (tie) | Fundação de Amparo à Pesquisa do Estado de São Paulo | 36 |
| 8 | National Research Foundation of Korea | 25 |
| 9 | Ministry of Education, Culture, Sports, Science and Technology | 21 |
| 10 | National Center for Research Resources | 17 |
| 11 | National Institute of General Medical Sciences | 14 |
| 12 | Osteology Foundation | 12 |
| 13 | National Key Research and Development Program of China | 11 |
| 14 (tie) | Australian Dental Research Foundation | 10 |
| 14 (tie) | China Postdoctoral Science Foundation | 10 |
| 16 (tie) | Ministry of Education, Science and Technology | 9 |
| 16 (tie) | Ministry of Science, ICT and Future Planning | 9 |
| 18 (tie) | Canadian Institutes of Health Research | 8 |
| 18 (tie) | Ministry of Science and Technology, Taiwan | 8 |
| 18 (tie) | National Heart, Lung, and Blood Institute | 8 |
| 21 (tie) | Fundamental Research Funds for the Central Universities | 7 |
| 21 (tie) | National Institute of Allergy and Infectious Diseases | 7 |
| 21 (tie) | National Institute of Arthritis and Musculoskeletal and Skin Diseases | 7 |
| 21 (tie) | Natural Science Foundation of Shandong Province | 7 |
| 21 (tie) | Tokyo Medical and Dental University | 7 |
| 21 (tie) | Türkiye Bilimsel ve Teknolojik Araştirma Kurumu | 7 |
| 27 (tie) | Capital Medical University | 6 |
| 27 (tie) | Fundação Carlos Chagas Filho de Amparo à Pesquisa do Estado do Rio de Janeiro | 6 |
| 27 (tie) | Karolinska Institutet | 6 |
| 27 (tie) | Ministry of Health and Welfare | 6 |
| 27 (tie) | Ministry of Health, Labour and Welfare | 6 |
| 27 (tie) | National Cancer Institute | 6 |
| 27 (tie) | National Center for Advancing Translational Sciences | 6 |
| 27 (tie) | National Institute of Diabetes and Digestive and Kidney Diseases | 6 |
| 27 (tie) | Natural Science Foundation of Zhejiang Province | 6 |
| 27 (tie) | School of Medicine, Shanghai Jiao Tong University | 6 |
| 27 (tie) | Southern Medical University | 6 |
| 27 (tie) | University of Hong Kong | 6 |
| 39 (tie) | Comisión Nacional de Investigación Científica y Tecnológica | 5 |
| 39 (tie) | Fondo Nacional de Desarrollo Científico y Tecnológico | 5 |
| 39 (tie) | Fundação de Amparo à Pesquisa do Estado de Minas Gerais | 5 |
| 39 (tie) | KU Leuven | 5 |
| 39 (tie) | Ministry of Education | 5 |
| 39 (tie) | National Taiwan University Hospital | 5 |
| 39 (tie) | Natural Science Foundation of Hubei Province | 5 |
| 39 (tie) | Science and Technology Commission of Shanghai Municipality | 5 |
| 39 (tie) | Sichuan Province Science and Technology Support Program | 5 |
| 39 (tie) | Suomen Hammaslääkäriseura Apollonia | 5 |
| 39 (tie) | Türkiye Bilimsel ve Teknolojik Araştırma Kurumu | 5 |
| 39 (tie) | U.S. Department of Veterans Affairs | 5 |
| 39 (tie) | University of Kentucky | 5 |
| 52 (tie) | 8020 Promotion Foundation | 4 |
| 52 (tie) | Beijing Municipal Administration of Hospitals | 4 |
| 52 (tie) | College of Dentistry, University of Kentucky | 4 |
| 52 (tie) | Deanship of Scientific Research, King Saud University | 4 |
| 52 (tie) | Delta Dental Foundation | 4 |
| 52 (tie) | Department of Science and Technology of Sichuan Province | 4 |
| 52 (tie) | European Commission | 4 |
| 52 (tie) | Fundação para a Ciência e a Tecnologia | 4 |
| 52 (tie) | Glaucoma Research Foundation | 4 |
| 52 (tie) | International Team for Implantology | 4 |
| 52 (tie) | Korea Health Industry Development Institute | 4 |
| 52 (tie) | National Health and Medical Research Council | 4 |
| 52 (tie) | National Institute on Aging | 4 |
| 52 (tie) | Natural Science Foundation of Chongqing Municipality | 4 |
| 52 (tie) | Nihon University | 4 |
| 52 (tie) | Ohio State University | 4 |
| 52 (tie) | Research Grants Council, University Grants Committee | 4 |
| 52 (tie) | Research Institute for Humanity and Nature | 4 |
| 52 (tie) | Shandong University | 4 |
| 52 (tie) | U.S. Public Health Service | 4 |
| 52 (tie) | University of Queensland | 4 |
| 52 (tie) | Università degli Studi di Milano | 4 |
| 74 (tie) | Austrian Science Fund | 3 |
| 74 (tie) | Basic and Applied Basic Research Foundation of Guangdong Province | 3 |
| 74 (tie) | Beijing Municipal Administration of Hospitals Clinical Medicine Development of Special Funding Support | 3 |
| 74 (tie) | Biotechnology and Biological Sciences Research Council | 3 |
| 74 (tie) | Center for Outcomes Research and Evaluation, Yale School of Medicine | 3 |
| 74 (tie) | China Scholarship Council | 3 |
| 74 (tie) | Chinese Academy of Sciences | 3 |
| 74 (tie) | Deutsche Forschungsgemeinschaft | 3 |
| 74 (tie) | Fujian Medical University | 3 |
| 74 (tie) | Guangdong Medical Research Foundation | 3 |
| 74 (tie) | Hacettepe Üniversitesi | 3 |
| 74 (tie) | International Association for Dental Research | 3 |
| 74 (tie) | Israel Science Foundation | 3 |
| 74 (tie) | Key Technology Research and Development Program of Shandong | 3 |
| 74 (tie) | Medizinische Universität Wien | 3 |
| 74 (tie) | Ministerio de Ciencia e Innovación | 3 |
| 74 (tie) | Nagasaki University | 3 |
| 74 (tie) | National Health Research Institutes | 3 |
| 74 (tie) | National Institute of Environmental Health Sciences | 3 |
| 74 (tie) | Natural Science Foundation of Beijing Municipality | 3 |
| 74 (tie) | Natural Science Foundation of Hunan Province | 3 |
| 74 (tie) | Natural Science Foundation of Jiangsu Province | 3 |
| 74 (tie) | Natural Science Foundation of Sichuan Province | 3 |
| 74 (tie) | Pfizer | 3 |
| 74 (tie) | School of Dentistry, Nihon University | 3 |
| 74 (tie) | Taipei Medical University Hospital | 3 |
| 74 (tie) | U.S. National Library of Medicine | 3 |

# **SUPPLEMENTARY TABLE 12** Top 100 publications according to AAS by June 1, 2025.

| ***Rank*** | ***AAS*** | ***Author, year*** | ***Title*** |
| --- | --- | --- | --- |
| 1 | 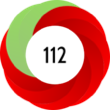 | González et al., 2013 [168] | Antibacterial effects of blackberry extract target periodontopathogens. |
| 2 | 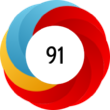 | Reichert et al., 2015 [169] | Use of floss/interdental brushes is associated with lower risk for new cardiovascular events among patients with coronary heart disease. |
| 3 | 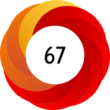 | Yaegaki and Sanada, 1992 [9] | Volatile sulfur compounds in mouth air from clinically healthy subjects and patients with periodontal disease. |
| 4 | 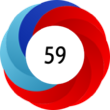 | Laleman et al., 2018 [170] | Influence of tongue brushing and scraping on the oral microflora of periodontitis patients. |
| 5 | 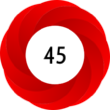 | Kornman and Loesche, 1980 [8] | The subgingival microbial flora during pregnancy. |
| 6 | 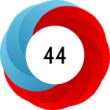 | Maruyama et al., 2022 [171] | Association between serum miRNAs and gingival gene expression in an obese rat model. |
| 7 | 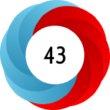 | He et al., 2023 [172] | Demystifying the connection between periodontal disease and chronic kidney disease - An umbrella review. |
| 8 | 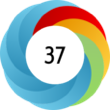 | Long et al., 2017 [106] | Association of oral microbiome with type 2 diabetes risk. |
| 9 | 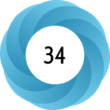 | Schiött and Löe, 1970 [173] | The origin and variation in number of leukocytes in the human saliva. |
| 10 | 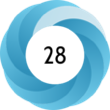 | Jirasek et al., 2024 [174] | Phytocannabinoids and gingival inflammation: Preclinical findings and a placebo-controlled double-blind randomized clinical trial with cannabidiol. |
| 11 | 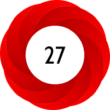 | Castro Dos Santos et al., 2024 [175] | Influence of gender on periodontal outcomes: A retrospective analysis of eight randomized clinical trials. |
| 12 | 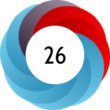 | Bourgeois et al., 2007 [176] | Epidemiology of periodontal status in dentate adults in France, 2002-2003. |
| 13 | 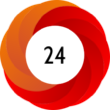 | Wu et al., 2015 [177] | Association of interleukin-1 gene variations with moderate to severe chronic periodontitis in multiple ethnicities. |
| 14 | 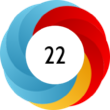 | de Camargo et al., 2019 [178] | Efficacy of toothbrushing procedures performed in intensive care units in reducing the risk of ventilator-associated pneumonia: A systematic review. |
| 15 (tie) | 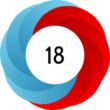 | Lönn et al., 2018 [179] | Lipoprotein modifications by gingipains of Porphyromonas gingivalis. |
| 15 (tie) | 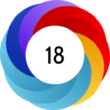 | Ren et al., 2017 [180] | The effectiveness of low-level laser therapy as an adjunct to non-surgical periodontal treatment: a meta-analysis. |
| 17 (tie) | 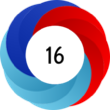 | Nascimento et al., 2024 [101] | Burden of severe periodontitis and edentulism in 2021, with projections up to 2050: The Global Burden of Disease 2021 study. |
| 17 (tie) | 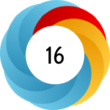 | Sgolastra et al., 2021 [181] | Adjunctive systemic antimicrobials in the treatment of chronic periodontitis: A systematic review and network meta-analysis. |
| 19 (tie) | 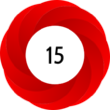 | Ilievski et al., 2017 [182] | Oral application of a periodontal pathogen impacts SerpinE1 expression and pancreatic islet architecture in prediabetes. |
| 19 (tie) | 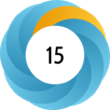 | Khocht et al., 2021 [183] | Cross-sectional comparisons of subgingival microbiome and gingival fluid inflammatory cytokines in periodontally healthy vegetarians versus non-vegetarians. |
| 19 (tie) | 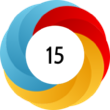 | Wang et al., 2020 [21] | Relationship between periodontal disease and lung cancer: A systematic review and meta-analysis. |
| 22 (tie) | 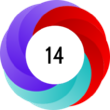 | Dreyer et al., 2018 [10] | Epidemiology and risk factors of peri-implantitis: A systematic review. |
| 22 (tie) | 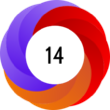 | Theilade et al., 1966 [1] | Experimental gingivitis in man. II. A longitudinal clinical and bacteriological investigation. |
| 22 (tie) | 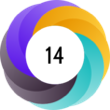 | Keestra et al., 2015 [125] | Non-surgical periodontal therapy with systemic antibiotics in patients with untreated aggressive periodontitis: a systematic review and meta-analysis. |
| 22 (tie) | 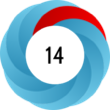 | Nibali et al., 2021 [184] | Periodontal status in children with primary immunodeficiencies. |
| 26 (tie) | 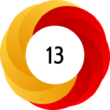 | McKendrick et al., 1968 [185] | A two-year comparison of hand and electric toothbrushes. |
| 26 (tie) | 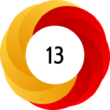 | Cunha et al., 2019 [27] | Periodontal condition and levels of bacteria associated with periodontitis in individuals with bipolar affective disorders: A case-control study. |
| 26 (tie) | 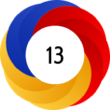 | Zhu et al., 2015 [186] | Multiple tooth loss is associated with vascular cognitive impairment in subjects with acute ischemic stroke. |
| 29 (tie) | 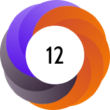 | D. M. Foulkes, 1973 [187] | Some toxicological observations on chlorhexidine. |
| 29 (tie) | 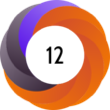 | Henskens et al., 1996 [188] | Protein composition of whole and parotid saliva in healthy and periodontitis subjects. |
| 29 (tie) | 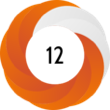 | Rawal et al., 2012 [189] | Effect of cannabidiol on human gingival fibroblast extracellular matrix metabolism: MMP production and activity, and production of fibronectin and transforming growth factor β. |
| 29 (tie) | 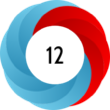 | Yamada et al., 2022 [190] | Regular dental visits, periodontitis, tooth loss, and atherosclerosis: The Ohasama study. |
| 29 (tie) | 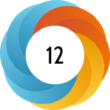 | Danesh-Sani et al., 2017 [115] | Histomorphometric results of different grafting materials and effect of healing time on bone maturation after sinus floor augmentation: a systematic review and meta-analysis. |
| 34 (tie) | 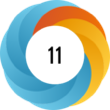 | Novello et al., 2020 [191] | Clinical application of mesenchymal stem cells in periodontal regeneration: A systematic review and meta-analysis. |
| 34 (tie) | 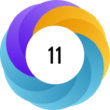 | Zhang et al., 2016 [192] | Clinical effect of azithromycin as an adjunct to non-surgical treatment of chronic periodontitis: a meta-analysis of randomized controlled clinical trials. |
| 34 (tie) | 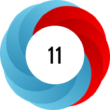 | Aoyama et al., 2018 [36] | Associations among tooth loss, systemic inflammation and antibody titers to periodontal pathogens in Japanese patients with cardiovascular disease. |
| 34 (tie) | 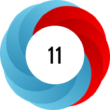 | Menzel et al., 2019 [193] | Activation of vitamin D in the gingival epithelium and its role in gingival inflammation and alveolar bone loss. |
| 34 (tie) | 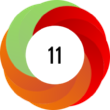 | Balci Yuce et al., 2018 [194] | Investigation of the effect of astaxanthin on alveolar bone loss in experimental periodontitis. |
| 34 (tie) | 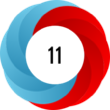 | Pettersson et al., 2017 [39] | Titanium ions form particles that activate and execute interleukin-1β release from lipopolysaccharide-primed macrophages. |
| 40 (tie) | 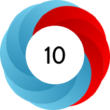 | Mau et al., 2017 [195] | Patients with chronic periodontitis present increased risk for osteoporosis: A population-based cohort study in Taiwan. |
| 40 (tie) | 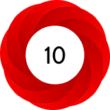 | Kobayashi et al., 2008 [196] | Colonization pattern of periodontal bacteria in Japanese children and their mothers. |
| 40 (tie) | 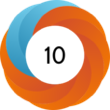 | Chaparro et al., 2013 [197] | Increased inflammatory biomarkers in early pregnancy is associated with the development of pre-eclampsia in patients with periodontitis: a case control study. |
| 40 (tie) | 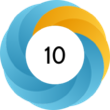 | Castro Dos Santos et al., 2022 [198] | Does the use of omega-3 fatty acids as an adjunct to non-surgical periodontal therapy provide additional benefits in the treatment of periodontitis? A systematic review and meta-analysis. |
| 40 (tie) | 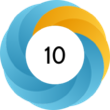 | Chisini et al., 2019 [199] | Is the use of Cannabis associated with periodontitis? A systematic review and meta-analysis. |
| 45 (tie) | 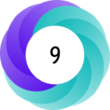 | Imery et al., 1994 [200] | Recommended revisions to American Dental Association guidelines for acceptance of chemotherapeutic products for gingivitis control. Report of the Task Force on Design and Analysis in Dental and Oral Research to the Council on Therapeutics of the American Dental Association. |
| 45 (tie) | 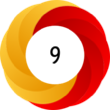 | Yamamoto et al., 1999 [201] | The structure and function of the cemento-dentinal junction in human teeth. |
| 45 (tie) | 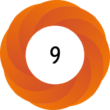 | Yu et al., 1993 [202] | Chemically-modified tetracycline normalizes collagen metabolism in diabetic rats: a dose-response study. |
| 45 (tie) | 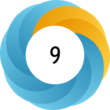 | Akram et al., 2018 [203] | Locally delivered metformin as adjunct to scaling and root planing in the treatment of periodontal defects: A systematic review and meta-analysis. |
| 45 (tie) | 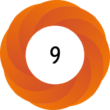 | Bertrand-Duchesne et al., 2010 [204] | Epidermal growth factor released from platelet-rich plasma promotes endothelial cell proliferation in vitro. |
| 45 (tie) | 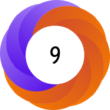 | Lindhe et al., 1975 [13] | Plaque induced periodontal disease in beagle dogs. A 4-year clinical, roentgenographical and histometrical study. |
| 45 (tie) | 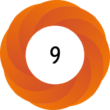 | Bender et al., 2006 [205] | Novel rinse assay for the quantification of oral neutrophils and the monitoring of chronic periodontal disease. |
| 45 (tie) | 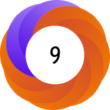 | Smith et al., 1991 [206] | Inhibition of intergeneric coaggregation among oral bacteria by cetylpyridinium chloride, chlorhexidine digluconate and octenidine dihydrochloride. |
| 45 (tie) | 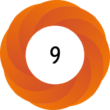 | Neiders et al., 1989 [70] | Heterogeneity of virulence among strains of Bacteroides gingivalis. |
| 45 (tie) | 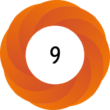 | Tani-Ishii et al., 1997 [207] | Osteopontin antisense deoxyoligonucleotides inhibit bone resorption by mouse osteoclasts in vitro. |
| 45 (tie) | 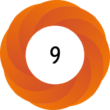 | Tang-Larsen et al., 1995 [208] | Competition for peptides and amino acids among periodontal bacteria. |
| 45 (tie) | 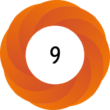 | Pirk et al., 1974 [209] | Effect of analogues of steroid and thyroxine hormones on wound healing in hamsters. |
| 45 (tie) | 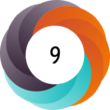 | Saxton and van der Ouderaa, 1989 [92] | The effect of a dentifrice containing zinc citrate and Triclosan on developing gingivitis. |
| 45 (tie) | 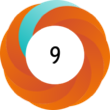 | Golub et al., 1985 [50] | Further evidence that tetracyclines inhibit collagenase activity in human crevicular fluid and from other mammalian sources. |
| 45 (tie) | 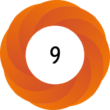 | Friedman and Golomb, 1982 [210] | New sustained release dosage form of chlorhexidine for dental use. I. Development and kinetics of release. |
| 45 (tie) | 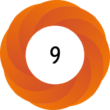 | Rojo-Botello et al., 2012 [211] | Expression of toll-like receptors 2, 4 and 9 is increased in gingival tissue from patients with type 2 diabetes and chronic periodontitis. |
| 45 (tie) | 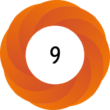 | Liu et al., 2009 [212] | In vivo determination of multiple indices of periodontal inflammation by optical spectroscopy. |
| 45 (tie) | 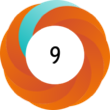 | Golub et al., 1990 [47] | Low-dose doxycycline therapy: effect on gingival and crevicular fluid collagenase activity in humans. |
| 45 (tie) | 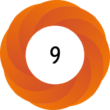 | Suido et al., 1987 [213] | Characterization of N-CBz-glycyl-glycyl-arginyl peptidase and glycyl-prolyl peptidase of Bacteroides gingivalis. |
| 45 (tie) | 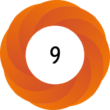 | Curtis et al., 1999 [48] | Molecular genetics and nomenclature of proteases of Porphyromonas gingivalis. |
| 45 (tie) | 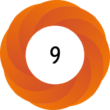 | Huynh et al., 2015 [214] | Gingival crevicular fluid proteomes in health, gingivitis and chronic periodontitis. |
| 45 (tie) | 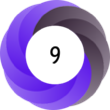 | Beck et al., 1997 [215] | A 5-year study of attachment loss and tooth loss in community-dwelling older adults. |
| 45 (tie) | 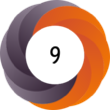 | Nagatomo et al., 2006 [14] | Stem cell properties of human periodontal ligament cells. |
| 45 (tie) | 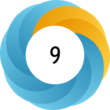 | Hagenfeld et al., 2019 [216] | No differences in microbiome changes between anti-adhesive and antibacterial ingredients in toothpastes during periodontal therapy. |
| 45 (tie) | 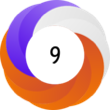 | Löe and Schiøtt, 1970 [3] | The effect of mouthrinses and topical application of chlorhexidine on the development of dental plaque and gingivitis in man. |
| 45 (tie) | 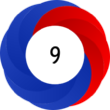 | Mizutani et al., 2015 [217] | Relationship between xerostomia and gingival condition in young adults. |
| 45 (tie) | 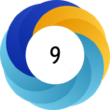 | Fernandez et al., 2017 [218] | A reproducible microcosm biofilm model of subgingival microbial communities. |
| 45 (tie) | 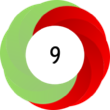 | Li et al., 2021 [136] | Curcumin: A review of experimental studies and mechanisms related to periodontitis treatment. |
| 45 (tie) | 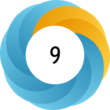 | Calciolari et al., 2022 [167] | The efficacy of adjunctive periodontal therapies during supportive periodontal care in patients with residual pockets. A systematic review and meta-analysis. |
| 45 (tie) | 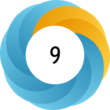 | Rademacher et al., 2019 [219] | Qualitative and quantitative differences in the subgingival microbiome of the restored and unrestored teeth. |
| 45 (tie) | 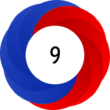 | Noronha Oliveira et al., 2018 [54] | Can degradation products released from dental implants affect peri-implant tissues? |
| 45 (tie) | 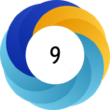 | Alkaya et al., 2017 [220] | Clinical effects of probiotics containing Bacillus species on gingivitis: a pilot randomized controlled trial. |
| 45 (tie) | 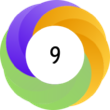 | Tsai et al., 2008 [221] | The levels of volatile sulfur compounds in mouth air from patients with chronic periodontitis. |
| 78 (tie) | 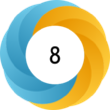 | Park et al., 2024 [222] | Differences in maternal subgingival microbiome between preterm and term births: The MOHEPI study. |
| 78 (tie) | 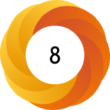 | Takemoto et al., 1993 [223] | Purification of arginine-sensitive hemagglutinin from Fusobacterium nucleatum and its role in coaggregation. |
| 78 (tie) | 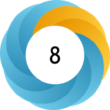 | Brun et al., 2021 [224] | Oral microbiota and atherothrombotic carotid plaque vulnerability in periodontitis patients. A cross-sectional study. |
| 78 (tie) | 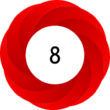 | Khosravisamani et al., 2014 [225] | Effect of the menstrual cycle on inflammatory cytokines in the periodontium. |
| 78 (tie) | 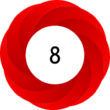 | Heijl et al., 1980 [226] | Periodontal disease in gnotobiotic rats. |
| 78 (tie) | 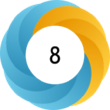 | Li et al., 2018 [227] | Integrated analysis of long noncoding RNA-associated competing endogenous RNA network in periodontitis. |
| 78 (tie) | 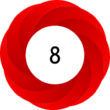 | Yang et al., 2017 [228] | Involvement of CD147 in alveolar bone remodeling and soft tissue degradation in experimental periodontitis. |
| 78 (tie) | 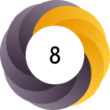 | Payne et al., 1975 [61] | Histopathologic features of the initial and early stages of experimental gingivitis in man. |
| 78 (tie) | 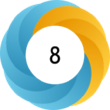 | de Oliveira et al., 2022 [229] | Dysbiotic relationship between arthritis and the oral-gut microbiome. A critical review. |
| 78 (tie) | 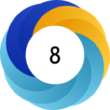 | Ambrósio et al., 2018 [230] | Does the adjunctive use of statins provide additional benefits to nonsurgical periodontal treatment? A systematic review and meta-analysis. |
| 78 (tie) | 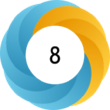 | Seymour et al., 2017 [231] | Influence of a triclosan toothpaste on periodontopathic bacteria and periodontitis progression in cardiovascular patients: a randomized controlled trial. |
| 78 (tie) | 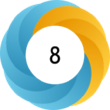 | Faggion et al., 2016 [232] | An overview of systematic reviews on the effectiveness of periodontal treatment to improve glycaemic control. |
| 78 (tie) | 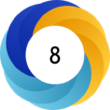 | Hamasaki et al., 2017 [233] | Periodontal disease and percentage of calories from fat using national data. |
| 78 (tie) | 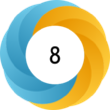 | Jin et al., 2015 [132] | Isolation and characterization of human mesenchymal stem cells from gingival connective tissue. |
| 78 (tie) | 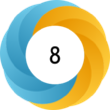 | Sato et al., 2018 [234] | An orally administered oral pathobiont and commensal have comparable and innocuous systemic effects in germ-free mice. |
| 78 (tie) | 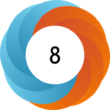 | Moritani et al., 2018 [235] | Spheroid culture enhances osteogenic potential of periodontal ligament mesenchymal stem cells. |
| 78 (tie) | 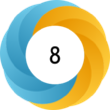 | Fang et al., 2016 [236] | Comparison of full-mouth disinfection and quadrant-wise scaling in the treatment of adult chronic periodontitis: a systematic review and meta-analysis. |
| 78 (tie) | 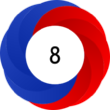 | Lombardo Bedran et al., 2014 [237] | Green tea extract and its major constituent, epigallocatechin-3-gallate, induce epithelial beta-defensin secretion and prevent beta-defensin degradation by Porphyromonas gingivalis. |
| 78 (tie) | 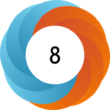 | Casarin et al., 2013 [53] | Subgingival biodiversity in subjects with uncontrolled type-2 diabetes and chronic periodontitis. |
| 97 (tie) | 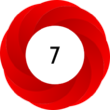 | Oh et al., 2022 [238] | Transepithelial channels for leukocytes in the junctional epithelium. |
| 97 (tie) | 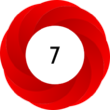 | J. Caton, 1992 [239] | Biological and measurement issues critical to design of gingivitis trials. |
| 97 (tie) | 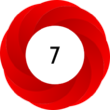 | Hong et al., 2016 [240] | Anti-inflammatory and anti-osteoclastogenic effects of zinc finger protein A20 overexpression in human periodontal ligament cells. |
| 97 (tie) | 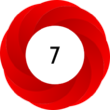 | Shen et al., 2024 [241] | Abnormal amyloid precursor protein processing in periodontal tissue in a murine model of periodontitis induced by Porphyromonas gingivalis. |

# **SUPPLEMENTARY FIGURE 1** Co-authorship analysis of authors with ≥10 publications by June 1, 2025 presented with **(A)** network and **(B)** density visualization.

# **SUPPLEMENTARY FIGURE 2** International collaboration network between countries with ≥ 5 publications in *JPR* from 1966 to June 1, 2025. The width of the connecting line presents the number of publications.

# **SUPPLEMENTARY FIGURE 3** Geographical distribution of Altmetric mentions in **(A)** X, **(B)** Facebook, and **(C)** news by by June 1, 2025.

# **REFERENCES**

1. E. Theilade, W. H. Wright, S. B. Jensen, and H. Löe, “Experimental gingivitis in man. II. A longitudinal clinical and bacteriological investigation,” *J Periodontal Res 1* (1966): 1–13, <https://doi.org/10.1111/j.1600-0765.1966.tb01842.x>.

2. R. C. Page, “The role of inflammatory mediators in the pathogenesis of periodontal disease,” *J Periodontal Res 26* (1991): 230–242, <https://doi.org/10.1111/j.1600-0765.1991.tb01649.x>.

3. H. Löe and C. R. Schiott, “The effect of mouthrinses and topical application of chlorhexidine on the development of dental plaque and gingivitis in man,” *J Periodontal Res 5* (1970): 79–83, <https://doi.org/10.1111/j.1600-0765.1970.tb00696.x>.

4. H. Birkedal-Hansen, “Role of cytokines and inflammatory mediators in tissue destruction,” *J Periodontal Res 28* (1993): 500–510, <https://doi.org/10.1111/j.1600-0765.1993.tb02113.x>.

5. S. Offenbacher, B. M. Odle, and T. E. Van Dyke, “The use of crevicular fluid prostaglandin E2 levels as a predictor of periodontal attachment loss,” *J Periodontal Res 21* (1986): 101–112, <https://doi.org/10.1111/j.1600-0765.1986.tb01443.x>.

6. L. M. Golub, H. M. Lee, G. Lehrer, A. Nemiroff, T. F. McNamara, R. Kaplan, et al., “Minocycline reduces gingival collagenolytic activity during diabetes. Preliminary observations and a proposed new mechanism of action,” *J Periodontal Res 18* (1983): 516–526, <https://doi.org/10.1111/j.1600-0765.1983.tb00388.x>.

7. M. P. Masada, R. Persson, J. S. Kenney, S. W. Lee, R. C. Page, and A. C. Allison, “Measurement of interleukin-1 alpha and -1 beta in gingival crevicular fluid: implications for the pathogenesis of periodontal disease,” *J Periodontal Res 25* (1990): 156–163, <https://doi.org/10.1111/j.1600-0765.1990.tb01038.x>.

8. K. S. Kornman and W. J. Loesche, “The subgingival microbial flora during pregnancy,” *J Periodontal Res 15* (1980): 111–122, <https://doi.org/10.1111/j.1600-0765.1980.tb00265.x>.

9. K. Yaegaki and K. Sanada, “Volatile sulfur compounds in mouth air from clinically healthy subjects and patients with periodontal disease,” *Journal of Periodontal Research 27* (1992): 233–238, <https://doi.org/https://doi.org/10.1111/j.1600-0765.1992.tb01673.x>.

10. H. Dreyer, J. Grischke, C. Tiede, J. Eberhard, A. Schweitzer, S. E. Toikkanen, et al., “Epidemiology and risk factors of peri-implantitis: A systematic review,” *J Periodontal Res 53* (2018): 657–681, <https://doi.org/10.1111/jre.12562>.

11. M. G. Newman and S. S. Socransky, “Predominant cultivable microbiota in periodontosis,” *J Periodontal Res 12* (1977): 120–128, <https://doi.org/10.1111/j.1600-0765.1977.tb00114.x>.

12. W. E. Moore, “Microbiology of periodontal disease,” *J Periodontal Res 22* (1987): 335–341, <https://doi.org/10.1111/j.1600-0765.1987.tb01595.x>.

13. J. Lindhe, S. E. Hamp, and H. Löe, “Plaque induced periodontal disease in beagle dogs. A 4-year clinical, roentgenographical and histometrical study,” *J Periodontal Res 10* (1975): 243–255, <https://doi.org/10.1111/j.1600-0765.1975.tb00031.x>.

14. K. Nagatomo, M. Komaki, I. Sekiya, Y. Sakaguchi, K. Noguchi, S. Oda, et al., “Stem cell properties of human periodontal ligament cells,” *J Periodontal Res 41* (2006): 303–310, <https://doi.org/10.1111/j.1600-0765.2006.00870.x>.

15. L. Glavind and H. Löe, “Errors in the clinical assessment of periodontal destruction,” *J Periodontal Res 2* (1967): 180–184, <https://doi.org/10.1111/j.1600-0765.1967.tb01887.x>.

16. C. A. McCulloch and S. Bordin, “Role of fibroblast subpopulations in periodontal physiology and pathology,” *J Periodontal Res 26* (1991): 144–154, <https://doi.org/10.1111/j.1600-0765.1991.tb01638.x>.

17. S. S. Socransky, A. D. Manganiello, D. Propas, V. Oram, and J. van Houte, “Bacteriological studies of developing supragingival dental plaque,” *J Periodontal Res 12* (1977): 90–106, <https://doi.org/10.1111/j.1600-0765.1977.tb00112.x>.

18. L. M. Golub, N. Ramamurthy, T. F. McNamara, B. Gomes, M. Wolff, A. Casino, et al., “Tetracyclines inhibit tissue collagenase activity. A new mechanism in the treatment of periodontal disease,” *J Periodontal Res 19* (1984): 651–655, <https://doi.org/10.1111/j.1600-0765.1984.tb01334.x>.

19. T. Crotti, M. D. Smith, R. Hirsch, S. Soukoulis, H. Weedon, M. Capone, et al., “Receptor activator NF kappaB ligand (RANKL) and osteoprotegerin (OPG) protein expression in periodontitis,” *J Periodontal Res 38* (2003): 380–387, <https://doi.org/10.1034/j.1600-0765.2003.00615.x>.

20. T. Mousquès, M. A. Listgarten, and R. W. Phillips, “Effect of scaling and root planing on the composition of the human subgingival microbial flora,” *J Periodontal Res 15* (1980): 144–151, <https://doi.org/10.1111/j.1600-0765.1980.tb00268.x>.

21. J. Wang, X. Yang, X. Zou, Y. Zhang, J. Wang, and Y. Wang, “Relationship between periodontal disease and lung cancer: A systematic review and meta-analysis,” *J Periodontal Res 55* (2020): 581–593, <https://doi.org/10.1111/jre.12772>.

22. T. Karring, N. P. Lang, and H. Löe, “The role of gingival connective tissue in determining epithelial differentiation,” *J Periodontal Res 10* (1975): 1–11, <https://doi.org/10.1111/j.1600-0765.1975.tb00001.x>.

23. J. Lindhe, S. Hamp, and H. Löe, “Experimental periodontitis in the beagle dog,” *J Periodontal Res 8* (1973): 1–10, <https://doi.org/10.1111/j.1600-0765.1973.tb00735.x>.

24. P. M. Camargo, V. Lekovic, M. Weinlaender, N. Vasilic, M. Madzarevic, and E. B. Kenney, “Platelet-rich plasma and bovine porous bone mineral combined with guided tissue regeneration in the treatment of intrabony defects in humans,” *J Periodontal Res 37* (2002): 300–306, <https://doi.org/10.1034/j.1600-0765.2002.01001.x>.

25. M. C. Ferreira, A. C. Dias-Pereira, L. S. Branco-de-Almeida, C. C. Martins, and S. M. Paiva, “Impact of periodontal disease on quality of life: a systematic review,” *J Periodontal Res 52* (2017): 651–665, <https://doi.org/10.1111/jre.12436>.

26. H. Löe, C. R. Schiött, G. Karring, and T. Karring, “Two years oral use of chlorhexidine in man. I. General design and clinical effects,” *J Periodontal Res 11* (1976): 135–144, <https://doi.org/10.1111/j.1600-0765.1976.tb00061.x>.

27. F. A. Cunha, L. O. M. Cota, S. C. Cortelli, T. B. Miranda, F. S. Neves, J. R. Cortelli, et al., “Periodontal condition and levels of bacteria associated with periodontitis in individuals with bipolar affective disorders: A case-control study,” *J Periodontal Res 54* (2019): 63–72, <https://doi.org/10.1111/jre.12605>.

28. G. J. Seymour, E. Gemmell, R. A. Reinhardt, J. Eastcott, and M. A. Taubman, “Immunopathogenesis of chronic inflammatory periodontal disease: cellular and molecular mechanisms,” *J Periodontal Res 28* (1993): 478–486, <https://doi.org/10.1111/j.1600-0765.1993.tb02108.x>.

29. P. Apse, R. P. Ellen, C. M. Overall, and G. A. Zarb, “Microbiota and crevicular fluid collagenase activity in the osseointegrated dental implant sulcus: a comparison of sites in edentulous and partially edentulous patients,” *J Periodontal Res 24* (1989): 96–105, <https://doi.org/10.1111/j.1600-0765.1989.tb00863.x>.

30. G. P. Garlet, W. Martins, Jr., B. R. Ferreira, C. M. Milanezi, and J. S. Silva, “Patterns of chemokines and chemokine receptors expression in different forms of human periodontal disease,” *J Periodontal Res 38* (2003): 210–217, <https://doi.org/10.1034/j.1600-0765.2003.02012.x>.

31. B. A. Dale, J. R. Kimball, S. Krisanaprakornkit, F. Roberts, M. Robinovitch, R. O'Neal, et al., “Localized antimicrobial peptide expression in human gingiva,” *J Periodontal Res 36* (2001): 285–294, <https://doi.org/10.1034/j.1600-0765.2001.360503.x>.

32. P. Gjermo, K. Lyche Baastad, and G. Rölla, “The plaque-inhibiting capacity of 11 antibacterial compounds,” *Journal of Periodontal Research 5* (1970): 102–109, <https://doi.org/https://doi.org/10.1111/j.1600-0765.1970.tb00700.x>.

33. G. Rölla, H. Löe, and C. R. Schiott, “The affinity of chlorhexidine for hydroxyapatite and salivary mucins,” *J Periodontal Res 5* (1970): 90–95, <https://doi.org/10.1111/j.1600-0765.1970.tb00698.x>.

34. A. M. Schmidt, E. Weidman, E. Lalla, S. D. Yan, O. Hori, R. Cao, et al., “Advanced glycation endproducts (AGEs) induce oxidant stress in the gingiva: a potential mechanism underlying accelerated periodontal disease associated with diabetes,” *J Periodontal Res 31* (1996): 508–515, <https://doi.org/10.1111/j.1600-0765.1996.tb01417.x>.

35. C. C. Tsai, H. S. Chen, S. L. Chen, Y. P. Ho, K. Y. Ho, Y. M. Wu, et al., “Lipid peroxidation: a possible role in the induction and progression of chronic periodontitis,” *J Periodontal Res 40* (2005): 378–384, <https://doi.org/10.1111/j.1600-0765.2005.00818.x>.

36. N. Aoyama, J. I. Suzuki, N. Kobayashi, T. Hanatani, N. Ashigaki, A. Yoshida, et al., “Associations among tooth loss, systemic inflammation and antibody titers to periodontal pathogens in Japanese patients with cardiovascular disease,” *J Periodontal Res 53* (2018): 117–122, <https://doi.org/10.1111/jre.12494>.

37. E. D. Savitt and S. S. Socransky, “Distribution of certain subgingival microbial species in selected periodontal conditions,” *J Periodontal Res 19* (1984): 111–123, <https://doi.org/10.1111/j.1600-0765.1984.tb00800.x>.

38. B. F. Mackler, K. B. Frostad, P. B. Robertson, and B. M. Levy, “Immunoglobulin bearing lymphocytes and plasma cells in human periodontal disease,” *J Periodontal Res 12* (1977): 37–45, <https://doi.org/10.1111/j.1600-0765.1977.tb00107.x>.

39. M. Pettersson, P. Kelk, G. N. Belibasakis, D. Bylund, M. Molin Thorén, and A. Johansson, “Titanium ions form particles that activate and execute interleukin-1β release from lipopolysaccharide-primed macrophages,” *J Periodontal Res 52* (2017): 21–32, <https://doi.org/10.1111/jre.12364>.

40. R. T. Cole, M. Crigger, G. Bogle, J. Egelberg, and K. A. Selvig, “Connective tissue regeneration to periodontally diseased teeth. A histological study,” *J Periodontal Res 15* (1980): 1–9, <https://doi.org/10.1111/j.1600-0765.1980.tb00256.x>.

41. J. Slots, “Selection of antimicrobial agents in periodontal therapy,” *J Periodontal Res 37* (2002): 389–398, <https://doi.org/10.1034/j.1600-0765.2002.00004.x>.

42. A. Sculean, N. Donos, P. Windisch, M. Brecx, I. Gera, E. Reich, et al., “Healing of human intrabony defects following treatment with enamel matrix proteins or guided tissue regeneration,” *J Periodontal Res 34* (1999): 310–322, <https://doi.org/10.1111/j.1600-0765.1999.tb02259.x>.

43. S. Murakami, S. Takayama, K. Ikezawa, Y. Shimabukuro, M. Kitamura, T. Nozaki, et al., “Regeneration of periodontal tissues by basic fibroblast growth factor,” *J Periodontal Res 34* (1999): 425–430, <https://doi.org/10.1111/j.1600-0765.1999.tb02277.x>.

44. W. S. Lavine, E. G. Maderazo, J. Stolman, P. A. Ward, R. B. Cogen, I. Greenblatt, et al., “Impaired neutrophil chemotaxis in patients with juvenile and rapidly progressing periodontitis,” *J Periodontal Res 14* (1979): 10–19, <https://doi.org/10.1111/j.1600-0765.1979.tb00213.x>.

45. R. Attström, “Presence of leukocytes in crevices of healthy and chronically inflamed gingivae,” *J Periodontal Res 5* (1970): 42–47, <https://doi.org/10.1111/j.1600-0765.1970.tb01836.x>.

46. S. Murakami, S. Takayama, M. Kitamura, Y. Shimabukuro, K. Yanagi, K. Ikezawa, et al., “Recombinant human basic fibroblast growth factor (bFGF) stimulates periodontal regeneration in class II furcation defects created in beagle dogs,” *J Periodontal Res 38* (2003): 97–103, <https://doi.org/10.1034/j.1600-0765.2003.00640.x>.

47. L. M. Golub, S. Ciancio, N. S. Ramamamurthy, M. Leung, and T. F. McNamara, “Low-dose doxycycline therapy: effect on gingival and crevicular fluid collagenase activity in humans,” *J Periodontal Res 25* (1990): 321–330, <https://doi.org/10.1111/j.1600-0765.1990.tb00923.x>.

48. M. A. Curtis, H. K. Kuramitsu, M. Lantz, F. L. Macrina, K. Nakayama, J. Potempa, et al., “Molecular genetics and nomenclature of proteases of Porphyromonas gingivalis,” *J Periodontal Res 34* (1999): 464–472, <https://doi.org/10.1111/j.1600-0765.1999.tb02282.x>.

49. S. Rovin, E. R. Costich, and H. A. Gordon, “The influence of bacteria and irritation in the initiation of periodontal disease in germfree and conventional rats,” *J Periodontal Res 1* (1966): 193–204, <https://doi.org/10.1111/j.1600-0765.1966.tb01860.x>.

50. L. M. Golub, M. Wolff, H. M. Lee, T. F. McNamara, N. S. Ramamurthy, J. Zambon, et al., “Further evidence that tetracyclines inhibit collagenase activity in human crevicular fluid and from other mammalian sources,” *J Periodontal Res 20* (1985): 12–23, <https://doi.org/10.1111/j.1600-0765.1985.tb00405.x>.

51. S. Offenbacher, B. M. Odle, R. C. Gray, and T. E. Van Dyke, “Crevicular fluid prostaglandin E levels as a measure of the periodontal disease status of adult and juvenile periodontitis patients,” *J Periodontal Res 19* (1984): 1–13, <https://doi.org/10.1111/j.1600-0765.1984.tb01190.x>.

52. S. S. Socransky and A. D. Haffajee, “Microbial mechanisms in the pathogenesis of destructive periodontal diseases: a critical assessment,” *J Periodontal Res 26* (1991): 195–212, <https://doi.org/10.1111/j.1600-0765.1991.tb01646.x>.

53. R. C. Casarin, A. Barbagallo, T. Meulman, V. R. Santos, E. A. Sallum, F. H. Nociti, et al., “Subgingival biodiversity in subjects with uncontrolled type-2 diabetes and chronic periodontitis,” *J Periodontal Res 48* (2013): 30–36, <https://doi.org/10.1111/j.1600-0765.2012.01498.x>.

54. M. Noronha Oliveira, W. V. H. Schunemann, M. T. Mathew, B. Henriques, R. S. Magini, W. Teughels, et al., “Can degradation products released from dental implants affect peri-implant tissues?,” *J Periodontal Res 53* (2018): 1–11, <https://doi.org/10.1111/jre.12479>.

55. R. B. Rutherford, C. E. Niekrash, J. E. Kennedy, and M. F. Charette, “Platelet-derived and insulin-like growth factors stimulate regeneration of periodontal attachment in monkeys,” *J Periodontal Res 27* (1992): 285–290, <https://doi.org/10.1111/j.1600-0765.1992.tb01679.x>.

56. P. A. Heasman, J. G. Collins, and S. Offenbacher, “Changes in crevicular fluid levels of interleukin-1 beta, leukotriene B4, prostaglandin E2, thromboxane B2 and tumour necrosis factor alpha in experimental gingivitis in humans,” *J Periodontal Res 28* (1993): 241–247, <https://doi.org/10.1111/j.1600-0765.1993.tb02090.x>.

57. W. V. Giannobile, R. A. Hernandez, R. D. Finkelman, S. Ryan, C. P. Kiritsy, M. D'Andrea, et al., “Comparative effects of platelet-derived growth factor-BB and insulin-like growth factor-I, individually and in combination, on periodontal regeneration in Macaca fascicularis,” *J Periodontal Res 31* (1996): 301–312, <https://doi.org/10.1111/j.1600-0765.1996.tb00497.x>.

58. N. Lang and M. C. Brecx, “Chlorhexidine digluconate–an agent for chemical plaque control and prevention of gingival inflammation,” *Journal of Periodontal Research 21* (1986): 74–89, <https://doi.org/https://doi.org/10.1111/j.1600-0765.1986.tb01517.x>.

59. F. D'Aiuto, D. Ready, and M. S. Tonetti, “Periodontal disease and C-reactive protein-associated cardiovascular risk,” *J Periodontal Res 39* (2004): 236–241, <https://doi.org/10.1111/j.1600-0765.2004.00731.x>.

60. J. Hönig, C. Rordorf-Adam, C. Siegmund, W. Wiedemann, and F. Erard, “Increased interleukin-1 beta (IL-1 beta) concentration in gingival tissue from periodontitis patients,” *J Periodontal Res 24* (1989): 362–367, <https://doi.org/10.1111/j.1600-0765.1989.tb00883.x>.

61. W. A. Payne, R. C. Page, A. L. Ogilvie, and W. B. Hall, “Histopathologic features of the initial and early stages of experimental gingivitis in man,” *J Periodontal Res 10* (1975): 51–64, <https://doi.org/10.1111/j.1600-0765.1975.tb00008.x>.

62. T. R. Gould, A. H. Melcher, and D. M. Brunette, “Migration and division of progenitor cell populations in periodontal ligament after wounding,” *J Periodontal Res 15* (1980): 20–42, <https://doi.org/10.1111/j.1600-0765.1980.tb00258.x>.

63. P. Mäntylä, M. Stenman, D. F. Kinane, S. Tikanoja, H. Luoto, T. Salo, et al., “Gingival crevicular fluid collagenase-2 (MMP-8) test stick for chair-side monitoring of periodontitis,” *J Periodontal Res 38* (2003): 436–439, <https://doi.org/10.1034/j.1600-0765.2003.00677.x>.

64. T. Saito, Y. Shimazaki, Y. Kiyohara, I. Kato, M. Kubo, M. Iida, et al., “Relationship between obesity, glucose tolerance, and periodontal disease in Japanese women: the Hisayama study,” *J Periodontal Res 40* (2005): 346–353, <https://doi.org/10.1111/j.1600-0765.2005.00813.x>.

65. H. V. Jordan, P. H. Keyes, and S. Bellack, “Periodontal lesions in hamsters and gnotobiotic rats infected with actinomyces of human origin,” *J Periodontal Res 7* (1972): 21–28, <https://doi.org/10.1111/j.1600-0765.1972.tb00627.x>.

66. V. Baelum, O. Fejerskov, and T. Karring, “Oral hygiene, gingivitis and periodontal breakdown in adult Tanzanians,” *J Periodontal Res 21* (1986): 221–232, <https://doi.org/10.1111/j.1600-0765.1986.tb01454.x>.

67. S. Pitaru, C. A. McCulloch, and S. A. Narayanan, “Cellular origins and differentiation control mechanisms during periodontal development and wound healing,” *J Periodontal Res 29* (1994): 81–94, <https://doi.org/10.1111/j.1600-0765.1994.tb01095.x>.

68. T. Ara, K. Kurata, K. Hirai, T. Uchihashi, T. Uematsu, Y. Imamura, et al., “Human gingival fibroblasts are critical in sustaining inflammation in periodontal disease,” *J Periodontal Res 44* (2009): 21–27, <https://doi.org/10.1111/j.1600-0765.2007.01041.x>.

69. U. E. Ruttimann, R. L. Webber, and E. Schmidt, “A robust digital method for film contrast correction in subtraction radiography,” *J Periodontal Res 21* (1986): 486–495, <https://doi.org/10.1111/j.1600-0765.1986.tb01484.x>.

70. M. E. Neiders, P. B. Chen, H. Suido, H. S. Reynolds, J. J. Zambon, M. Shlossman, et al., “Heterogeneity of virulence among strains of Bacteroides gingivalis,” *J Periodontal Res 24* (1989): 192–198, <https://doi.org/10.1111/j.1600-0765.1989.tb02005.x>.

71. D. White and D. Mayrand, “Association of oral Bacteroides with gingivitis and adult periodontitis,” *J Periodontal Res 16* (1981): 259–265, <https://doi.org/10.1111/j.1600-0765.1981.tb00974.x>.

72. M. Hirasawa, K. Takada, M. Makimura, and S. Otake, “Improvement of periodontal status by green tea catechin using a local delivery system: a clinical pilot study,” *J Periodontal Res 37* (2002): 433–438, <https://doi.org/10.1034/j.1600-0765.2002.01640.x>.

73. G. J. Seymour and J. S. Greenspan, “The phenotypic characterization of lymphocyte subpopulations in established human periodontal disease,” *J Periodontal Res 14* (1979): 39–46, <https://doi.org/10.1111/j.1600-0765.1979.tb00216.x>.

74. T. Sorsa, V. J. Uitto, K. Suomalainen, M. Vauhkonen, and S. Lindy, “Comparison of interstitial collagenases from human gingiva, sulcular fluid and polymorphonuclear leukocytes,” *J Periodontal Res 23* (1988): 386–393, <https://doi.org/10.1111/j.1600-0765.1988.tb01618.x>.

75. J. S. Garrett, M. Crigger, and J. Egelberg, “Effects of citric acid on diseased root surfaces,” *J Periodontal Res 13* (1978): 155–163, <https://doi.org/10.1111/j.1600-0765.1978.tb00164.x>.

76. C. R. Fontana, A. D. Abernethy, S. Som, K. Ruggiero, S. Doucette, R. C. Marcantonio, et al., “The antibacterial effect of photodynamic therapy in dental plaque-derived biofilms,” *J Periodontal Res 44* (2009): 751–759, <https://doi.org/10.1111/j.1600-0765.2008.01187.x>.

77. C. R. Schiott, H. Löe, S. B. Jensen, M. Kilian, R. M. Davies, and K. Glavind, “The effect of chlorhexidine mouthrinses on the human oral flora,” *J Periodontal Res 5* (1970): 84–89, <https://doi.org/10.1111/j.1600-0765.1970.tb00697.x>.

78. T. de Jong, A. D. Bakker, V. Everts, and T. H. Smit, “The intricate anatomy of the periodontal ligament and its development: Lessons for periodontal regeneration,” *J Periodontal Res 52* (2017): 965–974, <https://doi.org/10.1111/jre.12477>.

79. E. B. Kenney, J. H. Kraal, S. R. Saxe, and J. Jones, “The effect of cigarette smoke on human oral polymorphonuclear leukocytes,” *J Periodontal Res 12* (1977): 227–234, <https://doi.org/10.1111/j.1600-0765.1977.tb00126.x>.

80. T. Karring, E. Ostergaard, and H. Löe, “Conservation of tissue specificity after heterotopic transplantation of gingiva and alveolar mucosa,” *J Periodontal Res 6* (1971): 282–293, <https://doi.org/10.1111/j.1600-0765.1971.tb00619.x>.

81. S. Nyman, J. Gottlow, J. Lindhe, T. Karring, and J. Wennstrom, “New attachment formation by guided tissue regeneration,” *J Periodontal Res 22* (1987): 252–254, <https://doi.org/10.1111/j.1600-0765.1987.tb01581.x>.

82. N. Nojima, M. Kobayashi, M. Shionome, N. Takahashi, T. Suda, and K. Hasegawa, “Fibroblastic cells derived from bovine periodontal ligaments have the phenotypes of osteoblasts,” *J Periodontal Res 25* (1990): 179–185, <https://doi.org/10.1111/j.1600-0765.1990.tb01041.x>.

83. B. Zhang, Y. Yang, J. Yi, Z. Zhao, and R. Ye, “Hyperglycemia modulates M1/M2 macrophage polarization via reactive oxygen species overproduction in ligature-induced periodontitis,” *J Periodontal Res 56* (2021): 991–1005, <https://doi.org/10.1111/jre.12912>.

84. A. C. Tanner, S. S. Socransky, and J. M. Goodson, “Microbiota of periodontal pockets losing crestal alveolar bone,” *J Periodontal Res 19* (1984): 279–291, <https://doi.org/10.1111/j.1600-0765.1984.tb00819.x>.

85. G. E. Salvi, C. E. Brown, K. Fujihashi, H. Kiyono, F. W. Smith, J. D. Beck, et al., “Inflammatory mediators of the terminal dentition in adult and early onset periodontitis,” *J Periodontal Res 33* (1998): 212–225, <https://doi.org/10.1111/j.1600-0765.1998.tb02193.x>.

86. G. A. Boyko, A. H. Melcher, and D. M. Brunette, “Formation of new periodontal ligament by periodontal ligament cells implanted in vivo after culture in vitro. A preliminary study of transplanted roots in the dog,” *J Periodontal Res 16* (1981): 73–88, <https://doi.org/10.1111/j.1600-0765.1981.tb00951.x>.

87. V. P. Terranova, L. C. Franzetti, S. Hic, R. M. DiFlorio, R. M. Lyall, U. M. Wikesjö, et al., “A biochemical approach to periodontal regeneration: tetracycline treatment of dentin promotes fibroblast adhesion and growth,” *J Periodontal Res 21* (1986): 330–337, <https://doi.org/10.1111/j.1600-0765.1986.tb01467.x>.

88. W. L. Gabler and H. R. Creamer, “Suppression of human neutrophil functions by tetracyclines,” *J Periodontal Res 26* (1991): 52–58, <https://doi.org/10.1111/j.1600-0765.1991.tb01626.x>.

89. M. A. Listgarten, “Nature of periodontal diseases: pathogenic mechanisms,” *J Periodontal Res 22* (1987): 172–178, <https://doi.org/10.1111/j.1600-0765.1987.tb01560.x>.

90. M. Crigger, G. Bogle, R. Nilvéus, J. Egelberg, and K. A. Selvig, “The effect of topical citric acid application on the healing of experimental furcation defects in dogs,” *J Periodontal Res 13* (1978): 538–549, <https://doi.org/10.1111/j.1600-0765.1978.tb00208.x>.

91. K. Yamazaki, T. Honda, T. Oda, K. Ueki-Maruyama, T. Nakajima, H. Yoshie, et al., “Effect of periodontal treatment on the C-reactive protein and proinflammatory cytokine levels in Japanese periodontitis patients,” *J Periodontal Res 40* (2005): 53–58, <https://doi.org/10.1111/j.1600-0765.2004.00772.x>.

92. C. A. Saxton and F. J. van der Ouderaa, “The effect of a dentifrice containing zinc citrate and Triclosan on developing gingivitis,” *J Periodontal Res 24* (1989): 75–80, <https://doi.org/10.1111/j.1600-0765.1989.tb00860.x>.

93. U. M. Wikesjö, P. J. Baker, L. A. Christersson, R. J. Genco, R. M. Lyall, S. Hic, et al., “A biochemical approach to periodontal regeneration: tetracycline treatment conditions dentin surfaces,” *J Periodontal Res 21* (1986): 322–329, <https://doi.org/10.1111/j.1600-0765.1986.tb01466.x>.

94. P. B. Robertson, M. Lantz, P. T. Marucha, K. S. Kornman, C. L. Trummel, and S. C. Holt, “Collagenolytic activity associated with Bacteroides species and Actinobacillus actinomycetemcomitans,” *J Periodontal Res 17* (1982): 275–283, <https://doi.org/10.1111/j.1600-0765.1982.tb01154.x>.

95. A. Contreras and J. Slots, “Herpesviruses in human periodontal disease,” *J Periodontal Res 35* (2000): 3–16, <https://doi.org/10.1034/j.1600-0765.2000.035001003.x>.

96. J. M. Goodson, M. A. Cugini, R. L. Kent, G. C. Armitage, C. M. Cobb, D. Fine, et al., “Multicenter evaluation of tetracycline fiber therapy: II. Clinical response,” *J Periodontal Res 26* (1991): 371–379, <https://doi.org/10.1111/j.1600-0765.1991.tb02076.x>.

97. D. Jönsson, D. Nebel, G. Bratthall, and B. O. Nilsson, “The human periodontal ligament cell: a fibroblast-like cell acting as an immune cell,” *J Periodontal Res 46* (2011): 153–157, <https://doi.org/10.1111/j.1600-0765.2010.01331.x>.

98. H. E. Schroeder and J. Theilade, “Electron microscopy of normal human gingival epithelium,” *J Periodontal Res 1* (1966): 95–119, <https://doi.org/10.1111/j.1600-0765.1966.tb01850.x>.

99. M. Wilson, K. Reddi, and B. Henderson, “Cytokine-inducing components of periodontopathogenic bacteria,” *J Periodontal Res 31* (1996): 393–407, <https://doi.org/10.1111/j.1600-0765.1996.tb00508.x>.

100. M. K. Jeffcoat, M. S. Reddy, R. L. Webber, R. C. Williams, and U. E. Ruttimann, “Extraoral control of geometry for digital subtraction radiography,” *J Periodontal Res 22* (1987): 396–402, <https://doi.org/10.1111/j.1600-0765.1987.tb01605.x>.

101. G. G. Nascimento, S. Alves-Costa, and M. Romandini, “Burden of severe periodontitis and edentulism in 2021, with projections up to 2050: The Global Burden of Disease 2021 study,” *J Periodontal Res 59* (2024): 823–867, <https://doi.org/10.1111/jre.13337>.

102. G. Isola, A. Polizzi, S. Santonocito, A. Alibrandi, P. Pesce, and T. Kocher, “Effect of quadrantwise versus full-mouth subgingival instrumentation on clinical and microbiological parameters in periodontitis patients: A randomized clinical trial,” *J Periodontal Res 59* (2024): 647–656, <https://doi.org/10.1111/jre.13279>.

103. G. Isola, S. Santonocito, A. Distefano, A. Polizzi, M. Vaccaro, G. Raciti, et al., “Impact of periodontitis on gingival crevicular fluid miRNAs profiles associated with cardiovascular disease risk,” *J Periodontal Res 58* (2023): 165–174, <https://doi.org/10.1111/jre.13078>.

104. L. B. Wong, A. U. Yap, and P. F. Allen, “Periodontal disease and quality of life: Umbrella review of systematic reviews,” *J Periodontal Res 56* (2021): 1–17, <https://doi.org/10.1111/jre.12805>.

105. G. Isola, A. Polizzi, A. Alibrandi, R. C. Williams, and A. Lo Giudice, “Analysis of galectin-3 levels as a source of coronary heart disease risk during periodontitis,” *J Periodontal Res 56* (2021): 597–605, <https://doi.org/10.1111/jre.12860>.

106. J. Long, Q. Cai, M. Steinwandel, M. K. Hargreaves, S. R. Bordenstein, W. J. Blot, et al., “Association of oral microbiome with type 2 diabetes risk,” *J Periodontal Res 52* (2017): 636–643, <https://doi.org/10.1111/jre.12432>.

107. G. Isola, A. Alibrandi, E. Rapisarda, G. Matarese, R. C. Williams, and R. Leonardi, “Association of vitamin D in patients with periodontitis: A cross-sectional study,” *J Periodontal Res 55* (2020): 602–612, <https://doi.org/10.1111/jre.12746>.

108. A. Antezack, D. Etchecopar-Etchart, B. La Scola, and V. Monnet-Corti, “New putative periodontopathogens and periodontal health-associated species: A systematic review and meta-analysis,” *J Periodontal Res 58* (2023): 893–906, <https://doi.org/10.1111/jre.13173>.

109. S. Zheng, S. Yu, X. Fan, Y. Zhang, Y. Sun, L. Lin, et al., “Porphyromonas gingivalis survival skills: Immune evasion,” *J Periodontal Res 56* (2021): 1007–1018, <https://doi.org/10.1111/jre.12915>.

110. E. M. Lu, “The role of vitamin D in periodontal health and disease,” *J Periodontal Res 58* (2023): 213–224, <https://doi.org/10.1111/jre.13083>.

111. I. Lafuente Ibáñez de Mendoza, X. Maritxalar Mendia, A. M. García de la Fuente, G. Quindós Andrés, and J. M. Aguirre Urizar, “Role of Porphyromonas gingivalis in oral squamous cell carcinoma development: A systematic review,” *J Periodontal Res 55* (2020): 13–22, <https://doi.org/10.1111/jre.12691>.

112. Y. Wu, B. He, Q. Chen, R. Yu, Y. Wu, H. Yang, et al., “Association between Mediterranean diet and periodontitis among US adults: The mediating roles of obesity indicators,” *J Periodontal Res 59* (2024): 32–41, <https://doi.org/10.1111/jre.13195>.

113. K. Apaza-Bedoya, M. Tarce, C. A. M. Benfatti, B. Henriques, M. T. Mathew, W. Teughels, et al., “Synergistic interactions between corrosion and wear at titanium-based dental implant connections: A scoping review,” *J Periodontal Res 52* (2017): 946–954, <https://doi.org/10.1111/jre.12469>.

114. H. Mohammad-Rahimi, S. R. Motamedian, Z. Pirayesh, A. Haiat, S. Zahedrozegar, E. Mahmoudinia, et al., “Deep learning in periodontology and oral implantology: A scoping review,” *J Periodontal Res 57* (2022): 942–951, <https://doi.org/10.1111/jre.13037>.

115. S. A. Danesh-Sani, S. P. Engebretson, and M. N. Janal, “Histomorphometric results of different grafting materials and effect of healing time on bone maturation after sinus floor augmentation: a systematic review and meta-analysis,” *J Periodontal Res 52* (2017): 301–312, <https://doi.org/10.1111/jre.12402>.

116. T. Tsuzuno, N. Takahashi, M. Yamada-Hara, M. Yokoji-Takeuchi, B. Sulijaya, Y. Aoki-Nonaka, et al., “Ingestion of Porphyromonas gingivalis exacerbates colitis via intestinal epithelial barrier disruption in mice,” *J Periodontal Res 56* (2021): 275–288, <https://doi.org/10.1111/jre.12816>.

117. W. Jiang, Y. Wang, Z. Cao, Y. Chen, C. Si, X. Sun, et al., “The role of mitochondrial dysfunction in periodontitis: From mechanisms to therapeutic strategy,” *J Periodontal Res 58* (2023): 853–863, <https://doi.org/10.1111/jre.13152>.

118. P. Han, P. M. Bartold, and S. Ivanovski, “The emerging role of small extracellular vesicles in saliva and gingival crevicular fluid as diagnostics for periodontitis,” *J Periodontal Res 57* (2022): 219–231, <https://doi.org/10.1111/jre.12950>.

119. E. D. de Avila, B. A. van Oirschot, and J. van den Beucken, “Biomaterial-based possibilities for managing peri-implantitis,” *J Periodontal Res 55* (2020): 165–173, <https://doi.org/10.1111/jre.12707>.

120. M. Galofré, D. Palao, M. Vicario, J. Nart, and D. Violant, “Clinical and microbiological evaluation of the effect of Lactobacillus reuteri in the treatment of mucositis and peri-implantitis: A triple-blind randomized clinical trial,” *J Periodontal Res 53* (2018): 378–390, <https://doi.org/10.1111/jre.12523>.

121. L. Yang, W. Tao, C. Xie, Q. Chen, Y. Zhao, L. Zhang, et al., “Interleukin-37 ameliorates periodontitis development by inhibiting NLRP3 inflammasome activation and modulating M1/M2 macrophage polarization,” *J Periodontal Res 59* (2024): 128–139, <https://doi.org/10.1111/jre.13196>.

122. J. Deng, C. Lu, Q. Zhao, K. Chen, S. Ma, and Z. Li, “The Th17/Treg cell balance: crosstalk among the immune system, bone and microbes in periodontitis,” *J Periodontal Res 57* (2022): 246–255, <https://doi.org/10.1111/jre.12958>.

123. K. Nonaka, Y. Kajiura, M. Bando, E. Sakamoto, Y. Inagaki, J. H. Lew, et al., “Advanced glycation end-products increase IL-6 and ICAM-1 expression via RAGE, MAPK and NF-κB pathways in human gingival fibroblasts,” *J Periodontal Res 53* (2018): 334–344, <https://doi.org/10.1111/jre.12518>.

124. S. A. Tassi, N. Z. Sergio, M. Y. O. Misawa, and C. C. Villar, “Efficacy of stem cells on periodontal regeneration: Systematic review of pre-clinical studies,” *J Periodontal Res 52* (2017): 793–812, <https://doi.org/10.1111/jre.12455>.

125. J. A. Keestra, I. Grosjean, W. Coucke, M. Quirynen, and W. Teughels, “Non-surgical periodontal therapy with systemic antibiotics in patients with untreated aggressive periodontitis: a systematic review and meta-analysis,” *J Periodontal Res 50* (2015): 689–706, <https://doi.org/10.1111/jre.12252>.

126. L. Pitzurra, I. D. C. Jansen, T. J. de Vries, M. A. Hoogenkamp, and B. G. Loos, “Effects of L-PRF and A-PRF+ on periodontal fibroblasts in in vitro wound healing experiments,” *J Periodontal Res 55* (2020): 287–295, <https://doi.org/10.1111/jre.12714>.

127. S. Ji, Y. S. Choi, and Y. Choi, “Bacterial invasion and persistence: critical events in the pathogenesis of periodontitis?,” *J Periodontal Res 50* (2015): 570–585, <https://doi.org/10.1111/jre.12248>.

128. G. Baima, M. Corana, G. Iaderosa, F. Romano, F. Citterio, G. Meoni, et al., “Metabolomics of gingival crevicular fluid to identify biomarkers for periodontitis: A systematic review with meta-analysis,” *J Periodontal Res 56* (2021): 633–645, <https://doi.org/10.1111/jre.12872>.

129. P. M. Duarte, C. R. Serrão, T. S. Miranda, L. C. Zanatta, M. F. Bastos, M. Faveri, et al., “Could cytokine levels in the peri-implant crevicular fluid be used to distinguish between healthy implants and implants with peri-implantitis? A systematic review,” *J Periodontal Res 51* (2016): 689–698, <https://doi.org/10.1111/jre.12354>.

130. M. Iwasaki, Y. Kimura, H. Ogawa, T. Yamaga, T. Ansai, T. Wada, et al., “Periodontitis, periodontal inflammation, and mild cognitive impairment: A 5-year cohort study,” *J Periodontal Res 54* (2019): 233–240, <https://doi.org/10.1111/jre.12623>.

131. J. G. S. Souza, B. E. Costa Oliveira, M. Bertolini, C. V. Lima, B. Retamal-Valdes, M. de Faveri, et al., “Titanium particles and ions favor dysbiosis in oral biofilms,” *J Periodontal Res 55* (2020): 258–266, <https://doi.org/10.1111/jre.12711>.

132. S. H. Jin, J. E. Lee, J. H. Yun, I. Kim, Y. Ko, and J. B. Park, “Isolation and characterization of human mesenchymal stem cells from gingival connective tissue,” *J Periodontal Res 50* (2015): 461–467, <https://doi.org/10.1111/jre.12228>.

133. D. Sokos, V. Everts, and T. J. de Vries, “Role of periodontal ligament fibroblasts in osteoclastogenesis: a review,” *J Periodontal Res 50* (2015): 152–159, <https://doi.org/10.1111/jre.12197>.

134. M. B. Asparuhova, D. Kiryak, M. Eliezer, D. Mihov, and A. Sculean, “Activity of two hyaluronan preparations on primary human oral fibroblasts,” *J Periodontal Res 54* (2019): 33–45, <https://doi.org/10.1111/jre.12602>.

135. R. Bright, K. Hynes, S. Gronthos, and P. M. Bartold, “Periodontal ligament-derived cells for periodontal regeneration in animal models: a systematic review,” *J Periodontal Res 50* (2015): 160–172, <https://doi.org/10.1111/jre.12205>.

136. Y. Li, J. Jiao, Y. Qi, W. Yu, S. Yang, J. Zhang, et al., “Curcumin: A review of experimental studies and mechanisms related to periodontitis treatment,” *J Periodontal Res 56* (2021): 837–847, <https://doi.org/10.1111/jre.12914>.

137. M. Landzberg, H. Doering, G. M. Aboodi, H. C. Tenenbaum, and M. Glogauer, “Quantifying oral inflammatory load: oral neutrophil counts in periodontal health and disease,” *J Periodontal Res 50* (2015): 330–336, <https://doi.org/10.1111/jre.12211>.

138. G. P. Harvey, T. R. Fitzsimmons, A. A. S. S. K. Dhamarpatni, C. Marchant, D. R. Haynes, and P. M. Bartold, “Expression of peptidylarginine deiminase-2 and -4, citrullinated proteins and anti-citrullinated protein antibodies in human gingiva,” *Journal of Periodontal Research 48* (2013): 252–261, <https://doi.org/https://doi.org/10.1111/jre.12002>.

139. Y. Shimizu, T. Takeda-Kawaguchi, I. Kuroda, Y. Hotta, H. Kawasaki, T. Hariyama, et al., “Exosomes from dental pulp cells attenuate bone loss in mouse experimental periodontitis,” *J Periodontal Res 57* (2022): 162–172, <https://doi.org/10.1111/jre.12949>.

140. K. Aral, M. R. Milward, Y. Kapila, A. Berdeli, and P. R. Cooper, “Inflammasomes and their regulation in periodontal disease: A review,” *J Periodontal Res 55* (2020): 473–487, <https://doi.org/10.1111/jre.12733>.

141. K. Algate, D. R. Haynes, P. M. Bartold, T. N. Crotti, and M. D. Cantley, “The effects of tumour necrosis factor-α on bone cells involved in periodontal alveolar bone loss; osteoclasts, osteoblasts and osteocytes,” *J Periodontal Res 51* (2016): 549–566, <https://doi.org/10.1111/jre.12339>.

142. F. O. Costa, E. J. Lages, L. O. Cota, T. C. Lorentz, R. V. Soares, and J. R. Cortelli, “Tooth loss in individuals under periodontal maintenance therapy: 5-year prospective study,” *J Periodontal Res 49* (2014): 121–128, <https://doi.org/10.1111/jre.12087>.

143. B. O. Nilsson, “Mechanisms involved in regulation of periodontal ligament cell production of pro-inflammatory cytokines: Implications in periodontitis,” *J Periodontal Res 56* (2021): 249–255, <https://doi.org/10.1111/jre.12823>.

144. M. G. Corrêa, P. R. Pires, F. V. Ribeiro, S. Z. Pimentel, R. C. Casarin, F. R. Cirano, et al., “Systemic treatment with resveratrol and/or curcumin reduces the progression of experimental periodontitis in rats,” *J Periodontal Res 52* (2017): 201–209, <https://doi.org/10.1111/jre.12382>.

145. P. M. Bartold, S. Gronthos, S. Ivanovski, A. Fisher, and D. W. Hutmacher, “Tissue engineered periodontal products,” *J Periodontal Res 51* (2016): 1–15, <https://doi.org/10.1111/jre.12275>.

146. K. M. Fawzy El-Sayed, M. Elahmady, Z. Adawi, N. Aboushadi, A. Elnaggar, M. Eid, et al., “The periodontal stem/progenitor cell inflammatory-regenerative cross talk: A new perspective,” *J Periodontal Res 54* (2019): 81–94, <https://doi.org/10.1111/jre.12616>.

147. C. A. Figueredo, R. Q. Catunda, M. P. Gibson, P. W. Major, and F. T. Almeida, “Use of ultrasound imaging for assessment of the periodontium: A systematic review,” *J Periodontal Res 59* (2024): 3–17, <https://doi.org/10.1111/jre.13194>.

148. Y. Ma, Y. Qian, Y. Chen, X. Ruan, X. Peng, Y. Sun, et al., “Resveratrol modulates the inflammatory response in hPDLSCs via the NRF2/HO-1 and NF-κB pathways and promotes osteogenic differentiation,” *J Periodontal Res 59* (2024): 162–173, <https://doi.org/10.1111/jre.13200>.

149. H. Chen, Y. Liu, S. Yu, C. Li, B. Gao, and X. Zhou, “Cannabidiol attenuates periodontal inflammation through inhibiting TLR4/NF-κB pathway,” *J Periodontal Res 58* (2023): 697–707, <https://doi.org/10.1111/jre.13118>.

150. Y. Liu, Q. Liu, Z. Li, A. Acharya, D. Chen, Z. Chen, et al., “Long non-coding RNA and mRNA expression profiles in peri-implantitis vs periodontitis,” *J Periodontal Res 55* (2020): 342–353, <https://doi.org/10.1111/jre.12718>.

151. X. Wang, Y. Li, Y. Feng, H. Cheng, and D. Li, “Macrophage polarization in aseptic bone resorption around dental implants induced by Ti particles in a murine model,” *J Periodontal Res 54* (2019): 329–338, <https://doi.org/10.1111/jre.12633>.

152. T. Kagiya and S. Nakamura, “Expression profiling of microRNAs in RAW264.7 cells treated with a combination of tumor necrosis factor alpha and RANKL during osteoclast differentiation,” *J Periodontal Res 48* (2013): 373–385, <https://doi.org/10.1111/jre.12017>.

153. I. Saygun, N. Nizam, I. Keskiner, V. Bal, A. Kubar, C. Açıkel, et al., “Salivary infectious agents and periodontal disease status,” *J Periodontal Res 46* (2011): 235–239, <https://doi.org/10.1111/j.1600-0765.2010.01335.x>.

154. S. Liu, M. Zhou, J. Li, B. Hu, D. Jiang, H. Huang, et al., “LIPUS inhibited the expression of inflammatory factors and promoted the osteogenic differentiation capacity of hPDLCs by inhibiting the NF-κB signaling pathway,” *J Periodontal Res 55* (2020): 125–140, <https://doi.org/10.1111/jre.12696>.

155. T. Maekawa and G. Hajishengallis, “Topical treatment with probiotic Lactobacillus brevis CD2 inhibits experimental periodontal inflammation and bone loss,” *J Periodontal Res 49* (2014): 785–791, <https://doi.org/10.1111/jre.12164>.

156. W. Guo, Y. Zhao, H. Li, and L. Lei, “NCOA4-mediated ferritinophagy promoted inflammatory responses in periodontitis,” *J Periodontal Res 56* (2021): 523–534, <https://doi.org/10.1111/jre.12852>.

157. J. J. Mikkonen, S. P. Singh, M. Herrala, R. Lappalainen, S. Myllymaa, and A. M. Kullaa, “Salivary metabolomics in the diagnosis of oral cancer and periodontal diseases,” *J Periodontal Res 51* (2016): 431–437, <https://doi.org/10.1111/jre.12327>.

158. A. S. Ertugrul, H. Sahin, A. Dikilitas, N. Alpaslan, and A. Bozoglan, “Comparison of CCL28, interleukin-8, interleukin-1β and tumor necrosis factor-alpha in subjects with gingivitis, chronic periodontitis and generalized aggressive periodontitis,” *J Periodontal Res 48* (2013): 44–51, <https://doi.org/10.1111/j.1600-0765.2012.01500.x>.

159. E. F. de Morais, J. C. Pinheiro, R. B. Leite, P. P. A. Santos, C. A. G. Barboza, and R. A. Freitas, “Matrix metalloproteinase-8 levels in periodontal disease patients: A systematic review,” *J Periodontal Res 53* (2018): 156–163, <https://doi.org/10.1111/jre.12495>.

160. R. P. Teles, V. Likhari, S. S. Socransky, and A. D. Haffajee, “Salivary cytokine levels in subjects with chronic periodontitis and in periodontally healthy individuals: a cross-sectional study,” *J Periodontal Res 44* (2009): 411–417, <https://doi.org/10.1111/j.1600-0765.2008.01119.x>.

161. E. Albuquerque-Souza, D. Balzarini, E. S. Ando-Suguimoto, K. H. Ishikawa, M. R. L. Simionato, M. Holzhausen, et al., “Probiotics alter the immune response of gingival epithelial cells challenged by Porphyromonas gingivalis,” *J Periodontal Res 54* (2019): 115–127, <https://doi.org/10.1111/jre.12608>.

162. K. Deng, S. Wei, M. Xu, J. Shi, H. Lai, and M. S. Tonetti, “Diagnostic accuracy of active matrix metalloproteinase-8 point-of-care test for the discrimination of periodontal health status: Comparison of saliva and oral rinse samples,” *J Periodontal Res 57* (2022): 768–779, <https://doi.org/10.1111/jre.12999>.

163. X. Gao, S. Li, W. Wang, X. Zhang, X. Yu, C. Fan, et al., “Caspase-3 and gasdermin E mediate macrophage pyroptosis in periodontitis,” *J Periodontal Res 59* (2024): 140–150, <https://doi.org/10.1111/jre.13197>.

164. N. D. Pakpahan, M. Kyawsoewin, J. Manokawinchoke, C. Termkwancharoen, H. Egusa, P. Limraksasin, et al., “Effects of mechanical loading on matrix homeostasis and differentiation potential of periodontal ligament cells: A scoping review,” *J Periodontal Res 59* (2024): 877–906, <https://doi.org/10.1111/jre.13284>.

165. V. Lekovic, I. Milinkovic, Z. Aleksic, S. Jankovic, P. Stankovic, E. B. Kenney, et al., “Platelet-rich fibrin and bovine porous bone mineral vs. platelet-rich fibrin in the treatment of intrabony periodontal defects,” *J Periodontal Res 47* (2012): 409–417, <https://doi.org/10.1111/j.1600-0765.2011.01446.x>.

166. W. Kang, Q. Liang, L. Du, L. Shang, T. Wang, and S. Ge, “Sequential application of bFGF and BMP-2 facilitates osteogenic differentiation of human periodontal ligament stem cells,” *J Periodontal Res 54* (2019): 424–434, <https://doi.org/10.1111/jre.12644>.

167. E. Calciolari, P. Ercal, M. Dourou, A. Akcali, S. Tagliaferri, and N. Donos, “The efficacy of adjunctive periodontal therapies during supportive periodontal care in patients with residual pockets. A systematic review and meta-analysis,” *J Periodontal Res 57* (2022): 671–689, <https://doi.org/10.1111/jre.13001>.

168. O. A. González, C. Escamilla, R. J. Danaher, J. Dai, J. L. Ebersole, R. J. Mumper, et al., “Antibacterial effects of blackberry extract target periodontopathogens,” *Journal of Periodontal Research 48* (2013): 80–86, <https://doi.org/https://doi.org/10.1111/j.1600-0765.2012.01506.x>.

169. S. Reichert, A. Schlitt, V. Beschow, A. Lutze, S. Lischewski, T. Seifert, et al., “Use of floss/interdental brushes is associated with lower risk for new cardiovascular events among patients with coronary heart disease,” *Journal of Periodontal Research 50* (2015): 180–188, <https://doi.org/https://doi.org/10.1111/jre.12191>.

170. I. Laleman, R. Koop, W. Teughels, C. Dekeyser, and M. Quirynen, “Influence of tongue brushing and scraping on the oral microflora of periodontitis patients,” *J Periodontal Res 53* (2018): 73–79, <https://doi.org/10.1111/jre.12489>.

171. T. Maruyama, T. Kobayashi, Y. Sugiura, T. Yoneda, D. Ekuni, and M. Morita, “Association between serum miRNAs and gingival gene expression in an obese rat model,” *J Periodontal Res 57* (2022): 502–509, <https://doi.org/10.1111/jre.12979>.

172. I. He, B. Poirier, E. Jensen, S. Kaur, J. Hedges, S. Jesudason, et al., “Demystifying the connection between periodontal disease and chronic kidney disease - An umbrella review,” *J Periodontal Res 58* (2023): 874–892, <https://doi.org/10.1111/jre.13161>.

173. C. R. Schiött and H. Löe, “The origin and variation in number of leukocytes in the human saliva,” *J Periodontal Res 5* (1970): 36–41, <https://doi.org/10.1111/j.1600-0765.1970.tb01835.x>.

174. P. Jirasek, A. Jusku, J. Frankova, M. Urbankova, D. Diabelko, F. Ruzicka, et al., “Phytocannabinoids and gingival inflammation: Preclinical findings and a placebo-controlled double-blind randomized clinical trial with cannabidiol,” *J Periodontal Res 59* (2024): 468–479, <https://doi.org/10.1111/jre.13234>.

175. N. Castro Dos Santos, M. R. Westphal, B. Retamal-Valdes, P. M. Duarte, L. C. Figueiredo, M. Faveri, et al., “Influence of gender on periodontal outcomes: A retrospective analysis of eight randomized clinical trials,” *J Periodontal Res 59* (2024): 1175–1183, <https://doi.org/10.1111/jre.13272>.

176. D. Bourgeois, P. Bouchard, and C. Mattout, “Epidemiology of periodontal status in dentate adults in France, 2002-2003,” *J Periodontal Res 42* (2007): 219–227, <https://doi.org/10.1111/j.1600-0765.2006.00936.x>.

177. X. Wu, S. Offenbacher, N. J. Lόpez, D. Chen, H. Y. Wang, J. Rogus, et al., “Association of interleukin-1 gene variations with moderate to severe chronic periodontitis in multiple ethnicities,” *J Periodontal Res 50* (2015): 52–61, <https://doi.org/10.1111/jre.12181>.

178. L. de Camargo, S. N. da Silva, and L. Chambrone, “Efficacy of toothbrushing procedures performed in intensive care units in reducing the risk of ventilator-associated pneumonia: A systematic review,” *J Periodontal Res 54* (2019): 601–611, <https://doi.org/10.1111/jre.12668>.

179. J. Lönn, S. Ljunggren, K. Klarström-Engström, I. Demirel, T. Bengtsson, and H. Karlsson, “Lipoprotein modifications by gingipains of Porphyromonas gingivalis,” *J Periodontal Res 53* (2018): 403–413, <https://doi.org/10.1111/jre.12527>.

180. C. Ren, C. McGrath, L. Jin, C. Zhang, and Y. Yang, “The effectiveness of low-level laser therapy as an adjunct to non-surgical periodontal treatment: a meta-analysis,” *J Periodontal Res 52* (2017): 8–20, <https://doi.org/10.1111/jre.12361>.

181. F. Sgolastra, A. Petrucci, I. Ciarrocchi, C. Masci, and A. Spadaro, “Adjunctive systemic antimicrobials in the treatment of chronic periodontitis: A systematic review and network meta-analysis,” *J Periodontal Res 56* (2021): 236–248, <https://doi.org/10.1111/jre.12821>.

182. V. Ilievski, U. G. Bhat, S. Suleiman-Ata, B. A. Bauer, P. T. Toth, S. T. Olson, et al., “Oral application of a periodontal pathogen impacts SerpinE1 expression and pancreatic islet architecture in prediabetes,” *J Periodontal Res 52* (2017): 1032–1041, <https://doi.org/10.1111/jre.12474>.

183. A. Khocht, M. Orlich, B. Paster, D. Bellinger, L. Lenoir, C. Irani, et al., “Cross-sectional comparisons of subgingival microbiome and gingival fluid inflammatory cytokines in periodontally healthy vegetarians versus non-vegetarians,” *J Periodontal Res 56* (2021): 1079–1090, <https://doi.org/10.1111/jre.12922>.

184. L. Nibali, J. Bayliss-Chapman, H. Halai, C. Somani, J. Davies, P. Ancliff, et al., “Periodontal status in children with primary immunodeficiencies,” *J Periodontal Res 56* (2021): 819–827, <https://doi.org/10.1111/jre.12880>.

185. A. J. McKendrick, L. M. Barbenel, and W. D. McHugh, “A two-year comparison of hand and electric toothbrushes,” *J Periodontal Res 3* (1968): 224–231, <https://doi.org/10.1111/j.1600-0765.1968.tb01924.x>.

186. J. Zhu, X. Li, F. Zhu, L. Chen, C. Zhang, C. McGrath, et al., “Multiple tooth loss is associated with vascular cognitive impairment in subjects with acute ischemic stroke,” *J Periodontal Res 50* (2015): 683–688, <https://doi.org/10.1111/jre.12251>.

187. D. M. Foulkes, “Some toxicological observations on chlorhexidine,” *J Periodontal Res Suppl 12* (1973): 55–60, <https://doi.org/10.1111/j.1600-0765.1973.tb02165.x>.

188. Y. M. Henskens, P. A. van den Keijbus, E. C. Veerman, G. A. Van der Weijden, M. F. Timmerman, C. M. Snoek, et al., “Protein composition of whole and parotid saliva in healthy and periodontitis subjects. Determination of cystatins, albumin, amylase and IgA,” *J Periodontal Res 31* (1996): 57–65, <https://doi.org/10.1111/j.1600-0765.1996.tb00464.x>.

189. S. Y. Rawal, M. K. Dabbous, and D. A. Tipton, “Effect of cannabidiol on human gingival fibroblast extracellular matrix metabolism: MMP production and activity, and production of fibronectin and transforming growth factor β,” *Journal of Periodontal Research 47* (2012): 320–329, <https://doi.org/https://doi.org/10.1111/j.1600-0765.2011.01435.x>.

190. S. Yamada, T. Komiyama, T. Ohi, T. Murakami, Y. Miyoshi, K. Endo, et al., “Regular dental visits, periodontitis, tooth loss, and atherosclerosis: The Ohasama study,” *Journal of Periodontal Research 57* (2022): 615–622, <https://doi.org/https://doi.org/10.1111/jre.12990>.

191. S. Novello, A. Debouche, M. Philippe, F. Naudet, and S. Jeanne, “Clinical application of mesenchymal stem cells in periodontal regeneration: A systematic review and meta-analysis,” *J Periodontal Res 55* (2020): 1–12, <https://doi.org/10.1111/jre.12684>.

192. Z. Zhang, Y. Zheng, and X. Bian, “Clinical effect of azithromycin as an adjunct to non-surgical treatment of chronic periodontitis: a meta-analysis of randomized controlled clinical trials,” *J Periodontal Res 51* (2016): 275–283, <https://doi.org/10.1111/jre.12319>.

193. L. P. Menzel, W. Ruddick, M. H. Chowdhury, D. C. Brice, R. Clance, E. Porcelli, et al., “Activation of vitamin D in the gingival epithelium and its role in gingival inflammation and alveolar bone loss,” *J Periodontal Res 54* (2019): 444–452, <https://doi.org/10.1111/jre.12646>.

194. H. Balci Yuce, A. Lektemur Alpan, F. Gevrek, and H. Toker, “Investigation of the effect of astaxanthin on alveolar bone loss in experimental periodontitis,” *J Periodontal Res 53* (2018): 131–138, <https://doi.org/10.1111/jre.12497>.

195. L. P. Mau, Y. C. Kuan, Y. C. Tsai, J. J. Lin, G. Huynh-Ba, P. W. Weng, et al., “Patients with chronic periodontitis present increased risk for osteoporosis: A population-based cohort study in Taiwan,” *J Periodontal Res 52* (2017): 922–929, <https://doi.org/10.1111/jre.12464>.

196. N. Kobayashi, K. Ishihara, N. Sugihara, M. Kusumoto, M. Yakushiji, and K. Okuda, “Colonization pattern of periodontal bacteria in Japanese children and their mothers,” *J Periodontal Res 43* (2008): 156–161, <https://doi.org/10.1111/j.1600-0765.2007.01005.x>.

197. A. Chaparro, A. Sanz, A. Quintero, C. Inostroza, V. Ramirez, F. Carrion, et al., “Increased inflammatory biomarkers in early pregnancy is associated with the development of pre-eclampsia in patients with periodontitis: a case control study,” *J Periodontal Res 48* (2013): 302–307, <https://doi.org/10.1111/jre.12008>.

198. N. C. Castro Dos Santos, M. V. Furukawa, I. Oliveira-Cardoso, J. R. Cortelli, M. Feres, T. Van Dyke, et al., “Does the use of omega-3 fatty acids as an adjunct to non-surgical periodontal therapy provide additional benefits in the treatment of periodontitis? A systematic review and meta-analysis,” *J Periodontal Res 57* (2022): 435–447, <https://doi.org/10.1111/jre.12984>.

199. L. A. Chisini, M. G. Cademartori, A. Francia, M. Mederos, G. Grazioli, M. C. M. Conde, et al., “Is the use of Cannabis associated with periodontitis? A systematic review and meta-analysis,” *J Periodontal Res 54* (2019): 311–317, <https://doi.org/10.1111/jre.12639>.

200. P. B. Imrey, N. W. Chilton, B. L. Pihlstrom, H. M. Proskin, A. Kingman, M. A. Listgarten, et al., “Recommended revisions to American Dental Association guidelines for acceptance of chemotherapeutic products for gingivitis control. Report of the Task Force on Design and Analysis in Dental and Oral Research to the Council on Therapeutics of the American Dental Association,” *J Periodontal Res 29* (1994): 299–304, <https://doi.org/10.1111/j.1600-0765.1994.tb01225.x>.

201. T. Yamamoto, T. Domon, S. Takahashi, N. Islam, R. Suzuki, and M. Wakita, “The structure and function of the cemento-dentinal junction in human teeth,” *J Periodontal Res 34* (1999): 261–268, <https://doi.org/10.1111/j.1600-0765.1999.tb02252.x>.

202. Z. Yu, N. S. Ramamurthy, M. Leung, K. M. Chang, T. F. McNamara, and L. M. Golub, “Chemically-modified tetracycline normalizes collagen metabolism in diabetic rats: a dose-response study,” *J Periodontal Res 28* (1993): 420–428, <https://doi.org/>

203. Z. Akram, F. Vohra, and F. Javed, “Locally delivered metformin as adjunct to scaling and root planing in the treatment of periodontal defects: A systematic review and meta-analysis,” *J Periodontal Res 53* (2018): 941–949, <https://doi.org/10.1111/jre.12573>.

204. M. P. Bertrand-Duchesne, D. Grenier, and G. Gagnon, “Epidermal growth factor released from platelet-rich plasma promotes endothelial cell proliferation in vitro,” *J Periodontal Res 45* (2010): 87–93, <https://doi.org/10.1111/j.1600-0765.2009.01205.x>.

205. J. S. Bender, H. Thang, and M. Glogauer, “Novel rinse assay for the quantification of oral neutrophils and the monitoring of chronic periodontal disease,” *J Periodontal Res 41* (2006): 214–220, <https://doi.org/10.1111/j.1600-0765.2005.00861.x>.

206. R. N. Smith, R. N. Andersen, and P. E. Kolenbrander, “Inhibition of intergeneric coaggregation among oral bacteria by cetylpyridinium chloride, chlorhexidine digluconate and octenidine dihydrochloride,” *J Periodontal Res 26* (1991): 422–428, <https://doi.org/10.1111/j.1600-0765.1991.tb01732.x>.

207. N. Tani-Ishii, A. Tsunoda, and T. Umemoto, “Osteopontin antisense deoxyoligonucleotides inhibit bone resorption by mouse osteoclasts in vitro,” *J Periodontal Res 32* (1997): 480–486, <https://doi.org/10.1111/j.1600-0765.1997.tb00563.x>.

208. J. Tang-Larsen, R. Claesson, M. B. Edlund, and J. Carlsson, “Competition for peptides and amino acids among periodontal bacteria,” *J Periodontal Res 30* (1995): 390–395, <https://doi.org/10.1111/j.1600-0765.1995.tb01292.x>.

209. F. W. Pirk, T. M. ElAttar, and G. D. Roth, “Effect of analogues of steroid and thyroxine hormones on wound healing in hamsters,” *J Periodontal Res 9* (1974): 290–297, <https://doi.org/10.1111/j.1600-0765.1974.tb00684.x>.

210. M. Friedman and G. Golomb, “New sustained release dosage form of chlorhexidine for dental use. I. Development and kinetics of release,” *J Periodontal Res 17* (1982): 323–328, <https://doi.org/10.1111/j.1600-0765.1982.tb01160.x>.

211. N. R. Rojo-Botello, A. L. García-Hernández, and L. Moreno-Fierros, “Expression of toll-like receptors 2, 4 and 9 is increased in gingival tissue from patients with type 2 diabetes and chronic periodontitis,” *J Periodontal Res 47* (2012): 62–73, <https://doi.org/10.1111/j.1600-0765.2011.01405.x>.

212. K. Z. Liu, X. M. Xiang, A. Man, M. G. Sowa, A. Cholakis, E. Ghiabi, et al., “In vivo determination of multiple indices of periodontal inflammation by optical spectroscopy,” *J Periodontal Res 44* (2009): 117–124, <https://doi.org/10.1111/j.1600-0765.2008.01112.x>.

213. H. Suido, M. E. Neiders, P. K. Barua, M. Nakamura, P. A. Mashimo, and R. J. Genco, “Characterization of N-CBz-glycyl-glycyl-arginyl peptidase and glycyl-prolyl peptidase of Bacteroides gingivalis,” *J Periodontal Res 22* (1987): 412–418, <https://doi.org/10.1111/j.1600-0765.1987.tb01608.x>.

214. A. H. Huynh, P. D. Veith, N. R. McGregor, G. G. Adams, D. Chen, E. C. Reynolds, et al., “Gingival crevicular fluid proteomes in health, gingivitis and chronic periodontitis,” *J Periodontal Res 50* (2015): 637–649, <https://doi.org/10.1111/jre.12244>.

215. J. D. Beck, T. Sharp, G. G. Koch, and S. Offenbacher, “A 5-year study of attachment loss and tooth loss in community-dwelling older adults,” *J Periodontal Res 32* (1997): 516–523, <https://doi.org/10.1111/j.1600-0765.1997.tb00567.x>.

216. D. Hagenfeld, K. Prior, I. Harks, Y. Jockel-Schneider, T. W. May, D. Harmsen, et al., “No differences in microbiome changes between anti-adhesive and antibacterial ingredients in toothpastes during periodontal therapy,” *J Periodontal Res 54* (2019): 435–443, <https://doi.org/10.1111/jre.12645>.

217. S. Mizutani, D. Ekuni, T. Tomofuji, T. Azuma, K. Kataoka, M. Yamane, et al., “Relationship between xerostomia and gingival condition in young adults,” *J Periodontal Res 50* (2015): 74–79, <https://doi.org/10.1111/jre.12183>.

218. Y. M. M. Fernandez, R. A. M. Exterkate, M. J. Buijs, W. Beertsen, G. A. van der Weijden, E. Zaura, et al., “A reproducible microcosm biofilm model of subgingival microbial communities,” *J Periodontal Res 52* (2017): 1021–1031, <https://doi.org/10.1111/jre.12473>.

219. S. W. H. Rademacher, E. Zaura, C. J. Kleverlaan, M. J. Buijs, W. Crielaard, B. G. Loos, et al., “Qualitative and quantitative differences in the subgingival microbiome of the restored and unrestored teeth,” *J Periodontal Res 54* (2019): 405–412, <https://doi.org/10.1111/jre.12642>.

220. B. Alkaya, I. Laleman, S. Keceli, O. Ozcelik, M. Cenk Haytac, and W. Teughels, “Clinical effects of probiotics containing Bacillus species on gingivitis: a pilot randomized controlled trial,” *J Periodontal Res 52* (2017): 497–504, <https://doi.org/10.1111/jre.12415>.

221. C. C. Tsai, H. H. Chou, T. L. Wu, Y. H. Yang, K. Y. Ho, Y. M. Wu, et al., “The levels of volatile sulfur compounds in mouth air from patients with chronic periodontitis,” *J Periodontal Res 43* (2008): 186–193, <https://doi.org/10.1111/j.1600-0765.2007.01011.x>.

222. J. S. Park, E. Kim, S. J. Kwon, J. S. Heo, and K. H. Ahn, “Differences in maternal subgingival microbiome between preterm and term births: The MOHEPI study,” *J Periodontal Res 59* (2024): 939–950, <https://doi.org/10.1111/jre.13292>.

223. T. Takemoto, M. Ozaki, M. Shirakawa, T. Hino, and H. Okamoto, “Purification of arginine-sensitive hemagglutinin from Fusobacterium nucleatum and its role in coaggregation,” *J Periodontal Res 28* (1993): 21–26, <https://doi.org/10.1111/j.1600-0765.1993.tb01046.x>.

224. A. Brun, A. Nuzzo, B. Prouvost, D. Diallo, S. Hamdan, E. Meseguer, et al., “Oral microbiota and atherothrombotic carotid plaque vulnerability in periodontitis patients. A cross-sectional study,” *J Periodontal Res 56* (2021): 339–350, <https://doi.org/10.1111/jre.12826>.

225. M. Khosravisamani, G. Maliji, S. Seyfi, A. Azadmehr, B. Abd Nikfarjam, S. Madadi, et al., “Effect of the menstrual cycle on inflammatory cytokines in the periodontium,” *J Periodontal Res 49* (2014): 770–776, <https://doi.org/10.1111/jre.12161>.

226. L. Heijl, J. Wennström, J. Lindhe, and S. S. Socransky, “Periodontal disease in gnotobiotic rats,” *J Periodontal Res 15* (1980): 405–419, <https://doi.org/10.1111/j.1600-0765.1980.tb00298.x>.

227. S. Li, X. Liu, H. Li, H. Pan, A. Acharya, Y. Deng, et al., “Integrated analysis of long noncoding RNA-associated competing endogenous RNA network in periodontitis,” *J Periodontal Res 53* (2018): 495–505, <https://doi.org/10.1111/jre.12539>.

228. D. Yang, R. Liu, L. Liu, H. Liao, C. Wang, and Z. Cao, “Involvement of CD147 in alveolar bone remodeling and soft tissue degradation in experimental periodontitis,” *J Periodontal Res 52* (2017): 704–712, <https://doi.org/10.1111/jre.12435>.

229. R. C. G. de Oliveira, E. Gardev, and L. M. Shaddox, “Dysbiotic relationship between arthritis and the oral-gut microbiome. A critical review,” *J Periodontal Res 57* (2022): 711–723, <https://doi.org/10.1111/jre.13002>.

230. L. M. B. Ambrósio, E. S. Rovai, D. I. Sendyk, M. Holzhausen, and C. M. Pannuti, “Does the adjunctive use of statins provide additional benefits to nonsurgical periodontal treatment? A systematic review and meta-analysis,” *J Periodontal Res 53* (2018): 12–21, <https://doi.org/10.1111/jre.12480>.

231. G. J. Seymour, J. E. Palmer, S. J. Leishman, H. L. Do, B. Westerman, A. D. Carle, et al., “Influence of a triclosan toothpaste on periodontopathic bacteria and periodontitis progression in cardiovascular patients: a randomized controlled trial,” *J Periodontal Res 52* (2017): 61–73, <https://doi.org/10.1111/jre.12369>.

232. C. M. Faggion, Jr., M. P. Cullinan, and M. Atieh, “An overview of systematic reviews on the effectiveness of periodontal treatment to improve glycaemic control,” *J Periodontal Res 51* (2016): 716–725, <https://doi.org/10.1111/jre.12358>.

233. T. Hamasaki, M. Kitamura, Y. Kawashita, Y. Ando, and T. Saito, “Periodontal disease and percentage of calories from fat using national data,” *J Periodontal Res 52* (2017): 114–121, <https://doi.org/10.1111/jre.12375>.

234. K. Sato, M. Yokoji, M. Yamada, T. Nakajima, and K. Yamazaki, “An orally administered oral pathobiont and commensal have comparable and innocuous systemic effects in germ-free mice,” *J Periodontal Res 53* (2018): 950–960, <https://doi.org/10.1111/jre.12593>.

235. Y. Moritani, M. Usui, K. Sano, K. Nakazawa, T. Hanatani, M. Nakatomi, et al., “Spheroid culture enhances osteogenic potential of periodontal ligament mesenchymal stem cells,” *J Periodontal Res 53* (2018): 870–882, <https://doi.org/10.1111/jre.12577>.

236. H. Fang, M. Han, Q. L. Li, C. Y. Cao, R. Xia, and Z. H. Zhang, “Comparison of full-mouth disinfection and quadrant-wise scaling in the treatment of adult chronic periodontitis: a systematic review and meta-analysis,” *J Periodontal Res 51* (2016): 417–430, <https://doi.org/10.1111/jre.12326>.

237. T. B. Lombardo Bedran, K. Feghali, L. Zhao, D. M. Palomari Spolidorio, and D. Grenier, “Green tea extract and its major constituent, epigallocatechin-3-gallate, induce epithelial beta-defensin secretion and prevent beta-defensin degradation by Porphyromonas gingivalis,” *J Periodontal Res 49* (2014): 615–623, <https://doi.org/10.1111/jre.12142>.

238. C. Oh, H. J. Kim, and H. M. Kim, “Transepithelial channels for leukocytes in the junctional epithelium,” *J Periodontal Res 57* (2022): 1093–1100, <https://doi.org/10.1111/jre.13043>.

239. J. Caton, “Biological and measurement issues critical to design of gingivitis trials,” *J Periodontal Res 27* (1992): 364–368; discussion 373–364, <https://doi.org/10.1111/j.1600-0765.1992.tb01697.x>.

240. J. Y. Hong, W. J. Bae, J. K. Yi, G. T. Kim, and E. C. Kim, “Anti-inflammatory and anti-osteoclastogenic effects of zinc finger protein A20 overexpression in human periodontal ligament cells,” *J Periodontal Res 51* (2016): 529–539, <https://doi.org/10.1111/jre.12332>.

241. H. Shen, Y. Jiang, C. Qiu, X. Xie, H. Zhang, Z. He, et al., “Abnormal amyloid precursor protein processing in periodontal tissue in a murine model of periodontitis induced by Porphyromonas gingivalis,” *J Periodontal Res 59* (2024): 395–407, <https://doi.org/10.1111/jre.13224>.
