# Supplementary figures and images for: Mapping 60 Years of Discovery: An AI‐Driven Bibliometric and Altmetric Analysis of the Journal of Periodontal Research
Source: J Periodontal Res. 2025 Dec 30;60(12):1181–200. doi: 10.1111/jre.70071 (PMC12881886; doi:10.1111/jre.70071)

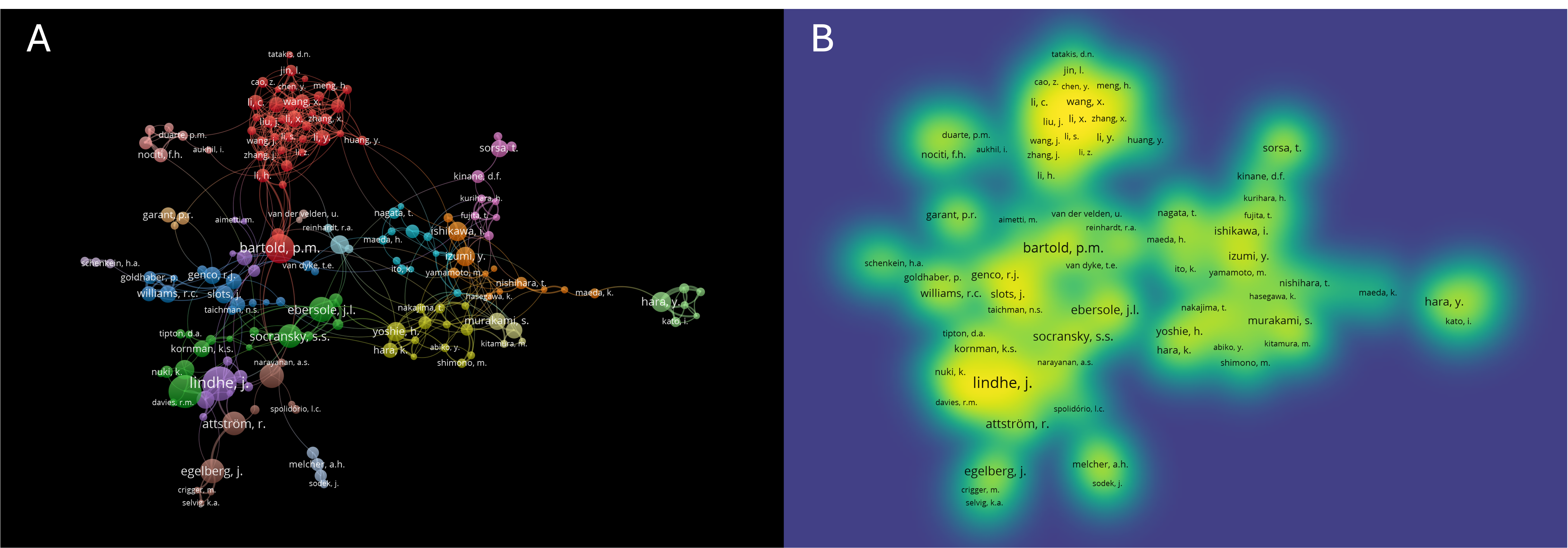

Supplement: Supplementary file 1 — Data S1: jre70071‐sup‐0002‐DataS1.zip. [file JRE-60-1181-s002.zip › jre70071-sup-0002-FigureS1.png]

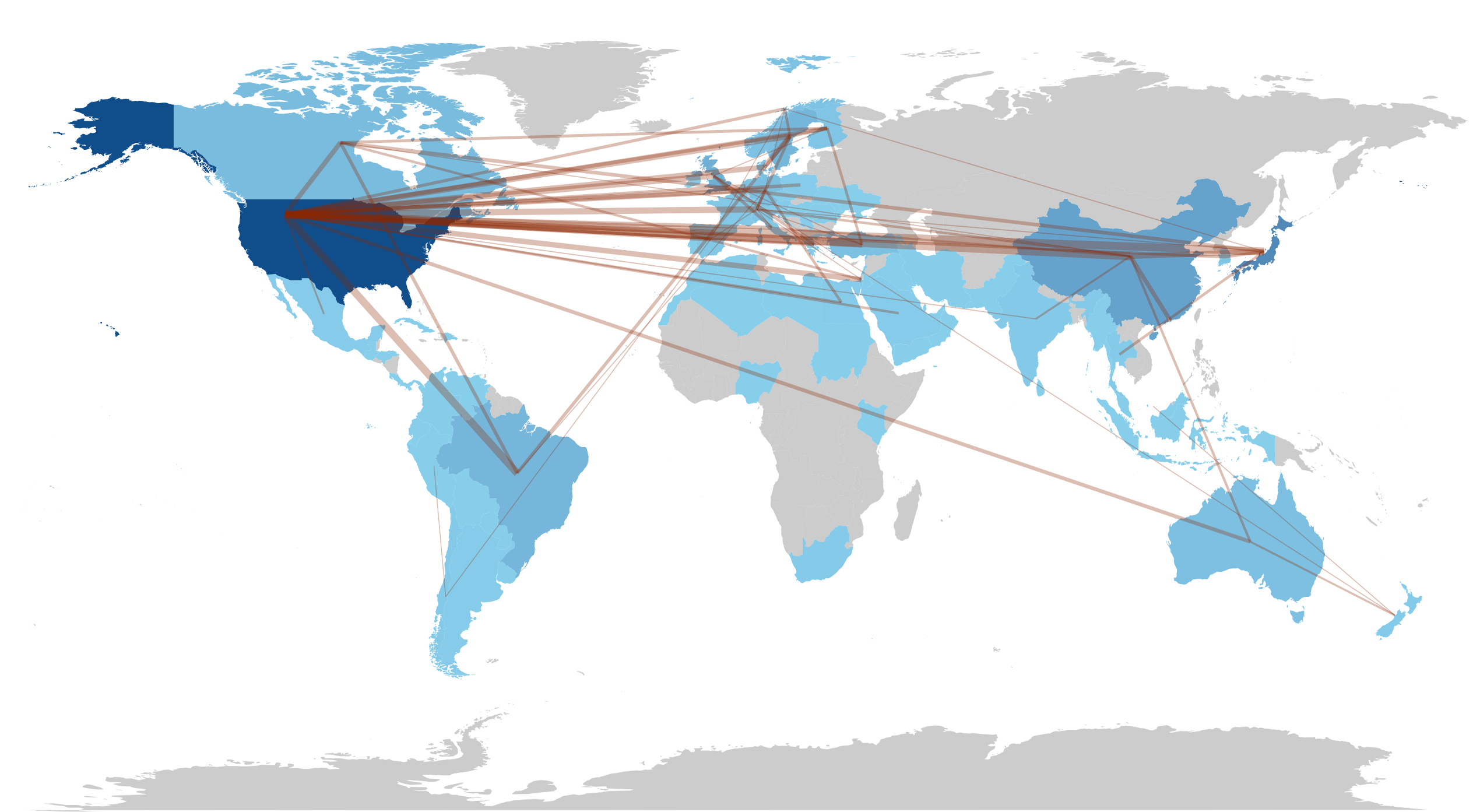

Supplement: Supplementary file 1 — Data S1: jre70071‐sup‐0002‐DataS1.zip. [file JRE-60-1181-s002.zip › jre70071-sup-0003-FigureS2.png]

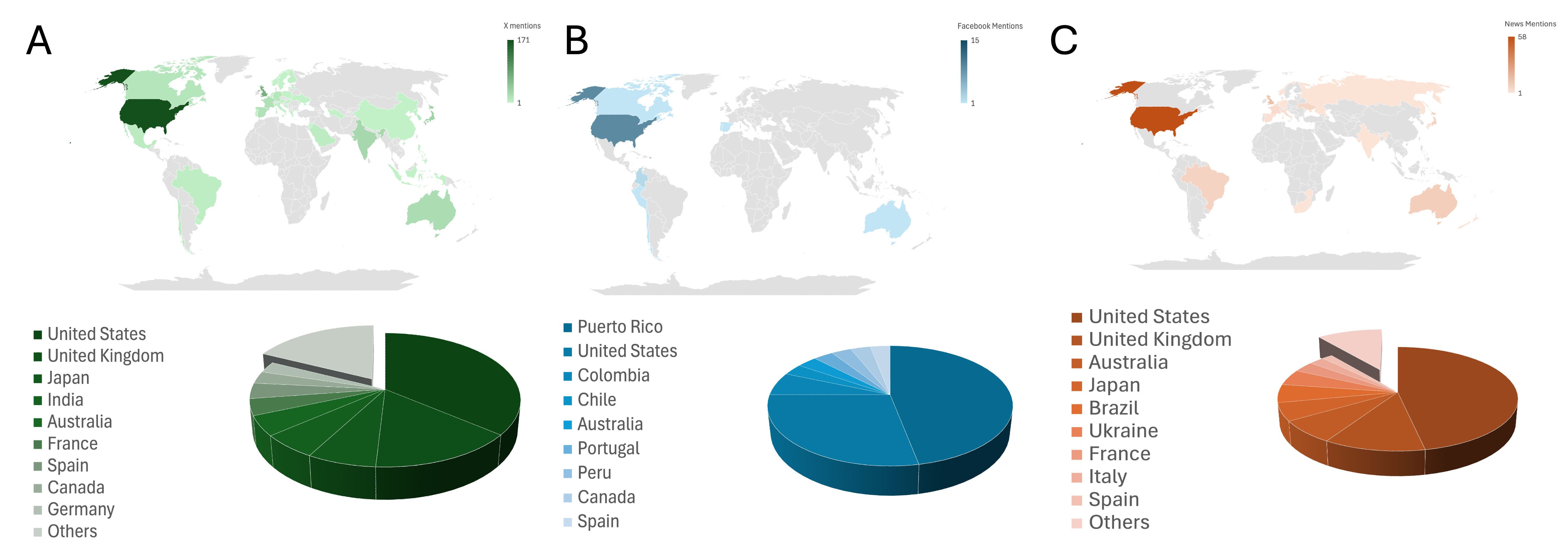

Supplement: Supplementary file 1 — Data S1: jre70071‐sup‐0002‐DataS1.zip. [file JRE-60-1181-s002.zip › jre70071-sup-0004-FigureS3.png]
