# Supplementary material for: Mapping 60 Years of Discovery: An AI‐Driven Bibliometric and Altmetric Analysis of the Journal of Periodontal Research
Source: J Periodontal Res. 2025 Dec 30;60(12):1181–200. doi: 10.1111/jre.70071 (PMC12881886; doi:10.1111/jre.70071)
Supplement: Supplementary file 2 — Data S2: jre7001‐sup‐0002‐DataS2.docx. [file JRE-60-1181-s001.docx]

**Appendix S1** the Codebooks of Topics and Levels of Evidence

**A.1. Topics Codebook**

**1) Epidemiology, Risk, and Public Health**

**Definition:** population patterns, prevalence/incidence, risk/protective factors, staging/grading tools, screening, access/disparities, care delivery models, economics, and PROMs.

**Include:** prevalence/incidence; cohort/case–control; risk calculators/scores; grading validation; screening/triage pathways; service models; cost-effectiveness; PROMs/QoL; adherence/acceptance.

**Exclude:** pure diagnostic accuracy/measurement → 5; pathobiology/biomarkers → 3; treatment trials → 6/7/8/10/11/12.

**2) Microbiology and Biofilm Ecology**

**Definition:** microbial composition/dysbiosis, ecological shifts, microbial methods.

**Include:** microbial profile of disease/health; dysbiosis; biofilm architecture; virulence factor of pathogens;

**Exclude:** host-only biomarkers/pathways → 3; integrated host–microbe multi-omics → 13; implant disease therapy → 11.

**3) Host Response and Inflammatory Biomarkers**

**Definition:** immune/inflammatory pathways and biomarkers (GCF/saliva/blood/tissue).

**Include:** cytokines (IL-1β, TNF-α), MMPs, CRP; oxidative stress; host-side proteomics/metabolomics, inflammatory signaling pathways.

**Exclude:** pure microbiology → 2; broad multi-omics/genetics → 13.

**4) Systemic Links and Comorbidities**

**Definition:** bidirectional associations between periodontitis and systemic conditions.

**Include:** diabetes/HbA1c; CVD; pregnancy outcomes; osteoporosis; mediation/causality analyses

**Exclude:** local therapy outcomes without systemic endpoints

**5) Diagnostics and Measurement**

**Definition:** indices/thresholds; probing depth (PD)/clinical attachment level (CAL); radiographic/cone beam computed tomography (CBCT) as measurement; test validity/reliability; diagnostic AI/ML.

**Include:** ROC/AUC; sensitivity/specificity; examiner reliability; measurement repeatability; diagnostic ML

**Exclude:** planning/manufacturing workflows → 11 if implant-specific; implant placement/prosthetics → 11; biomaterial fabrication → 9.

**6) Nonsurgical and Resective/Access Periodontal Therapy**

**Definition:** tooth-related SRP/maintenance and non-regenerative surgical therapy.

**Include:** scaling/root planing; ultrasonic/air polishing; supportive periodontal therapy; access flaps; osseous recontouring; crown lengthening; modified Widman; root amputation

**Exclude:** antimicrobial-centered protocols → 7; tooth regenerative defects → 8; soft/hard-tissue augmentation → 10; implant therapy → 11.

**7) Antimicrobial and Adjunctive Strategies**

**Definition:** antibiotics/antiseptics/photodynamic/laser as antimicrobials.

**Include:** chlorhexidine; amoxicillin + metronidazole; doxycycline; photodynamic therapy (PDT); antimicrobial lasers; local antimicrobial delivery

**Exclude:** host-modulators (e.g., SDD as host-modulator, NSAIDs, statins) → 14; device/material science → 9; implant disease therapy → 11.

**8) Regenerative Periodontal Therapy**

**Definition:** regenerative therapies around natural dentition.

**Include:** GTR/membranes; EMD; rhPDGF; BMP-2; intrabony/furcation defects; histologic/regeneration outcomes.

**Exclude:** ridge/sinus augmentation for implant sites → 10; material-centric study → 9.

**9) Biomaterials and Biologics**

**Definition:** materials/biologics science independent of a single clinical technique.

**Include:** DFDBA/FDBA; xenografts; collagen membranes; PRF/PRP; handling/porosity/degradation; in vitro/in vivo characterization.

**Exclude:** technique-centric surgery → 6/8/10/11; cell-based TE → 12.

**10) Soft and Hard Tissue Augmentation**

**Definition:** mucogingival procedures and volume augmentation for implant/tooth sites.

**Include:** CTG/FGG/CAF; tunneling; phenotype/KT width; peri-implant soft-tissue augmentation; GBR; lateral/crestal sinus lift; ridge split; vertical/horizontal bone gain.

**Exclude:** tooth intrabony/furcation regeneration → 8; biomaterials characterization → 9; implant placement/prosthetics/maintenance → 11.

**11) Placement and Maintenance of Dental Implants**

**Definition:** implant timing/loading, guided/navigation surgery, prosthetic design, maintenance and peri-implant disease management; implant-specific planning workflows.

**Include:** immediate vs. delayed placement/loading; guided templates/navigation; emergence profile; implant-specific digital planning (CBCT-guided, intraoral scanning, surgical guide design) when tied to placement/prosthetics; peri-implant mucositis/peri-implantitis diagnostics/therapies; maintenance protocols and risk indicators.

**Exclude:** augmentation for volume → 10; pure diagnostic accuracy/measurement → 5; general biomaterials → 9.

**12) Tissue Engineering and Cell-Based Therapies**

**Definition:** cells/scaffolds/signals beyond conventional GTR.

**Include:** MSCs/PDLSCs; engineered scaffolds; bioreactors; cell-laden hydrogels; gene/cell therapies; growth factor + cells.

**Exclude:** conventional GTR/EMD without cells → 8; materials without cells → 9.

**13) Genetics, Epigenetics, and Multi-Omics**

**Definition:** genetic susceptibility, epigenetics, transcriptomics; integrated multi-omics and host–microbe networks.

**Include:** GWAS; SNPs; methylation; miRNA; RNA-seq; integrated host–microbiome analyses; pathway/network integration.

**Exclude:** single biomarker/pathway studies → 3; purely microbial taxonomy without host integration → 2.

**14) Host Modulation and Pharmacologic Interventions.**

**Definition:** drugs targeting host pathways/bone metabolism rather than bacterial kill.

**Include:** SDD as host-modulator; NSAIDs; statins (host effects); anti-resorptives' host effects; anti-inflammatory/osteomodulatory agents.

**Exclude:** antimicrobial protocols → **7**; observational systemic-link studies without intervention → 4.

**15) Miscellaneous/Unclassifiable**

**Definition:** use only if none of 1–14 reasonably fit after holistic review.

**Include:** truly out-of-scope items; cross-domain content that resists assignment.

**Exclude:** anything that can reasonably map to an above topic (keep this < 3% of corpus).

**A.2. Levels of Evidence Codebook**

**1) Level 1 *(High-quality RCTs or SRs; inception cohorts)***

**Definition:** high-quality single- or multi-center randomized controlled trials (RCTs) with narrow confidence intervals, or systematic reviews of these studies presenting homogeneity. Inception cohort studies or systematic reviews of these studies presenting homogeneity.

**Include: high-quality** RCTs; SRs of high-quality RCTs; inception cohort studies; SRs of inception cohorts.

**Exclude:** designs not meeting the above

**2) Level 2 *(Lower-quality RCTs; prospective cohort/comparative)***

**Definition:** lower-quality RCTs, prospective cohort or prospective comparative studies, or systematic reviews of these studies.

**Include:** lower-quality RCTs; prospective cohort; prospective comparative; SRs of these.

**Exclude:** Retrospective designs, case series/reports, expert opinion, pre-clinical.

**3) Level 3 *(Retrospective cohort/comparative; case–control)***

**Definition:** retrospective cohort or retrospective comparative studies, case–control studies, or systematic reviews of these studies.

**Include:** retrospective cohort; retrospective comparative; case–control; SRs of these.

**Exclude:** prospective RCTs/cohorts (Levels 1–2), case series/reports, expert opinion, pre-clinical.

**4) Level 4 *(Case series; poor-quality studies)***

**Definition:** case series, poor-quality cohort or case–control studies.

**Include:** case series; poor-quality cohort; poor-quality case–control.

**Exclude:** single case reports, expert opinion, pre-clinical.

**5) Level 5 *(Expert opinion; case report; narrative/editorials)***

**Definition:** expert opinions without critical appraisal, case report, case study, narrative reviews, editorials, commentaries.

**Include:** expert opinion; case report/case study; narrative reviews; editorials; commentaries.

**Exclude:** systematic reviews, analytic clinical studies, pre-clinical.

**6) Level 6 *(Animal/*in vitro*/*ex vivo*; pre-clinical)***

**Definition:** animal, in vitro (cellular/molecular), ex vivo (on non-vital or separated human organs), or pre-clinical studies.

**Include:** animal studies; in vitro; ex vivo; pre-clinical.

**Exclude:** human clinical/epidemiologic designs (Levels 1–5)

**Appendix S2**: Prompt Structure and Technical Setup

After finalizing the cleaned dataset (as described in Section 2.2), each study was assigned a unique identifier (e.g., JPR_0001), and its title, abstract, and author keywords were formatted as structured JSON text inputs. All tasks were implemented in Python (version 3.11) using the OpenAI API together with data-handling libraries including openai, pandas, tqdm, tenacity, json, pathlib, and re.

Each model interaction consisted of two components: a system message and a user message. The system message defined the model's role and general behavior (e.g., instructing the model to act as an expert periodontics researcher and strictly follow the corresponding codebook). The user message contained the task description, the full text of the relevant codebook (provided in Appendix A), and the formatted title, abstract, and keywords of the study. The model then returned its classification in a standardized JSON output structure.

To ensure reproducible results, a deterministic prompting configuration was used for both the pilot and full-scale analyses. Specifically, the model's randomness parameter (“temperature”) was fixed at 0 for GPT-4o and GPT-4o-mini, while a locked snapshot version (gpt-5-mini-2025-08-07) was used for GPT-5-mini. This setup ensured that identical prompts consistently produced identical outputs.

For the full dataset, the 4680 studies were randomly divided into 10 batches (each containing 468 records) and processed in two separate sessions for Topic and LEV labeling. Although not required for computational reasons, this batching approach allowed for consistent progress tracking and quality control across sessions. The random assignment of studies into batches (using a fixed random seed) ensured that each batch included papers from different publication years, preventing temporal clustering and maintaining balanced representation across decades.

The following sections (B.1 and B.2) present the exact prompt formats used for Topic and Level of Evidence (LEV) classification, along with representative examples of model inputs and outputs.

**B.1** Topic Labeling Prompt and Example

**Prompt structure**

**System Message**

“You are a careful periodontics domain expert and research assistant. Follow the JPR Topic Codebook exactly. Use holistic judgment from the title, abstract, and keywords. Return valid JSON only.”

**User message**

Task: Read Title, Abstract, and (if present) Keywords, and assign the BEST-FITTING topic from the JPR Topic Codebook.

• Choose ONE primary topic

• Use holistic judgment; do not rely on single trigger words.

• Return JSON ONLY with keys:

primary_id (int), primary_topic (str)

confidence (0–1), justification (≤ 30 words)

**Example** *(Paper ID: JPR_3995)*:

**Title:** “Histological changes in experimental periodontal disease in rats monoinfected with gram-negative organisms*”*

Keywords—**Abstract:** “Groups of weanling gnotobiotic rats of the Sprague-Dawley strain were monoinfected with isolates of *Cappocytophaga*, strains 4, 6 and 27, and with *Fusobacterium nucleatum*, strain 325, Bacteroides strain 289c and *Eikenella corrodens*, strain 1073. All caused periodontal disease between the first and second maxillary molars, with migration of the epithelial attachment, destruction of the alveolar bone by osteoclasts and impaction of debris. No plaque was formed. *Capnocytophaga*, strain 4, caused almost complete destruction of the periodontium by 42 days. The other organisms were less pathogenic, but all caused considerable destruction of the periodontium by 84 days”

**Model Output (JSON):**

{

"primary_id": 2,

"primary_topic": "Microbiology & Biofilm Ecology",

"confidence": 0.9,

"justification": "Animal monoinfection study assessing pathogenicity of gram-negative taxa and resulting histologic periodontal destruction (epithelial migration, osteoclastic bone loss)."

}

**B.2** Levels of Evidence Labeling Prompt and Example

**Prompt Structure**

**System Message**

“You are a careful periodontics domain expert and research assistant. Follow the Level of Evidence Codebook exactly. Use holistic judgment from the title, abstract, and keywords. Return valid JSON only.”

**User Message**

Task: Read Title, Abstract, and (if present) Keywords, and assign the BEST-FITTING Level of Evidence from the Codebook.

• Choose ONLY ONE level (1–6).

• Return JSON ONLY with keys:

level_id (int)

confidence (0–1)

**Example** *(Paper ID: JPR_3995)*:

**Title:** “Histological changes in experimental periodontal disease in rats monoinfected with gram-negative organisms*”.*

Keywords—**Abstract:** “Groups of weanling gnotobiotic rats of the Sprague-Dawley strain were monoinfected with isolates of *Cappocytophaga*, strains 4, 6 and 27, and with *Fusobacterium nucleatum*, strain 325, Bacteroides strain 289c and *Eikenella corrodens*, strain 1073. All caused periodontal disease between the first and second maxillary molars, with migration of the epithelial attachment, destruction of the alveolar bone by osteoclasts and impaction of debris. No plaque was formed. *Capnocytophaga*, strain 4, caused almost complete destruction of the periodontium by 42 days. The other organisms were less pathogenic, but all caused considerable destruction of the periodontium by 84 days”

**Model Output (JSON):**

{

"level_id": 6,

"confidence": 0.95

}
